# Environmental, economic, and social sustainability in aquaculture: the aquaculture performance indicators

**DOI:** 10.1038/s41467-024-49556-8

**Published:** 2024-06-20

**Authors:** Taryn M. Garlock, Frank Asche, James L. Anderson, Håkan Eggert, Thomas M. Anderson, Bin Che, Carlos A. Chávez, Jingjie Chu, Nnaemeka Chukwuone, Madan M. Dey, Kevin Fitzsimmons, Jimely Flores, Jordi Guillen, Ganesh Kumar, Lijun Liu, Ignacio Llorente, Ly Nguyen, Rasmus Nielsen, Ruth B. M. Pincinato, Pratheesh O. Sudhakaran, Byela Tibesigwa, Ragnar Tveteras

**Affiliations:** 1 School of Fisheries, Aquaculture and Aquatic Sciences, Auburn University, Auburn, AL 36849 USA; 2 School of Forest, Fisheries and Geomatics Science, University of Florida, Gainesville, FL 32611 USA; 3 Department of Safety, Economics and Planning, University of Stavanger, 4036 Stavanger, Norway; 4 Food and Resource Economics Department, University of Florida, Gainesville, FL 32611 USA; 5 Department of Economics, University of Gothenburg, 405 30 Göteborg, Sweden; 6 Department of Agricultural and Resource Economics, University of California at Davis, Davis, CA 95616 USA; 7 College of Economics and Management, Shanghai Ocean University, Shanghai, 201306 China; 8 Facultad de Economía y Negocios, Universidad de Talca and Interdisciplinary Center for Aquaculture Research, Talca, Chile; 9 East Asia-Environment, Natural Resources and Blue Economy, The World Bank, Washington, DC 20433 USA; 10 Department of Agricultural Economics and Resource and Environmental Policy Research Centre, Environment for Development Nigeria, University of Nigeria Nsukka, Nsukka, Enugu State 40001 Nigeria; 11 Department of Agricultural Sciences, Texas State University, San Marcos, TX 78666 USA; 12 Environmental Science, University of Arizona, Tucson, AZ 85721 USA; 13 Climate Resilient Fisheries and Oceans Program, Environmental Defense Fund, 1100 Quezon City, Philippines; 14 Ocean and water unit, European Commission Joint Research Centre, 21027 Ispra, Italy; 15 Thad Cochran National Warmwater Aquaculture Center, Delta Research and Extension Center, Mississippi State University, Mississippi, 38756 USA; 16 Business Administration Department, Universidad de Cantabria, 39005 Santander, Spain; 17 College of Agriculture and Food Sciences, Florida A&M University, Tallahassee, FL 32312 USA; 18 Department of Food and Resource Economics, University of Copenhagen, 1958 Frederiksberg C, Denmark; 19 University of Dar Es Salaam, Dar es Salaam, Tanzania; 20 UiS School of Business and Law, University of Stavanger, 4036 Stavanger, Norway

**Keywords:** Environmental economics, Environmental impact

## Abstract

Aquaculture is a rapidly growing food production technology, but there are significant concerns related to its environmental impact and adverse social effects. We examine aquaculture outcomes in a three pillars of sustainability framework by analyzing data collected using the Aquaculture Performance Indicators. Using this approach, comparable data has been collected for 57 aquaculture systems worldwide on 88 metrics that measure social, economic, or environmental outcomes. We first examine the relationships among the three pillars of sustainability and then analyze performance in the three pillars by technology and species. The results show that economic, social, and environmental outcomes are, on average, mutually reinforced in global aquaculture systems. However, the analysis also shows significant variation in the degree of sustainability in different aquaculture systems, and weak performance of some production systems in some dimensions provides opportunity for innovative policy measures and investment to further align sustainability objectives.

## Abstract (web summary)

Garlock and colleagues analyze 57 aquaculture systems worldwide on 88 metrics that measure social, economic, or environmental outcomes. They find significant variation in the degree of sustainability in different aquaculture systems, and weak performance of some production systems in some dimensions provides opportunity for innovative policy measures and investment to further align sustainability objectives.

## Introduction

Aquaculture is a recent addition to the global food production system, and due to rapid production growth, aquaculture has overtaken fisheries as the main source of seafood for human consumption^1^. Aquaculture has the potential to support livelihoods, food security, and human and environmental health^2–4^, but the industry is controversial as it is a new way of using aquatic resources, and there are significant concerns with respect to its environmental sustainability^5–8^ and its social impacts^9,10^. Lack of data has prevented systematic comparison of global aquaculture production systems. In this paper, we use data collected by the Aquaculture Performance Indicators (APIs; Supplementary Information) for 57 aquaculture systems to show that, on average, the three pillars of sustainability are complementary, suggesting no systematic trade-offs between economic, environmental, and social sustainability. The results indicate that the strength of the relationships between the sustainability pillars is quite different in aquaculture from fisheries, the other main production technology for seafood. In particular, there is a much weaker relationship between environmental and economic sustainability and a much stronger relationship between social and environmental sustainability. The APIs also facilitate the investigation of several controversial topics about aquaculture development. For instance, our results suggest that freshwater and marine aquaculture are equivalent from a sustainability perspective, and monoculture is preferable to polyculture. We identify high-performing aquaculture typologies and species and highlight opportunities to improve economic, social, and environmental performance. Our results support the nuanced picture of a heterogenous industry indicated by Naylor et al.^11^, and that a sustainable aquaculture industry is possible with the correct policy and investment decisions.

The concerns related to the sustainability of aquaculture often indicate that the three pillars of sustainability are competing rather than complementary. This is most evident in the potential trade-off between the environment and economic development. The aquaculture sector has clearly responded to economic opportunities to expand, intensify, and diversify^12,13^, and markets and trade are two important driving factors^14,15^. However, accompanying environmental externalities such as destruction of critical habitats, nutrient pollution, and the use of wild fish in feed production have been widely criticized^5,8^.

The potential trade-offs between the environment and aquaculture production are common issues that, at least in principle, can be addressed with improved governance^16,17^. For instance, mangrove forests can be protected from aquaculture development and are in many regions^18^, and forage fish stocks can be sustained with management systems that prevent overfishing^19^. Market incentives can also be important. For instance, rising prices for fishmeal and fish oil incentivized the industry to reduce fishmeal inclusion rates and explore alternative protein sources^7,11^. There is also increasing evidence that some forms of aquaculture perform well in terms of greenhouse gas emissions compared to other food production systems^4,20^.

The potential trade-offs between the economic and social sustainability of aquaculture are as controversial and even less studied. Aquaculture is mainly a food production technology used in the Global South, but market dynamics have affected the distribution of its benefits. Aquaculture is sometimes perceived to focus on the production of species and product forms that are aimed at the urban middle class and international markets^9^. In addition, it is argued the recent interest in developing marine aquaculture—operationalized through large-scale enterprises with highly automated systems—will contribute little to employment and food security among the poor^21^. It has also been shown that some development projects and business models, such as contract farming or sharecropping, designed to enhance economic opportunities of smallholder fish farmers worsen social equity by concentrating power and wealth among a few individuals^14,22,23^. Aquaculture has also been linked to labor exploitations in fishery value chains through the provisioning of feed inputs^24^.

On the other hand, several recent studies show that aquaculture can contribute to local economic development and poverty alleviation by increasing employment opportunities and reducing income inequality in low-income and rural areas by providing local ripple effects^25–28^. Increased aquaculture production can also increase the accessibility and availability of nutritious foods^29^. Belton et al.^2^ show that aquaculture is less export-oriented than wild-capture fisheries in the largest aquaculture-producing countries, and Garlock et al.^13^ show that increased aquaculture production increases domestic seafood consumption.

Despite the prevalence of potential sustainability trade-offs in the academic literature, there is increasing evidence that the complementarity indicated by the three pillars of sustainability model may apply also in aquaculture. We investigate these issues using a global dataset of 57 aquaculture systems spanning all populated continents. We first examine the relationships among the three pillars of sustainability and compare them to the same measures in fisheries, the other main seafood production method. We continue analyzing performance by production technology and species because some forms of aquaculture are more intensely criticized.

Assessing the three pillars of sustainability in aquaculture systems is challenging because comparable data are not available to investigate social, economic, and ecological outcomes in different aquaculture systems and regions. While good production data exist from the FAO, there is limited data available on environmental, economic, and social issues in many parts of the world, and this is particularly prevalent in developing nations. We address this challenge by developing a set of APIs that allows data to be collected consistently on a wide range of issues related to the three pillars of sustainability and, therefore, facilitates global comparisons. The APIs are an extension of the Fishery Performance Indicators (FPIs) of Anderson et al.^30^ and can be used in a similar fashion to assess specific issues with respect to sustainability globally^31,32^ or for specific systems or regions^33,34^. Several other sustainability metrics have been proposed for the aquaculture sector^35–37^. However, these metrics have a relatively specific focus and do not allow assessment along all three pillars of sustainability.

The APIs include 88 outcome measures grouped into 19 dimensions that can be further aggregated into the three indicators of environmental, economic, and social performance. The APIs also include 66 input metrics that reflect management approaches and enabling conditions. Each measure is scored on a 1 to 5 scale using data when they exist and are scored by an expert in data-poor settings (see Supplementary Information). Due to these differences in the scoring method, an uncertainty measure is also indicated with respect to the data quality. While indicators cannot fully capture all aspects of sustainability in relation to aquaculture, the large number of indicators used captures many of the most relevant aspects. In addition, the indicator system can be easily expanded to include specific issues that are of particular importance, as illustrated by McCluney et al.^33^ with the FPIs.

Data on 57 aquaculture systems were collected between 2020 and 2021 that reflect the performance of production systems as well as more general economic indicators in 2018 and 2019. As such, the data is not impacted by the COVID-19 pandemic and associated measures. The 57 case studies are an opportunistic sample targeting the major species and countries where aquaculture production takes place but are limited by where researchers volunteered to conduct APIs. The selected species and country combinations represent 41% of global aquaculture production. Case studies have been conducted in 21 countries which are responsible for 91% of global aquaculture production. Seventy-nine percent of the case studies are from developing countries, and this is consistent with the significant role of developing countries in global aquaculture production^38^.

**Fig. 1:**
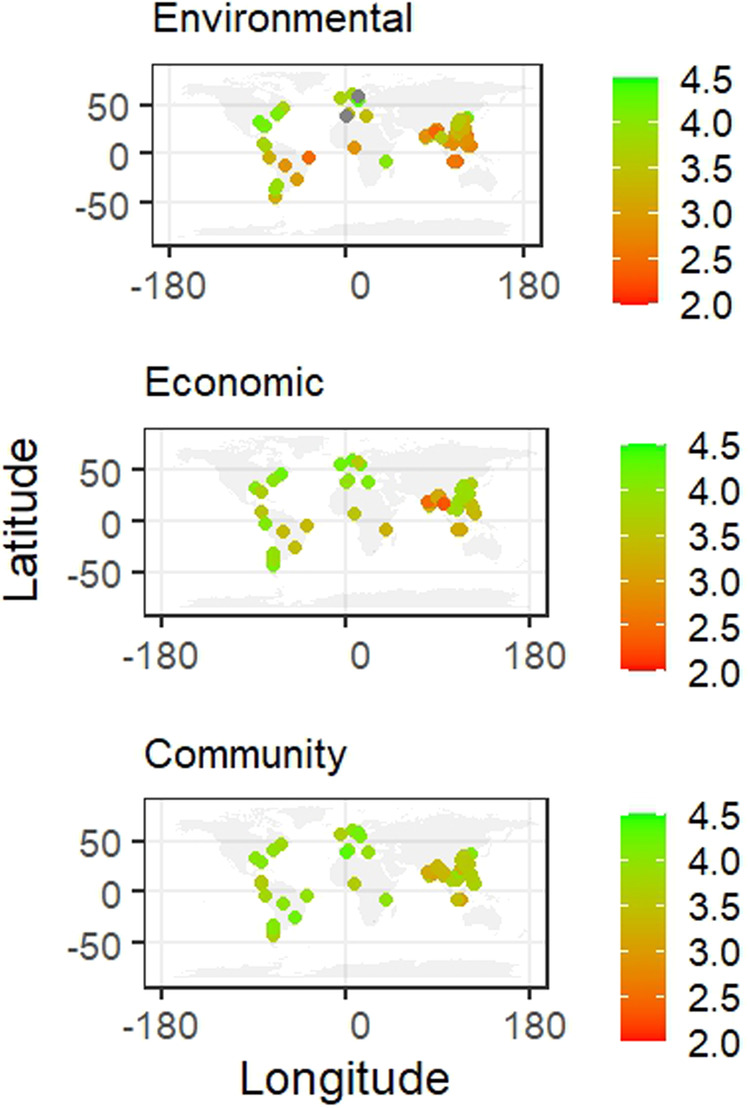
Geographical location, and environmental, economic, and social performance of aquaculture systems (*n* = 57). Green represents high scores, and red represents low scores.

Figure 1 maps the 57 aquaculture systems and their performance in the three pillars of sustainability. The aquaculture systems in the Global North scored higher in all three pillars compared to developing countries, although developing countries had a mix of well and poorly performing systems in all three pillars.

## Results

### Synergies and trade-offs among the three pillars of sustainability

**Fig. 2:**
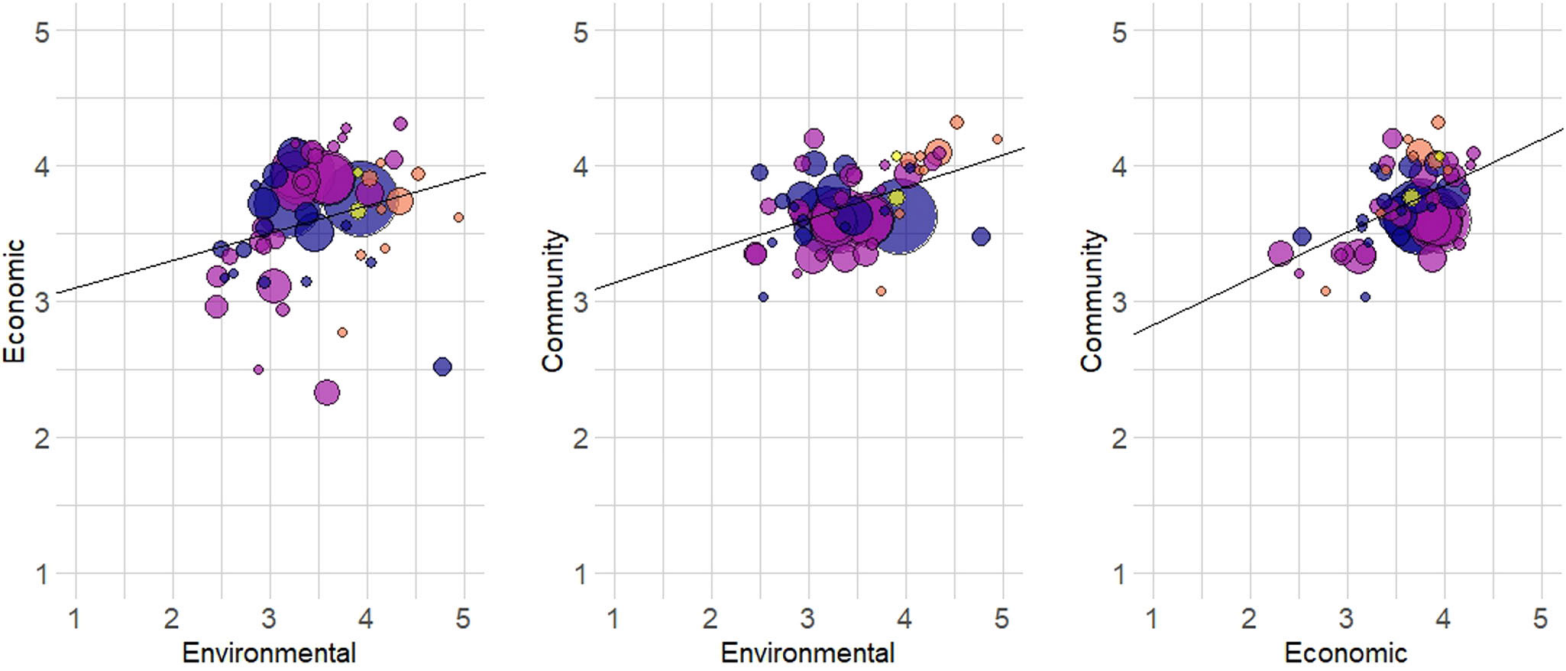
Correlations of the pillars of sustainability for 57 aquaculture systems. Correlation results (coefficient and *p* value) are as follows: Environmental—Economic (0.33, 0.012), Environmental—Community (0.45, <0.001), Economic—Community (0.55, <0.001). Crustaceans (blue), finfish (purple), mollusks (orange), seaweed (yellow), and the size of the bubble represents production value.

We investigated the relationship between the three pillars of sustainability by estimating correlations using 57 aquaculture case studies. The results indicated that all correlations were positive and statistically significant, suggesting that, on average, trade-offs do not exist among the pillars of sustainability (Fig. 2). The lack of trade-offs among the pillars of sustainability was analogous to the results reported by Asche et al.^31^ for global fisheries. However, the correlation coefficients for aquaculture were quite different from fisheries for two of the three relationships.

The correlation coefficient between the environmental and economic pillars was low at 0.33, and was lower than the 0.52 reported for fisheries^31^. This suggests that the environmental impacts of aquaculture were more weakly linked to the economic profits of the industry. This highlights an important distinction between fisheries and aquaculture wherein the environment plays a provisioning role in fisheries production and a supporting role in aquaculture production. The productivity of capture fisheries is intricately linked to habitat availability and quality, and thus destruction of habitat reduces the abundance and diversity of fish stocks for future capture. When fisheries are properly managed, the tragedy of the commons is mitigated, and the long-term economic benefits of healthy fish stocks are reaped by fishers^31,39,40^. In aquaculture, the farmer can influence the productivity of the system, and more so in intensive systems and species with closed production cycles, thus limiting dependence on the surrounding ecosystem. Therefore, degradation of the habitat through pollution or other means is not as tightly coupled to a farm’s productivity and profitability. This may also suggest that in some systems, aquaculture producers have weaker incentives to limit environmental externalities. As shown, e.g., by Pincinato et al.^41^, regulations may be the only way to limit some environmental externalities, although introducing market incentives such as ecolabels may also be an option^42,43^. However, it is worthwhile to note that the weaker correlation is driven by four outliers, India oysters, India carp, Myanmar carp polyculture, and Myanmar extensive shrimp. If these are removed, the correlation coefficient increases to 0.48. This is still lower than for fisheries, but not very much so.

The coefficient between environmental and social pillars was 0.45 and was considerably higher than the 0.23 coefficient reported for fisheries. The weak correlation for fisheries was not surprising given that social outcomes are dependent not only on the location of the stock, but where the fleet is based and where the fish is landed^31^. Aquaculture, on the other hand, occurs at a specific location, and at least some of the interactions must occur locally. Our results indicate that they do to a much larger extent than in fisheries. However, there are also commonalities as both fisheries and aquaculture are in part dependent on national, economic, and social policies^32,44^. The correlation between the economic and community pillars at 0.55 is slightly stronger than in fisheries and confirms that sustainable aquaculture is at least as well suited as fisheries to support community development objectives that seek to eradicate poverty and food insecurity. In aquaculture, greater control over the production process gives producers more leverage with respect to where the industry is located, and it is interesting to note the larger importance of aquaculture in inland regions^45^ and in highly populated countries^33^.

### Production environment, technologies, and species

**Fig. 3:**
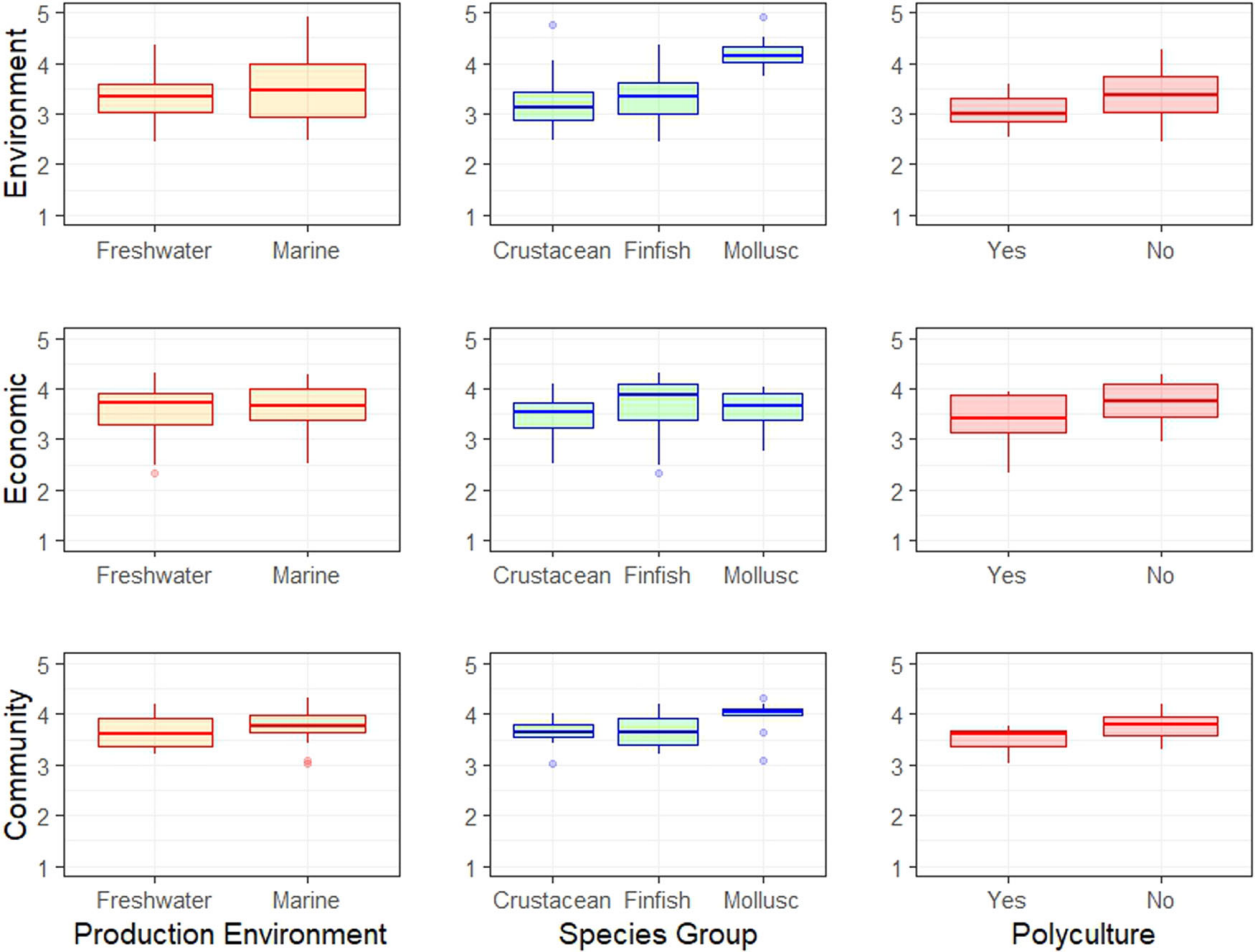
Relative performance in each of the three pillars of sustainability by production environment, species group, and mono- versus polyculture. Production environments are categorized into freshwater and marine environments, and species groups into crustaceans, finfish, and mollusks.

Because aquaculture production is highly diverse, and performance is expected to vary measurably by type, production technology, and species^11^, we further analyze the data by these factors (Fig. 3). Current aquaculture production is dominated by freshwater aquaculture^38^, but there are disparate views on future growth opportunities^45^. We find in aggregate that the performance of freshwater and marine aquaculture was similar in all three pillars (Fig. 3). Hence, from a sustainability perspective, the water bodies make little difference. This likely stems from the result that there are both positive and negative cases of marine and freshwater aquaculture around the globe. The result sheds light on potential dichotomies regarding freshwater and marine aquaculture ^21,46^ and has important policy implications for future development in the marine environment.

Based on our sample of aquaculture systems, monocultures on average scored higher in the three pillars than polycultures (Fig. 3). The finding that monocultures on average scored higher in the environmental pillar is at odds with existing views that multi-trophic aquaculture and polyculture can optimize resource use through utilization of wastes and byproducts^47^. However, the higher economic and social scores of monocultures were less surprising as the literature is more divided with respect to the social and economic benefits of polycultures (refs. ^10,48,49^).

**Fig. 4:**
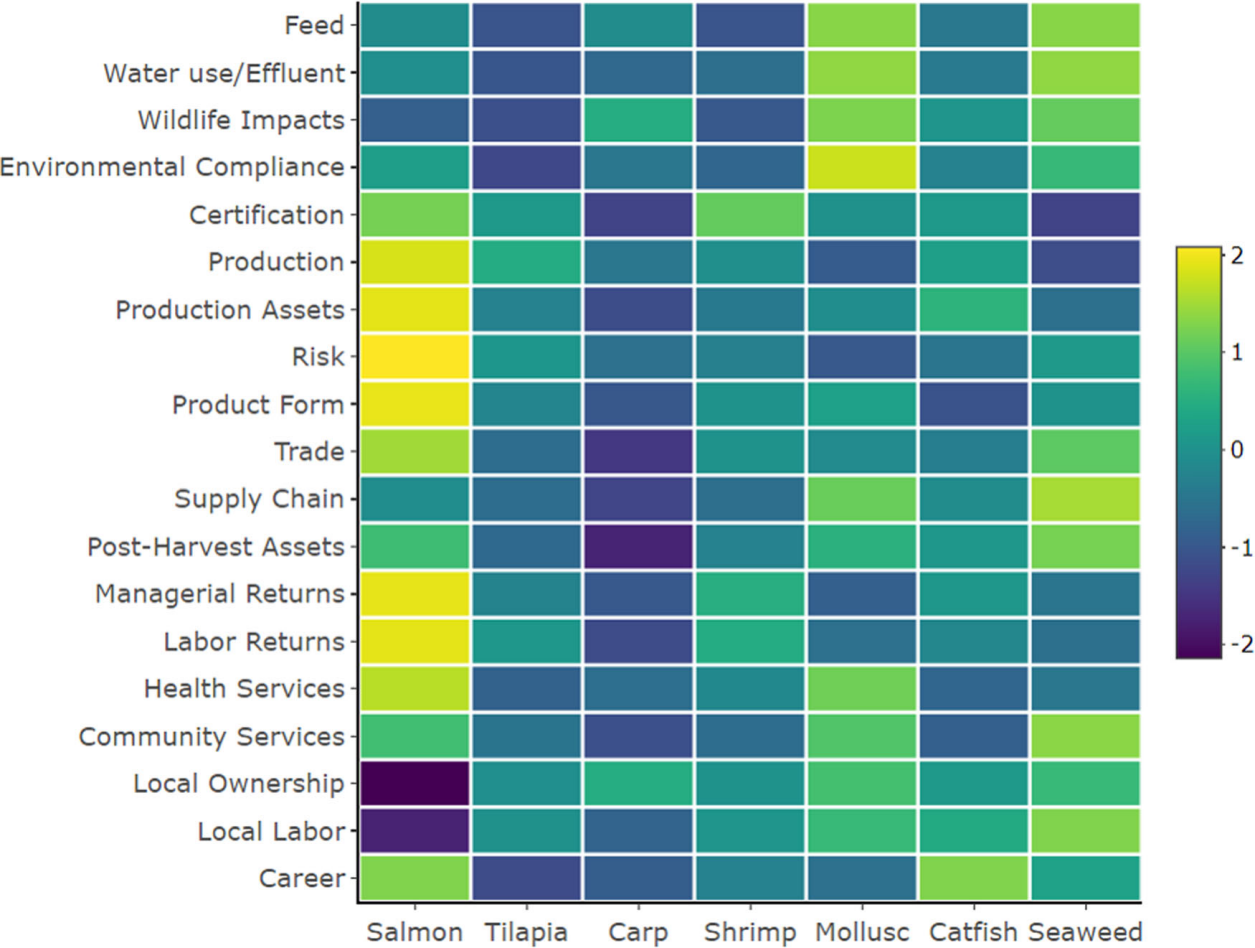
Average output dimension scores for salmon (*n* = 4), tilapia (*n* = 6), carp (*n* = 9), shrimp (*n* = 12), mollusk (*n* = 9), catfish (*n* = 4), and seaweed (*n* = 2). Scores are standardized by row by subtracting the mean and dividing by the standard deviation. Thus, the standardized score reflects the distance from the mean in units of standard deviation. See the Supplementary materials for the individual metrics comprising each dimension.

The relative performance of six important farmed species—salmon, tilapia, carp, shrimp, mollusks, and catfish—is shown in Fig. 4. Filter-feeding mollusks outperformed the other species in the environmental pillar including dimensions of feed use, water use, and effluent, and with respect to the broader impacts on native fish and the ecosystem. Molluscan aquaculture does not utilize manufactured feeds, and more importantly, fishmeal or fish oil which is one of the most heavily criticized aspects of aquaculture^50^. In addition, mollusks provide ecosystem services through assimilation of nutrients such as nitrogen and phosphorus as well as provision of habitat^51^. Mollusks, however, did not perform as well in the economics of production and were one of the riskiest forms of aquaculture. Control of production was the weakest due to susceptibility to environmental stresses and disease as well as the availability of quality seed^52–54^. Growth in global molluscan aquaculture has been slow relative to the growth observed in finfish. This is partly due to supply issues that have contributed to limited production and productivity growth^55^. Recent efforts to assign monetary values to the regulating ecosystem services provided by mollusks may provide economic incentives to increase production^51,56^.

Salmon aquaculture scored high in most dimensions, albeit with a few important exceptions. Salmon scored relatively high in feed performance similar to omnivorous carps which reflects that salmon feeds are generally sourced from sustainably managed fisheries and also that the use of marine ingredients have declined significantly^57^. Salmon performed better than other species in all but one of the economic dimensions, which reflects the success of global salmon farming. Technological innovations and productivity growth have led to intensive production with high degrees of control and automation at lower costs^12,58,59^. Salmon contrasted sharply with carp, which was the lowest-scoring species in many economic dimensions and is an artifact of production in extensive systems that produce low-value fish and weak profit margins. Salmon also performed well in most social dimensions reflecting high returns and wealth generation in the industry that is at least partly shared with the employees and the communities where the industry operates^25^. Salmon aquaculture scored low in the dimension of local labor and local ownership indicative of high foreign investment and ownership. This may be viewed as an artifact of a successful and globalized industry, whereas others believe it undermines local community development at the expense of their resources^60–62^. Interestingly, the greatest variation across species groups was observed in the dimension of local ownership, followed by trade, and the environmental dimensions of certification, feed, water use, and effluent. It should be noted that the scores presented compare the averages for different species groups, and there is significant variation in performance within each species group that is not shown here. For instance, Chile has had significant disease and environmental challenges with salmon production^63^, which has not been observed in other salmon-producing countries.

### International trade

**Fig. 5:**
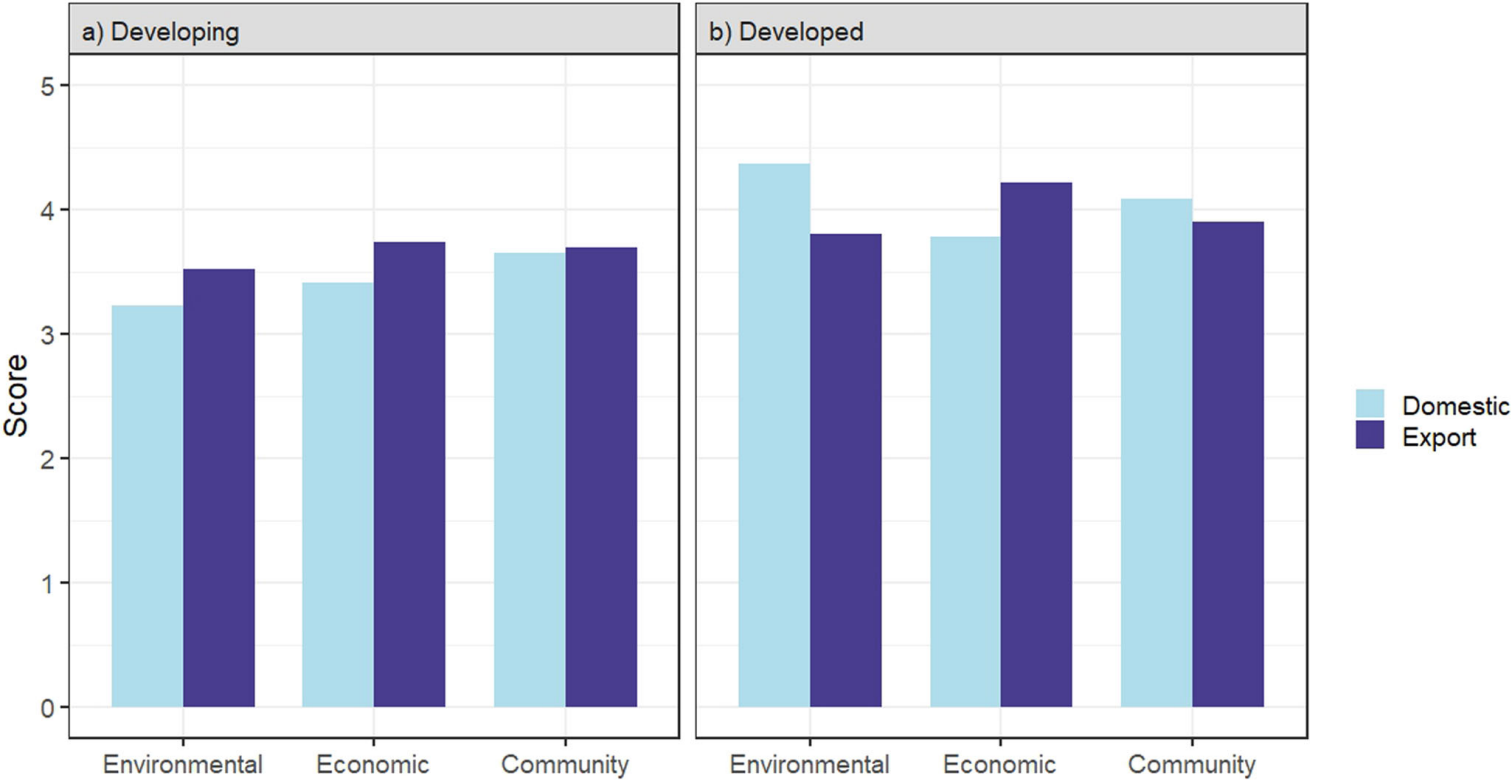
Average scores in the pillars of sustainability for domestic and export-oriented aquaculture. Each system is divided into **a** developing (domestic *n* = 27, export=13) and **b** developed countries (domestic = 6, export = 5).

The expansion of aquaculture is tightly coupled with the global trade of seafood commodities^15,64^, and international financial and development organizations have promoted export-oriented agro-industrialization to support economic growth and reduce poverty in many poor regions of the world^65^. Shrimp farming in Southeast Asia has been one of the most heavily scrutinized for its mangrove destruction and other environmental impacts, as well as the limited provisioning of socioeconomic benefits and food security to local communities relative to other forms of aquaculture that serve domestic urban markets^65–68^. We examine the performance of aquaculture production by market (i.e., domestic or export) and by development status (Fig. 5). In low-income countries, export-oriented aquaculture systems outperformed production for domestic markets in environmental (*t*(32) = 2.206, *p* = 0.035; Fig. 5a) and economic performance (*t*(28) = 2.658, *p* = 0.013; Fig. 5a) whereas community performance did not differ between domestic and export-oriented aquaculture (*t*(22) = 0.410, *p* = 0.686; Fig. 5a). This contrasts with specific case studies in the literature suggesting that export-oriented aquaculture results in greater environmental degradation^67^. The better environmental performance of export-oriented sectors may suggest that the export industry is improving in response to market signals.

We find the reverse trend in high-income countries where aquaculture systems serving domestic markets scored higher in the environmental pillar than export-oriented aquaculture (*t*(8) = −2.829, *p* = 0.0210; Fig. 5b). Better economic performance in export-oriented aquaculture was also found in high-income countries (*t*(6) = 3.945, *p* = 0.008; Fig. 5b) but community performance did not differ between domestic and export-oriented aquaculture (*t*(8) = −2.083, *p* = 0.070; Fig. 5b). The results support the notion that production for export and domestic markets are linked to different performance outcomes, and that the dynamics are further nuanced by development status. Given that a large share of the aquaculture production exported from developing countries is destined for wealthy markets suggests that market incentives exist and offset the cost of more sustainable production practices. However, the market incentives are, on average, lacking for production serving domestic markets in the developing world.

## Discussion

Aquaculture production is rapidly growing, but it is also a heavily criticized food system as environmentally and socially unsustainable practices are perceived to arise in pursuit of economic objectives. This study utilized data collected with the APIs for 57 aquaculture systems and depicted that on average the three pillars of sustainability are complementary, suggesting no systematic trade-offs between economic, environmental, and social sustainability. Hence, sustainable aquaculture production is possible, and fundamental trade-offs among economic, ecological, and social sustainability should not be viewed as the norm, even though they may exist in specific cases. However, the results also indicate that there is significant variation in the degree of sustainability in different aquaculture systems, supporting the observation of Naylor et al.^11^ that it is a highly heterogeneous industry.

The heterogeneity is highly interesting, and our results indicate that some important debates most likely are over-simplified. This is most obvious in the discussion on the relative merits and potential of freshwater versus marine aquaculture^21,45,46^. Our results indicate that this is not a particularly interesting distinction, as the systems are performing quite similarly in all three sustainability dimensions. Rather, there are other factors that seem to be more important, such as the difference between mollusks and finfish or the degree of control over the production process.

Aquaculture production systems vary widely in terms of space, production technology, species, and market. The identification of negative outcomes in some species and dimensions serves not as a rejection of the food production technology, but to inform policy and investment decisions. Our analyses highlight and compare different aspects of sustainability for different forms of aquaculture and can be used to identify areas for improvement and key areas of needed research. The data also provides a baseline of the average performance of the sector as well as for different species groups. However, these analyses are just the beginning, and continued development of the APIs database will facilitate analysis on more detailed questions and will significantly help improve the sustainability of the aquaculture sector. An expansion of the database will be beneficial as it will allow analysis of specific sub-systems and make the sample more representative of global aquaculture. For instance, additional observations for seaweed would facilitate analysis of one of the most rapidly growing parts of the aquaculture sector.

## Methods

The APIs are designed to assess the performance of aquaculture systems. An aquaculture system consists of a group of aquaculture producers in a similar environmental area, production technology, and management structure. Aquaculture systems can be local, national, or multinational but will most often be an industry producing one species in one country.

There are two types of performance indicators: outputs and inputs. Output indicators reflect the system’s environmental, economic, and social outcomes. There are 88 output indicators scored on a scale of 1–5, with categories defined to capture global variation. Hence, rather than measuring a few indicators, the APIs scored multiple metrics with various degrees of precision. The output indicators are grouped into 19 output dimensions with multiple indicators within each dimension to reduce the effect of potential mismeasurement and to triangulate more accurate values. The 19 output dimensions are further aggregated to capture performance in the three pillars of sustainability: environmental, economic, and social performance (Supplementary Information). Environmental performance is captured in five dimensions reflecting feed use, water use and effluent, and broader impacts on native fish and the ecosystem. Economic performance is measured by whether the aquaculture system is generating market benefits and is determined by factors such as farm-gate and wholesale prices, international trade, and supply chain efficiency. Social performance reflects the extent to which aquaculture contributes to livelihoods and other benefits in the community and is determined by farmer and processor wages and access to community services.

The second type of API indicator is the input indicator. There are 66 input metrics scored on a scale of 1–5 that reflect the management approaches and enabling conditions of the system, such as macroeconomic conditions, property rights, regulation, research and development, and infrastructure (Supplementary Information). The input measures are designed to inform on their impact on environmental, economic, and social performance indicators, and thus it is not assumed that higher scores for input metrics are better.

API assessments have been conducted for 57 aquaculture systems (Supplementary Information) between 2020 and 2021 that represent 41% of global aquaculture production quantity and 37% of global production value. The 57 assessments represent a non-random sample where opportunistic sampling was used to collect data on sectors with relatively high importance in global aquaculture production and in locations where we could find collaborators. However, there were important sectors that were not within our capacity to assess. Assessments have been conducted in all but one of the top ten aquaculture-producing nations. Egypt is absent, as it was not within our capacity to collect data there. Assessments have also been conducted for about 40 species or species groups, and these species are responsible for 58% of global production quantity and 67% of value. Fifty percent of the sample are finfish, 31% crustacean, 16% mollusks, and 3% seaweed. For comparison, global aquaculture production is 47% finfish, 28% seaweed, 15% mollusks, and 9% crustaceans. Hence, seaweed aquaculture is the species group most underrepresented in our sample, and with only two observations, a separate analysis could not be conducted for this sector. It is worthwhile to note that as most of the seaweed production has a low unit value, its value share is only 5%, due to the fact that most of the production is for non-food industrial uses. It is also important to note that some small sectors are present largely because the data was easy to obtain. The main results are quite stable as the removal of outliers does not change conclusions.

FPI assessments were conducted for 121 fishery sectors between 2011 and 2017 and are reported on in ref. ^30,31^. We do not believe that the different time periods when the FPI and API data were collected should influence the comparison as the indicators are assessing structural issues that are not likely to change significantly over relatively short time spans.

Each assessment is led by a scorer who identifies the best available source of information for each metric for a single year. For indicators covering several years, this year will be the final year. The scorer can draw on targeted data, proxy data, interviews with farmers, and local expert knowledge when data are not available. To minimize interscorer variation, there is an extensive manual that provides justification and detailed examples for scoring each metric (Supplementary Information). At least one experienced API analyst has vetted all assessments independent of the scorer to ensure consistency. The focus of a specific year can be a challenge when a sector is rapidly changing. For instance, when our observation of shrimp in Indonesia was conducted, the industry was primarily composed of extensive producers, but it has intensified rapidly since then.

While the categories in most cases are clear, there are also several cases where the boundaries are fluent. In some cases, this is explicitly scored. For instance, to examine potential differences in domestic and export-oriented aquaculture sectors, we separate the data based on the International Trade metric. Those sectors receiving a score of 1 (virtually no export) and 2 (2–30% export) were classified as domestic, and those sectors receiving a score of 4 (60–90% export) and 5 (90–100% export) were classified as export-oriented. Those sectors scoring a 3 (30–60% export) were not included in this analysis. Other categorizations are a part of the sectors’ description, such as monoculture versus polyculture. Here, the classification is based on the main product so that a sector will be classified as polyculture only if there is more than one species produced to be sold or used in subsistence settings. Species that are maintained to provide environmental or fish health services in the production process are not regarded as polyculture. For some sectors, like Chinese carp culture, this is common practice, and the systems scored here were primarily producing one species for the market and, therefore, are categorized as monoculture; however, there are other producers in the sector that are polyculture. In the Indonesian tilapia/shrimp system, the shrimp is mostly exported, while the tilapia is consumed locally. Production indicators are then an average of the two species. For example, the export indicator is scored as a 3.

### Reporting summary

Further information on research design is available in the Nature Portfolio Reporting Summary linked to this article.

### Supplementary information

Supplementary Information

Peer Review File

Reporting Summary

## Data Availability

The data that support the findings of this study is available at https://github.com/taryngarlock/API-Data-2023.
