# Author Correction: Environmental, economic, and social sustainability in aquaculture: the aquaculture performance indicators

**DOI:** 10.1038/s41467-024-50360-7

**Published:** 2024-07-16

**Authors:** Taryn M. Garlock, Frank Asche, James L. Anderson, Håkan Eggert, Thomas M. Anderson, Bin Che, Carlos A. Chávez, Jingjie Chu, Nnaemeka Chukwuone, Madan M. Dey, Kevin Fitzsimmons, Jimely Flores, Jordi Guillen, Ganesh Kumar, Lijun Liu, Ignacio Llorente, Ly Nguyen, Rasmus Nielsen, Ruth B. M. Pincinato, Pratheesh O. Sudhakaran, Byela Tibesigwa, Ragnar Tveteras

**Affiliations:** 1 School of Fisheries, Aquaculture and Aquatic Sciences, Auburn University, Auburn, AL 36849 USA; 2 School of Forest, Fisheries and Geomatics Science, University of Florida, Gainesville, FL 32611 USA; 3 Department of Safety, Economics and Planning, University of Stavanger, 4036 Stavanger, Norway; 4 Food and Resource Economics Department, University of Florida, Gainesville, FL 32611 USA; 5 Department of Economics, University of Gothenburg, 405 30 Göteborg, Sweden; 6 Department of Agricultural and Resource Economics, University of California at Davis, Davis, CA 95616 USA; 7 College of Economics and Management, Shanghai Ocean University, Shanghai, 201306 China; 8 Facultad de Economía y Negocios, Universidad de Talca and Interdisciplinary Center for Aquaculture Research, Talca, Chile; 9 East Asia-Environment, Natural Resources and Blue Economy, The World Bank, Washington, DC 20433 USA; 10 Department of Agricultural Economics and Resource and Environmental Policy Research Centre, Environment for Development Nigeria, University of Nigeria Nsukka, Nsukka, Enugu State 40001 Nigeria; 11 Department of Agricultural Sciences, Texas State University, San Marcos, TX 78666 USA; 12 Environmental Science, University of Arizona, Tucson, AZ 85721 USA; 13 Climate Resilient Fisheries and Oceans Program, Environmental Defense Fund, 1100 Quezon City, Philippines; 14 Ocean and water unit, European Commission Joint Research Centre, 21027 Ispra, Italy; 15 Thad Cochran National Warmwater Aquaculture Center, Delta Research and Extension Center, Mississippi State University, Mississippi, 38756 USA; 16 Business Administration Department, Universidad de Cantabria, 39005 Santander, Spain; 17 College of Agriculture and Food Sciences, Florida A&M University, Tallahassee, FL 32312 USA; 18 Department of Food and Resource Economics, University of Copenhagen, 1958 Frederiksberg C, Denmark; 19 University of Dar Es Salaam, Dar es Salaam, Tanzania; 20 UiS School of Business and Law, University of Stavanger, 4036 Stavanger, Norway

**Keywords:** Environmental economics, Environmental impact

Correction to: *Nature Communications* 10.1038/s41467-024-49556-8, published online 20 June 2024

In this article the affiliation details for Madan M. Dey and Pratheesh O. Sudhakaran were incorrectly given as ‘College of Agriculture and Food Sciences, Florida A&M University, Tallahassee, FL 32312, USA’ but should have been ‘Department of Agricultural Sciences, Texas State University, San Marcos, TX, 78666, USA’.

In addition, the affiliation details for Ly Nguyen were incorrectly given as ‘Department of Agricultural Economics, Texas A&M University, McAllen, TX 78504, USA’ but should have been ‘College of Agriculture and Food Sciences, Florida A&M University, Tallahassee, FL 32312, USA’.

The original article has been corrected.

