# Supplementary material for: Environmental, economic, and social sustainability in aquaculture: the aquaculture performance indicators
Source: Nat Commun. 2024 Jun 20;15:5274. doi: 10.1038/s41467-024-49556-8 (PMC11190207; doi:10.1038/s41467-024-49556-8)
Supplement: Supplementary file 1 — Supplementary Information [file 41467_2024_49556_MOESM1_ESM.pdf]

Supplementary Information

Aquaculture Performance Indicators Manual

Version 1.0

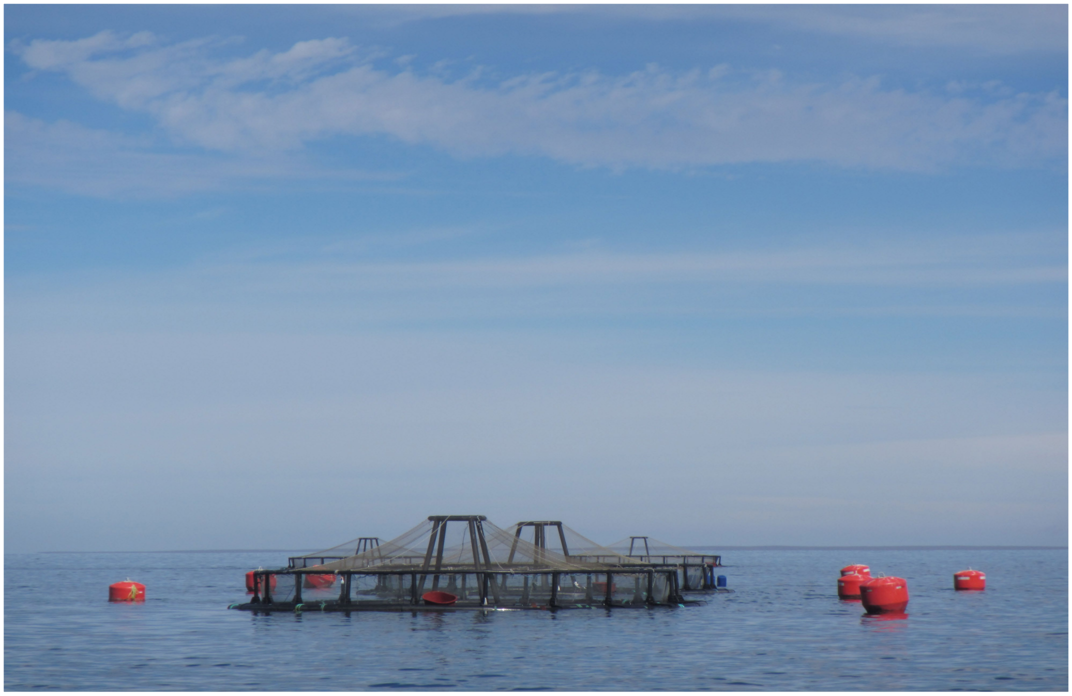

James L. Anderson[1], Frank Asche[2], Taryn Garlock[3], Håkan Eggert[4]

December 2, 2020

Citation: Anderson, J.L., Asche, F., Garlock, T. and Eggert, H. 2020. Aquaculture Performance Indicators Manual (version 1.0).

[1] Director, Institute of Sustainable Food Systems, Professor, Food and Resource Economics, University of Florida, Gainesville, FL, Email: james.anderson@ufl.edu
[2] Professor, Fisheries and Aquatic Sciences, University of Florida, Gainesville, FL, Email: frank.asche@ufl.edu
[3] Assistant Research Scientist, Institute for Sustainable Food Systems, University of Florida, Gainesville, FL, Email: tgainer@ufl.edu
[4]Associate Professor, Department of Economics, University of Gothenburg, Gothenburg, Sweden, Email: hakan.eggert@economics.gu.se

CONTENTS

INTRODUCTION....3

PRACTICAL SCORING GUIDANCE....4

AQUACULTURE PERFORMANCE INDICATORS — Outputs....5

AQUACULTURE PERFORMANCE INDICATORS — Inputs....105

APPENDIX: CASE STUDIES....173

TABLES

Table 1. AQUACULTURE PERFORMANCE INDICATORS — Outputs....6

Table 2. AQUACULTURE PERFORMANCE INDICATORS — Inputs....106

INTRODUCTION

This report presents an innovative set of Aquaculture Performance Indicators (APIs) for evaluating and comparing the world's aquaculture management systems. These indicators build upon the success of the Fishery Performance Indicators (FPIs) and their effectiveness in evaluating investment decisions and fisheries regulatory institutions in ecosystem health, economic sustainability and human well-being (Chu et al. 2017; Asche et al. 2018; McCluney et al. 2019). The FPIs have been supported and used by many organizations including the World Bank, World Wildlife Fund, Environmental Defense Fund, the Wildlife Conservation Society and the Packard Foundation (Anderson et al. 2013; Chu et al. 2012; Chu and Meredith 2015), and organizations have identified the need to have an analogous tool for aquaculture management systems.

The APIs adopt a similar framework as the FPIs and are composed of two types of indicators – outputs and inputs. The first type of indicators are output indicators that identify and measure whether the aquaculture sector is economically viable and socio-ecologically sustainable. In accordance with the World Bank's emphasis on the triple bottom line and the importance of an integrated ecosystem management approach, each of the API outcomes corresponds with environmental, economic, or community well-being. Additionally, each API output metric corresponds to the environmental, production, or post-production sector.

The second type of indicators are input indicators, or indicators of enabling conditions, that contribute to the process of incentivizing profitable and socio-ecologically sustainable aquaculture industries. By analyzing relationships among the output and input metrics, the API dataset can be used to understand the causes, correlations and paths toward successful and sustainable industry development that can arise from investment in, and changes to, aquaculture policy and practice. This will give academics, policy makers and industry participants critical information to make a case for better management based on a broader set of criteria incorporating governance and economic factors, many of which are currently being ignored.

PRACTICAL SCORING GUIDANCE

COMPLETING THE API WORKSHEET IN EXCEL

1. Fill in the first tab of the worksheet "Cover Page" with country, location, production technology, species name, date, and author information.

2. It is essential to fill in the column of quality scores for both the input and output tables. Note the quality score guidelines:

    A: Reviewer is highly confident (95%) the 1-5 score is correct. Confidence can come from familiarity with the aquaculture sector, the reliability of another expert source, a calculation based on reliable data, or large ranges of the underlying metric for the given score that make another score highly unlikely for the sector. Note that it is confidence in the 1-5 score that matters, and thus wide ranges for the underlying metric associated with a score can support "A" quality, even in the case when information about the precise level of the underlying metric is poor.

    B: Reviewer feels 1-5 score is more likely than others, and reviewer is highly confident (95%) that the true underlying metric would be within one of the given score.

    C: Reviewer is making an educated guess based on best available information, but reviewer is not highly confident the true metric would be within one of the given score.

    Note that uncertainty about the interpretation of the metric should be resolved through consultation with the FPI materials or personnel, rather than giving the score a lower quality. Interpretations can be explained in the notes.

3. All metrics should be scored with a 1, 2, 3, 4, 5 or NA. Intermediate scores of 1.5, 2.5, etc. are not acceptable. NA is only acceptable if the metric truly does not apply to the aquaculture sector (example: in a sector with no property rights you should score the transferability of the property right as NA). If a score cannot be given due to missing data, the metric should not be left blank: an educated guess as to the score should be made and the metric should be given a quality score of C.

4. The explanation column should be filled in for each metric so that reviewers know the rationale behind the given score. Explanations can be brief but it should be clear from the explanation and data source column which information sources are being used.

5. An API Profile should be completed for each aquaculture sector in order to provide important context and background information for the scores. Completion of the aquaculture profile does not mean that scores no longer require an explanation in the worksheet.

AQUACULTURE PERFORMANCE INDICATORS

Outputs

MEASURING ENVIRONMENTAL, ECONOMIC, AND COMMUNITY OUTCOMES

This section identifies 88 metrics of environmental health and human well-being (see Table 1 below). Each metric (found in the third column in Table 1) is individually explained in the following pages. To facilitate scoring, the metrics are organized in the manual according to the sector partitioning, as data on each sector tends to be available from similar sources. Each indicator is presented alongside practical scoring guidance and examples that are derived from the existing set of case studies or from theoretical situations that could arise.

Supplementary Table 1. Aquaculture Performance Indicators—Outputs

Dimension	Component	Metric
Environmental Health	Feed-related impacts	Sustainability of aquatic feed sources
		Sustainability of non-marine feed ingredients
	Water use and effluents	Impact of discharge (nutrient emissions)
		Non-nutrient emissions
		Freshwater use
	Impacts to wildlife	Wildlife mortality
		Benefits to wildlife
		Ecological impacts of escaped fish
		Genetic impacts of escaped fish
		Parasite and disease transmission
		Site use
		Land Use
		GHG emissions
	Environmental compliance	Compliance with environmental law
	Certification	Proportion of production with 3rd party certification
Production Sector	Production Performance	Production Technology
		Adult feed
		Juvenile survival rate
		Juvenile production
		Selective breeding and production time
		Survival Trend
		Survival Rate
		Proportion of production affected by disease and parasites
		Proportion of production affected by predation
		Proportion of production that escapes
		Proportion of production lost to handling and unspecified loss
		Production costs compared to historic high
	Production Assets	Ratio of Asset Value to Gross Earnings
		Total Revenue Compared to Historic High
		Asset (Permit, Quota, etc...) Value Compared to Historic High
		Borrowing Rate Compared to Risk-free Rate
		Source of Capital
		Functionality of Production Capital
	Risk	Annual Total Revenue Volatility
		Annual Production Volatility
		Intra-annual Production Volatility
		Annual Price Volatility
		Intra-annual Price Volatility
		Spatial Price Volatility
		Contestability & Legal Challenges
	Farm Owners	Earnings Compared to Regional Average Earnings
		Owner Wages Compared to Non-Aquaculture Wages
		Education Access
		Access to Health Care
		Social Standing of Farm Owners
		Proportion of Nonresident Owners
	Farm Workers	Earnings Compared to Regional Average Earnings
		Worker Wages Compared to Non-Aquaculture Wages
		Education Access
		Access to Health Care
		Social Standing of Workers
		Proportion of Nonresident Workers
		Worker Experience
		Age Structure of Workers
		Proportion of income spent on food
Post-Harvest Sector	Markets	Farm-gate Price Compared to Historic High
		Final Market Use
		International Trade
		Final Market Wealth
		Wholesale Price Compared to Similar Products
		Capacity of Firms to Export to the US & EU
		Farm-gate to Wholesale Marketing Margins
		Food safety
	Supply Chain Performance	Processing Yield
		Shrink
		Capacity Utilization Rate
		Product Improvement
		Proportion of production sold fresh
		Sanitation
		Local Support Businesses
		Availability of Support Businesses
		Proportion of feed ingredients sourced from socially responsible sectors
	Post-Harvest Assets	Borrowing Rate Compared to Risk-free Rate
		Source of Capital
		Age of Facilities
	Processing Managers	Earnings Compared to Regional Average Earnings
		Manager Wages Compared to Non-fish farming Wages
		Education Access
		Access to Health Care
		Social Standing of Processing Managers
		Nonresident Ownership of Processing Capacity
	Processing Workers	Earnings Compared to Regional Average Earnings
		Worker Wages Compared to Non-fish farming Wages
		Education Access
		Access to Health Care
		Social Standing of Processing Workers
		Proportion of Nonresident Employment
		Worker Experience

ENVIRONMENTAL HEALTH

FEED-RELATED IMPACTS

Sustainability of Aquatic Feed Resources

RATIONALE:
Sustainably sourced ingredients for aquafeeds is an essential component to environmentally sustainable aquaculture. It is hypothesized that increased aquaculture production will increase demand for and exploitation of wild capture fisheries for fishmeal in aquafeeds and consequently threaten the sustainability of wild capture fisheries (Naylor et al. 2000; Deutsch et al. 2007). The effect of aquaculture production on exploitation of feed fisheries is in large part dependent on the management system for feed fish given depletion of feed fish stocks can be everted if management is in place to prevent overexploitation. Feed ingredients sourced from unsustainable fisheries also represent a source of risk and uncertainty for the aquaculture sector.

METRIC:
Measures the environmental sustainability of marine ingredients (fish oil and fish meal) in the feed. This should be scored to reflect the feed for the dominant portion of production value.

5	Feeds are sourced from BAP or other certified feed mills, or no marine ingredients are used
4	Fishery-based ingredients are sourced from sustainably managed fisheries
3	Some fishery-based ingredients are sourced from sustainable fisheries
2	Fishery-based ingredients are sourced from unmanaged fisheries or managed fisheries with severely depleted stocks
1	Fishery-based ingredients are sourced from unsustainable and severely overfished stocks

SCORING GUIDANCE:
If fish meal and fish oil are not used in the feeds or if there are no feed inputs, then the score should be a 5. Note: This should be scored for growout stages, not seed production.

EXAMPLES:
1. All feed mills in the U.S. catfish industry are BAP certified, thus scoring a 5.
2. Fishmeal and fish oil found in Atlantic salmon feeds are sourced from sustainable fisheries and byproducts, but feed mills do not have an internationally recognized sustainability certification, thus scoring a 4.

ENVIRONMENTAL HEALTH

FEED-RELATED IMPACTS

Sustainability of Non-Marine Feed Ingredients

RATIONALE:
Sustainably sourced ingredients for aquafeeds is an essential component to environmentally sustainable aquaculture. Terrestrial plant sources are commonly asserted as sustainable alternatives to marine ingredients. Recent findings challenge this notion that exchanging marine ingredients with terrestrial crops will necessarily minimize environmental impacts (Malcorps et al. 2019). Aquaculture that sources feed ingredients from farms with best management practices will minimize the environmental footprint.

METRIC:
Measures the environmental sustainability of non-marine ingredients in the feed. This should be scored to reflect feeds for the dominant portion of production value.

5	Feeds are sourced from high yield farming with best management farming practices, or no non-marine ingredients are used
4	Feeds are sourced from farms with yields more than 20% higher than global averages
3	Feeds are sourced from farms with yields within 20% of global averages
2	Feeds are sourced from farms with yields less than 80% of global averages
1	Feeds are sourced from farms with extremely eroded soils

SCORING GUIDANCE:
If non-marine ingredients are not used in the feed or there are no feed inputs, then the score should be a 5. Note: This should be scored for growout stages, not seed production.

EXAMPLES:

ENVIRONMENTAL HEALTH
WATER USE AND EFFLUENTS

Impact of Discharge (Nutrient Emissions)

RATIONALE:
Aquaculture facilities commonly discharge water and unused nutrients into surrounding environments (primarily as nitrogen and phosphorus) which can lead to eutrophication of aquatic environments. On the other hand, some aquaculture production may have a net positive impact on the environment through bioassimilation of nutrients. Examples of aquaculture with positive impacts on water quality are oysters, mussels, clams and seaweed (Lindahl et al. 2005, Higgins et al. 2011).

METRIC:
Measures the observable impact of discharged nutrient emissions (mainly nitrogen and phosphorus) on the ecosystem.

5	Positive impact on water quality
4	No observable impact
3	Minor impact; minor observable effect on primary production
2	Significant impact; observable effects on primary production and aquatic animals
1	Severe eutrophication resulting in hypoxic conditions

SCORING GUIDANCE:
Production of some organisms (such as bivalves and seaweeds) can assimilate nutrients rather than release nutrients; these sectors should score a 5. Note: This should be scored for growout stages, not seed production.

EXAMPLES:
1. In the case of U.S. catfish, pondwater is exchanged every 10-15 years and there is no significant change in water quality parameters in nearby ecosystems following discharges, thus U.S. catfish scores a 4.

ENVIRONMENTAL HEALTH

WATER USE AND EFFLUENTS

Non-Nutrient Emissions

RATIONALE:
The use of chemicals and biological agents in aquaculture production is common, similar to other food production systems, to prevent and treat diseases, enhance the health of the organism and improve the environmental rearing conditions. Consequently, considerable amounts of chemicals and biological products used in production can be released, either intentionally or not, into the surrounding environment and poses risks for the health and function of the surrounding ecosystem and can contribute to the development of resistant strains of bacteria and parasites (Rico et al. 2012). Many factors influence the level of non-nutrient emissions ranging from use, application methods, discharge and water treatment practices.

METRIC:
Measures the level of non-nutrients detected in the discharge environment and/or environment surrounding the farm. Chemicals (i.e., pesticides and hormones), antimicrobials, probiotics, etc. should be considered in this measure.

5	Zero non-nutrients detected in the surrounding environment
4	Trace amounts of non-nutrients detected
3	Moderate amounts of non-nutrients detected
2	High levels of non-nutrients detected
1	Very high levels of non-nutrients detected in surrounding environment

SCORING GUIDANCE: Note: This should be scored for growout stages, not seed production.

EXAMPLES:

1. In split pond production of U.S. catfish, there are no antibiotics used in growout process. A few chemicals are used, but the chemicals have short half-lives and pond water is only discharged every 10 years. This limits the spread of the chemicals to the surrounding environment and thus this metric is scored a 5.

ENVIRONMENTAL HEALTH

WATER USE AND EFFLUENTS

Freshwater Use

RATIONALE:
Freshwater use in food production receives a lot of attention because it is projected to rapidly increase with population growth. Intensive production systems are thought to use less water than extensive production systems (Verdegem and Bosma 2009).

METRIC:
Measures the extent of consumptive freshwater use in production.

5	Virtually no consumptive use of water; 0-500L/kg; marine cages/pens and recirculating systems with treated, reusable discharge
4	Minor consumptive use of water; 501-1000L/kg; recirculating system with partial water treatment; pond farming
3	Moderate consumptive use of water; 1001-4000L/kg; many plant crops
2	High consumptive use of water; 4001-10,000L/kg; pork production
1	Very high consumptive use of water; >10,000L/kg; cattle production

SCORING GUIDANCE:
Three scales are provided: a quantitative scale when data are available and two qualitative scales – one of which characterizes production technology. This metric should account for consumptive freshwater use only – where water is consumed by evaporation or the organism, or is discharged in a non-reusable state. If water is treated and discharged in a reusable state, it should not account against the score. Note: This should be scored for growout stages, not seed production.

EXAMPLES:
1. Norwegian Atlantic salmon are farmed in marine pens where virtually no water is consumed during growout process, thus scoring a 5.

Environmental Health

Impacts To Wildlife

Wildlife Mortality

Rationale:

Attraction of wildlife to aquaculture sites can pose significant problems for farmers especially in the marine environment (Goldburg et al. 2005; Diana 2009). The provision of food and physical structure are the key factors attracting birds and marine mammals to aquaculture farms (Callier et al. 2018). In some cases, mortality of wildlife can be unintentional, such as animals getting caught in gear and netting, and in other cases, farmers intentionally kill wildlife to prevent economic loss.

Metric:

Measures the extent that farms impact regional wildlife populations. This includes intentional mortality (such as killing predators) and non-intentional mortality such as birds or other animals getting caught in gear and netting.

5	Virtually no impact on wildlife populations
4	Minimal impact on wildlife populations
3	Moderate impact on wildlife populations
2	High impact on wildlife populations
1	Severe population decline

Scoring Guidance:

This measure should not consider effects of escaped organisms on wildlife. This is captured below by the metric *Ecological impacts of escaped fish.*

Examples:

1. U.S. catfish farmers are permitted to kill a limited number of cormorants and other birds through the U.S. Fish and Wildlife Service. Given that farmers stay within the limits set by wildlife managers, the impact on bird populations is considered to be minimal so the industry scores a 4.

ENVIRONMENTAL HEALTH
IMPACTS TO WILDLIFE

Benefits to Wildlife

RATIONALE:
Negative environmental effects of aquaculture tend to receive the majority of the focus; however, aquaculture can provide environmental benefits aside from bioassimilation of nutrients. These benefits primarily occur to wildlife through increases in habitat that aquaculture gear (e.g., net pens or cages) or farmed organisms can provide. For example, farmed oysters can provide habitat for juvenile fishes and crabs. Fish farming can also directly or indirectly provide food resources for wildlife. One example is the recovery of colonial wading bird populations in Louisiana that has been linked to increased crayfish aquaculture (Fleury and Sherry 1995).

METRIC:
Measures the extent that production improve wildlife or biota. Perhaps the most common example of benefits to wildlife is the increase in habitat for fishes and crustaceans provided by oyster farming.

5	Significant improvement in biotic functions of the ecosystem
4	Improvement in biotic functions
3	Moderate improvement in biotic functions
2	Little improvement in biotic functions
1	No improvement in biotic functions

SCORING GUIDANCE:
This measure should not reflect improvements to water quality; this is captured above in the metric *Impact of discharge*.

EXAMPLES:

ENVIRONMENTAL HEALTH

IMPACTS TO WILDLIFE

Ecological Impacts of Escaped Fish

RATIONALE:

Poorly managed and unregulated aquaculture can result in the introduction of non-native species to new environments where they can negatively impact the environmental conditions through predation, competition for food and space, habitat perturbations, etc. (Naylor et al. 2001; Lin et al. 2015). Even domestic escaped fish can have substantial ecological impacts through intraspecific competition and transfer of disease and reproductive habitat perturbations as observed with Atlantic salmon (Gross et al. 1998; Fleming 1997; Ferguson 1997; Jonsson and Jonsson 2006).

METRIC:

Measures the ecological effect of escaped fish on the ecosystem. Effects can include intra- and interspecific competition of food and space, predation, and alteration of food webs. If escaped fish are non-native, the impacts are likely to be higher.

5	No observable ecological effect
4	Minor observable effects
3	Moderate ecological effects
2	Significant ecological effects
1	Major ecological impacts; sub-population or invasive species established

SCORING GUIDANCE:

EXAMPLES:

1. The U.S. catfish industry scored a 5 as there are rarely escapees. Farms are not built on major river systems and only native or hybrid species are grown.

ENVIRONMENTAL HEALTH

IMPACTS TO WILDLIFE

Genetic Impacts of Escaped Fish

RATIONALE:
Domesticated fish can be phenotypically and genetically distinct from wild populations due to the selective pressures of the rearing environment and as a result of selective breeding programs (Gross et al. 1998; Huntingford 2004; Lorenzen et al. 2012). Interbreeding and gene flow from domesticated fish to wild conspecifics can occur and lead to alteration of life history/fitness related traits such as reproductive success (Bolstad et al. 2017; Glover et al. 2017). While reproductive success of escaped fish is often low (such as with Atlantic salmon - Fleming 1996), escaped fish can still contribute substantially to the gene pool of wild populations (Gross 1997; Glover et al. 2017).

METRIC:
Measures the genetic effects of escaped fish on wild conspecifics.

5	No observable genetic effects; escapees do not breed with wild conspecifics
4	Minor genetic effects
3	Moderate genetic effects
2	Significant genetic effects
1	High levels of introgression with genetic changes in life history

SCORING GUIDANCE:
Interbreeding of domesticated fish and wild conspecifics leading to life history changes, such as reduced reproductive success, among wild fish should be scored a 1.

EXAMPLES:
1. The U.S. catfish industry scored a 5 because escapees are rare and the hybrid species are sterile.
2. Norwegian Atlantic salmon scored a 3 because escapes of farmed fish are common and there is evidence to show that farmed salmon are interbreeding with wild populations.

ENVIRONMENTAL HEALTH
IMPACTS TO WILDLIFE

Parasite and Disease Transmission

RATIONALE:
The spread of infectious pathogens from farmed to wild fish can threaten wild fish stocks and associated fisheries (Krkošek et al. 2007). Perhaps among the most contentiously debated is the transmission of sea lice from farmed to wild fish and its effect on wild salmon and trout stocks (Torrissen et al. 2013). Disease threats that reduce wild fish survival and reproductive capabilities poses threats to environmental sustainability.

METRIC:
Measures the extent of parasite and disease transmission from farms to wild fish and its effect on wild fish survival and reproduction.

5	No observable increase in parasitism or disease prevalence in wild fish
4	Slight increase in parasite or disease occurrence in wild fish with minimal effect on survival or reproduction
3	Increased parasite or disease occurrence in wild fish and/or moderate effect on survival or reproduction
2	Significant increase in parasite or disease occurrence in wild fish and/or significant effect on survival or reproduction
1	Parasite or disease transmission leading to high mortality levels and severe population decline

SCORING GUIDANCE:

EXAMPLES:
1. Atlantic salmon farms in Norway has increased the prevalence of sea lice and other parasites on wild fish and thus scores low (a score of 2) on this measure.

ENVIRONMENTAL HEALTH

IMPACTS TO WILDLIFE

Site Use

RATIONALE:
Lengthening the use of an aquaculture site is particularly important in the context of increasing the global food supply in a sustainable manner. Aquaculture sectors with rapid site turnover reflect inefficiency and unsustainable practices.

METRIC:
Measures the number of production cycles that occur on a site prior to moving the operation to a new location.

5	More than 10
4	5-10
3	4-5
2	2-3
1	1

SCORING GUIDANCE:
Normal stops in production like fallowing do not count against score.

EXAMPLES:

1. The U.S. catfish industry scores a 5 because most farms have been operational for 15-30 years and have continuously grown fish in the same ponds.

ENVIRONMENTAL HEALTH

IMPACTS TO WILDLIFE

Land Use

RATIONALE:

Alteration of land has enabled humans to expand and revolutionize food production but at the expense of the environment. Successful aquaculture industries can reconcile food production and environmental conservation objectives.

METRIC:

Measures the extent of change to land, benthic habitats, vegetation and hydrology relative to earlier use. This measures the change in land relative to use immediately prior to use for aquaculture. For example, if rainforest was historically cleared for crop farming and at a later date was modified to pond farming, the score should be based on the changes that occurred to transform it from crop farming to pond farming and should not consider clearing of the rainforest. The score can be improved if the farm conducts restoration efforts that offsets environment impacts.

5	Little to no changes to land, benthic habitat, vegetation and hydrology are made for aquaculture activities
4	Minor changes to land, benthic habitat, vegetation and hydrology, or restoration efforts on the farm or elsewhere offset environmental impact of changes
3	Moderate changes to land, benthic habitat, vegetation and hydrology, or restoration efforts partially offset environmental impact
2	Major changes to land, benthic habitat, vegetation and hydrology, or weak ability to offset environmental impact
1	Severe changes to land, benthic habitat, vegetation, hydrology, or no ability to offset environmental impact

SCORING GUIDANCE:

EXAMPLES:

In the case of Atlantic salmon farming in Norway, strategic siting of pens in areas with sufficient water exchange has prevent major environmental impacts to the benthos, so it scored a 4.

ENVIRONMENTAL HEALTH

IMPACTS TO WILDLIFE

GHG Emissions

RATIONALE:

Food production is a significant contributor to global greenhouse gas emissions (GHG). Intensive food production techniques are thought by some to produce the most food with the least amount of GHG emissions. Long-term sustainability of the food system will depend on the ability to manage and reduce GHG emissions.

METRIC:

Measure of greenhouse gas emissions attributable to production. This should be scored relative to other types of food production and measured in CO2 equivalents.

5	Virtually no GHG emissions or among the lowest in animal protein production; less than 1 kg of CO2 per kg of harvested product
4	GHG emissions are low; between 2-4 kg of CO2 per kg of product
3	GHG emissions are moderate; 4-8 kg of CO2 per kg of product
2	GHG emissions are high; 8-10 kg of CO2 per kg of product
1	GHG emissions are very high and among the highest in animal protein production; more than 10 kg of CO2 per kg of product

SCORING GUIDANCE:

This score should measure emissions from production and not from processing, transportation, etc.

EXAMPLES:

1. GHG emissions are very low in pond production of U.S. catfish with gas and diesel to conduct daily feeding and harvesting activities being a key source. Thus this industry scores a 5.

ENVIRONMENTAL HEALTH

ENVIRONMENTAL COMPLIANCE

Compliance with Environmental Law

RATIONALE:
Adherence to the law ensures that production meets basic environmental requirements.

METRIC:
Proportion of production that is compliant with environmental regulations and policies at the national, regional and local levels. The score should be discounted if environmental law is very weak or non-existent.

5 All production is compliant with environmental law
4 High compliance
3 Moderate compliance
2 Low compliance
1 Virtually no compliance with environmental law or environmental law is non-existent

SCORING GUIDANCE:

EXAMPLES:

1. The governing bodies at the local, state and federal level in the U.S. are strict and enforcement and penalties for violations are high and therefore compliance is high, so this industry scored a 5.

ENVIRONMENTAL HEALTH

CERTIFICATION

Proportion of Production with Third-party Certification

RATIONALE:
Aquaculture production must be sustainable in order to generate long-run economic profits. One goal of certification programs is to ensure that production is environmentally sustainable. Certification may also be essential for achieving market access in developed countries.

METRIC:
The proportion of production value that is certified as ecologically sustainable by a third party program such as the Aquaculture Stewardship Council (ASC) or Best Management Practices (BMP) certification.

5	76-100% of production is certified
4	51-75% of production is certified
3	26-50% of production is certified
2	1-25% of production is certified
1	No production has third party certification

SCORING GUIDANCE:
If multiple species are produced but not all have certification, weight the individual species by their farm-gate value.

EXAMPLES:

PRODUCTION SECTOR

PRODUCTION PERFORMANCE

Production Technology

RATIONALE:
Utilization of advanced farming technology is important for efficiency and wealth generation. Intensification of farms and innovations in systems design can reduce production costs and minimize threats to the environment.

METRIC:
A measure of how advanced or sophisticated the production technology is.

5	Production technology is state of the art
4	Production technology is modern
3	Production technology is functional
2	Production technology is outdated or not well developed
1	Production technology is poorly developed

SCORING GUIDANCE:
This measure should not consider how advanced the technology is for the particular species but relative to global standards across all species. For example, tilapia farming in China should not be scored relative to tilapia farming in Indonesia but relative to e.g., the most sophisticated recirculating aquaculture systems. This measure should not reflect how old or poorly maintained the technology is, rather how sophisticated or advanced the technology is for the time.

EXAMPLES:
Recirculating systems will typically be scored as 5. A normal pen based system with cameras and sensors should be scored a 4. Pond based farming with irrigation and attempts to measure density and/or feed efficiency will normally be a 3. Ponds with limited control and rope-based farming would normally score a 2. Production systems where there is no interaction with the biomass, with the possible exception of feeding, between release and harvesting should score a 1.

PRODUCTION SECTOR

PRODUCTION PERFORMANCE

Adult Feed

RATIONALE:
The field of aquatic animal nutrition is critical for sustainable aquaculture development. Growth, health and reproduction of fish are dependent on an adequate supply of quality nutrients, which requires extensive knowledge on fish nutritional requirements and consideration of the availability of high-quality feeds.

METRIC:
This measures the level of sophistication and control in feeding, particularly with respect to knowledge regarding the organism's nutritional needs and the availability and access to high-quality feeds. Culture of organisms with no control of feeding or no feed inputs, such as in some bivalve culture and in cattle grazing, should score a 1.

5	Formulated feeds match the nutritional needs of the animal and are readily available
4	Formulated feeds and extensive knowledge about nutritional needs
3	Formulated feeds and limited knowledge on nutritional needs or nutritional composition developed for other species; end feeding
2	Non-formulated feeds, household leftovers, pond fertilization
1	Forage feed, live or frozen fish or fish parts

SCORING GUIDANCE:

EXAMPLES:

PRODUCTION SECTOR

PRODUCTION PERFORMANCE

Juvenile Survival Rate

RATIONALE:

High rates of juvenile mortality reflect poor control of the life cycle, fish health and farm management practices. High mortality inhibits wealth generation and long-term success of the industry.

METRIC:

Measure of survival rate from egg through the juvenile period, or to the beginning of the grow-out period.

5	Above 65% survival
4	45-65%
3	25-45%
2	5-25%
1	Less than 5% survival

SCORING GUIDANCE:

When necessary, use the first point in the production cycle where it's possible to count/estimate organisms/survival. Intentional management-induced mortality such as selection of sex should not be counted against the score.

EXAMPLES:

PRODUCTION SECTOR

PRODUCTION PERFORMANCE

Juvenile Production

RATIONALE:
The level of control in aquaculture can vary significantly from managing a small part of the organism's life cycle to managing the entire life cycle (i.e., a closed production cycle where seed/fry are produced in a hatchery and subsequently grown out for harvest or broodstock). Greater control of juvenile production can reduce dependence on wild fisheries and reduce risk and improve wealth generation.

METRIC:
This metric measures the level of sophistication and control in juvenile production.

5	Closed production cycle
4	Closed production cycle, occasional harvest of wild fish for broodstock
3	Regular harvest of wild organisms for broodstock
2	Open production cycle, wild harvest of seed/fry
1	Wild harvest of recruited fish

SCORING GUIDANCE:
A closed production cycle that is completely independent of wild resources, e.g., Atlantic salmon, should score a 5. Sectors that have not successfully mastered juvenile rearing through the density dependent processes and are dependent on harvesting large recruited fish from the wild for growout would score a 1. Sectors that are still dependent on wild harvest for growout but can successfully grow out wild eggs or fry should score a 2.

EXAMPLES:
1. Atlantic salmon production in Norway is completely independent of wild resources. When more genetic diversity is needed, hatcheries trade broodstock amongst one another rather than harvesting from the wild. Thus, scoring a 5.

PRODUCTION SECTOR

PRODUCTION PERFORMANCE

Selective Breeding and Production Time

RATIONALE:
Farming of genetically improved aquaculture species is important for the development of profitable aquaculture industries that can better utilize feed, space and water resources. In addition, there are potential ecological benefits of breeding programs such as reduced dependence on wild stocks.

METRIC:
Measures the extent that the production cycle has been shortened as a result of selective breeding.

5	Production time has been reduced by more than 50% and selection for traits other than growth
4	Production time has been reduced by more than 50%
3	Production time has been reduced by more than 20%
2	Production time has been reduced by more than 10%
1	No improvement in production time

SCORING GUIDANCE:

EXAMPLES:

1. Hybrid catfish can now be harvested in less than 16 months compared to the previous two-year crop cycle. Thus, scoring a 3.

PRODUCTION SECTOR

PRODUCTION PERFORMANCE

Survival Trend

RATIONALE:
High rates of adult mortality reflect poor control of production, fish health and farm management practices. High mortality inhibits wealth generation and long-term success of the industry.

METRIC:
Ratio of the current survival rate to the three highest survival rates in the past.

5	Survival rate is at least 95% of historic high
4	Survival rate is 85-95% of historic high
3	Survival rate is 70-85% of historic high
2	Survival rate is at least 50-70% of historic high
1	Survival rate is below 50% of historic high

SCORING GUIDANCE:
The survival rate of the adult grow-out stage (post-recruitment) should be considered in this metric. The fry and juvenile stages where density-dependent processes strongly drive mortality is considered in the *Juvenile Survival Rate* above.

EXAMPLES:

PRODUCTION SECTOR

PRODUCTION PERFORMANCE

Survival Rate

RATIONALE:
High rates of adult mortality reflect poor control of production, fish health and farm management practices. High mortality inhibits wealth generation and long-term success of the industry.

METRIC:
Measure of survival rate during the adult growout period.

5	Above 95% survival
4	85-95%
3	70-85%
2	50-70%
1	Less than 50% survival

SCORING GUIDANCE:
Intentional management-induced mortality such as selection of sex should not be counted against the score.

EXAMPLES:

1. The survival rate of hybrid catfish in U.S. growout ponds is between 85 and 90%, so the industry scores a 4.

PRODUCTION SECTOR
PRODUCTION PERFORMANCE

Proportion of Production Affected Disease and Parasites

RATIONALE:
Losses in production can be significant threats to economic stability and wealth generation. An aquaculture sector can fail to generate wealth if threaten by disease and if the proper institutions are not in place to prevent, control and treat outbreaks.

METRIC:
The extent that production value is affected by disease and parasites.

5	Production value unaffected by disease
4	Production value reduced by less than 10%
3	Production value reduced by 10-30%
2	Production value reduced by more than 30%
1	Production value almost completely eliminated by disease

SCORING GUIDANCE:
This should consider production value lost through mortality, reduced growth and the costs of treatment. It should not consider prevention expenses or fluctuations in market price due to disease in neighboring farms/industries.

EXAMPLES:
1. In split pond production of hybrid catfish intensive aeration and improved production practices has lowered mortalities due to disease to less than 10% so the industry scores a 4.

PRODUCTION SECTOR
PRODUCTION PERFORMANCE

Proportion of Production Affected by Predation

RATIONALE:
Losses in production can be a significant threat to economic stability and wealth generation. One potential source of fish mortality is predation.

METRIC:
The extent that production value is affected by predation.

5	Production value unaffected by predation
4	Production value reduced by less than 10%
3	Production value reduced by 10-30%
2	Production value reduced by more than 30%
1	Production value almost completely eliminated by predation

SCORING GUIDANCE:
This measure should not consider costs to prevent predation and should not consider human predation or theft.

EXAMPLES:
1. There are not good estimates of predation in catfish ponds. There are avian predators but it is likely less to contribute to less than 10% so the industry scores a 4.

PRODUCTION SECTOR

PRODUCTION PERFORMANCE

Proportion of Production that Escapes

RATIONALE:

Losses in production can be a significant threat to economic stability and wealth generation. Fish escapes are one potential source of production loss. High levels of escaped fish reflect inadequate facility and containment operations, wasted profits and economic risk.

METRIC:

The extent that production value is affected by escape events.

5	Production value basically unaffected by escapes
4	Production value reduced by less than 10%
3	Production value reduced by 10-30%
2	Production value reduced by more than 30%
1	Production value almost completely eliminated by escapes

SCORING GUIDANCE:

Regulatory fines for escapees should not be considered here.

EXAMPLES:

1. In the U.S. catfish industry, fish cannot escape from the split ponds that are used for growout, so the industry scores a 5.

PRODUCTION SECTOR

PRODUCTION PERFORMANCE

Proportion of Production Affected by Handling and Unspecified Loss

RATIONALE:
Losses in production can be a significant threat to economic stability and wealth generation. Fish lost to handling, unspecified loss and theft may reflect poor practices, weak property rights, wasted profits and economic risk that can deter investment.

METRIC:
The extent that production value is affected by handling, unspecified losses, and theft.

5 Production value unaffected by handling and unspecified losses
4 Production value reduced by less than 10%
3 Production value reduced by 10-30%
2 Production value reduced by more than 30%
1 Production value almost completely eliminated by handling and unspecified losses

SCORING GUIDANCE:
This measure should only consider production value lost in the production/harvest sector; losses in the processing sector are captured below in the metric *Shrink*.

EXAMPLES:
1. Handling and harvesting practices are well established in the U.S. catfish industry and losses due to these practices are trivial. There is no loss due to theft and other unspecified losses, so the industry scores a 5.

PRODUCTION SECTOR

PRODUCTION PERFORMANCE

Production Costs Compared to Historic High

RATIONALE:
An aquaculture sector that is generating wealth will reinvest in capital and research and orient towards market access, innovation and efficiency. This should be observable in declining production costs.

METRIC:
Ratio of current production costs to the highest three production costs in the last 10 years. If data is available this should be computed in real costs (i.e., adjust by local CPI if inflation was significant).

5 Less than 50% of historic highs
4 Less than 80%
3 Less than 120%
2 Less than 150%
1 More than 150% of historic highs

SCORING GUIDANCE:
This metric is meant to measure the extent that production costs have declined. Preferably, data should be aggregated at the industry-level (not for individual farms). See the excel worksheet labeled "Historical Data" for the calculations. If data are not available, try to get industry participants to estimate general trends about whether production costs have been rising or falling and by how much.

EXAMPLES:
The cost of current production of U.S. hybrid catfish in split ponds is between $0.79 - $0.92/lb. The cost in traditional pond production was between $1.02 - $1.10/lb

PRODUCTION SECTOR
PRODUCTION ASSETS

Ratio of Asset Value to Gross Earnings

RATIONALE:
In addition to income, wealth can accumulate to producers through the value of assets that allow access and participation in the industry. The price of farming capital, in the form of technology, infrastructure, land or water permits, is a direct measure of the accumulation of wealth in the industry. The price of access should reflect the present discounted value of the profits arising from access to production. This will include expectations for changes in management, production levels, prices and costs. Gross earnings are used to normalize the asset value to the level of production. Gross earnings are a proxy for net earnings because cost data are rarely available, and this normalization is standard in agricultural frameworks. For a fixed level of gross earnings, if the sector's income is highly uncertain or costs are excessive then the ratio would be lower.

METRIC:
Extent to which wealth is accumulated in farming assets (e.g., land value, land or water permits, farm capital, production technology and infrastructure, etc.). Ratio of average annual price of capital and licenses required to participate in the industry over the past 5 years to the average annual gross earnings in the same period for a similarly scaled access right in the same period.

5	10 or higher
4	7.5-10
3	5-7.5
2	2.5-5
1	2.5 or below

SCORING GUIDANCE:
Calculation: Annual cost of access /Annual gross earnings per access capital (average for last five years)

When determining the price of capital, try to capture at least 80% of the asset value required for access; for example, if participation requires a permit, vessel, and marine cages, but data on vessel prices are hard to obtain, just focus on the permit and marine cages. Use data on purchase value and not lease value of assets. The price of access should represent the cost to a new entrant and not the present value of existing farmers' assets. See the excel worksheet labeled "Historical Data" for the calculations. If data are not available, try to get industry participants to estimate general trends about whether asset values and earning have been rising or falling and by how much.

EXAMPLES:

PRODUCTION SECTOR

PRODUCTION ASSETS

Total Revenue Compared to Historic High

RATIONALE:

If the production sector is generating wealth, it is expected that the total revenue for the fishery is likely to increase to some sustainable maximum range. Production sectors with declining total revenue are likely to be in decline due to poor management, poor marketing, or disease. In contrast, a sector managed for wealth creation should be producing seafood sustainably and will likely to orient towards market access and innovation. This should be observable in stable or increasing total revenue.

METRIC:

Ratio of total real revenue (in local currency) to the average of the three highest total real revenues in the past 10 years. Adjust by local CPI if inflation was significant.

5	Above 95%
4	85 to 95%
3	70 to 85%
2	50 to 70%
1	Below 50%

SCORING GUIDANCE:

This metric is meant to measure the extent that total revenues are stable or increasing. See the excel worksheet labeled "Historical Data" for the calculations. If data are not available, try to get industry participants to estimate general trends about whether revenue has been rising or falling and by how much.

EXAMPLES:

PRODUCTION SECTOR

PRODUCTION ASSETS

Asset Value Compared to Historic High

RATIONALE:
If the sector is generating wealth, it is expected that the value of physical capital and permits will increase to some sustainable maximum range. Production sectors with declining assets are likely to be in decline as a result of poor management, poor marketing, or disease. In contrast, a sector managed for wealth creation should be producing seafood sustainably and will likely orient to improved marketing and innovation.

METRIC:
Ratio of the current value of the asset (e.g., land or water permits, production technology and infrastructure) to the average of the three highest asset values in the past 10 years. Adjust by local CPI if inflation was significant.

5	Above 95 percent
4	85 to 95 percent
3	70 to 85 percent
2	50 to 70 percent
1	Below 50 percent

SCORING GUIDANCE:
When determining the price of capital, try to capture at least 80% of the asset value required for access; for example, if participation requires a permit, vessel, and marine cages, but data on vessel prices are hard to obtain, just focus on the permit and marine cages. Use data on purchase value and not lease value of assets. The price of access should represent the cost to a new entrant and not the present value of existing farmers' assets. See the excel worksheet labeled "Historical Data" for the calculations. If data are not available, try to get industry participants to estimate general trends about whether asset values have been rising or falling and by how much.

EXAMPLES:

PRODUCTION SECTOR

PRODUCTION ASSETS

Borrowing Rate Compared to Risk-Free Rate

RATIONALE:

The premium demanded by the capital market to make loans is a direct measure of financial risk in the sector. It is locally normalized to reflect the overall riskiness in the region and opportunities available to local capital.

METRIC:

Average ratio between the interest rate on loans made to producers in the industry to risk-free rates over the last three years.

5	Ratio less than 1.75; cf. 30-year conforming mortgage
4	Ratio less than 2.5; cf. personal bank loan
3	Ratio less than 4; cf. good credit card rates
2	Ratio less than 7; cf. bad credit card rates
1	Ratio greater than 7; usury

SCORING GUIDANCE:

Calculation: Interest rate in the producing industry / Risk-free interest rate (average over last 3 years).

Note that if harvesting businesses can access international credit markets, then the international risk-free rate (US 10-year Treasury bill) is an appropriate comparison, but if businesses are forced to use local credit markets then the benchmark should be local risk-free rates. Typically national/municipal government bonds will be the best representative of local risk-free rates. When scoring, it is often easier to ask the next question about the source of capital funds first and then ask about the rates that they pay. As long as there are credit transactions this metric should not be NA; strive to get some estimate of the interest rates that producers pay.

In some sectors, there are cultural or religious prohibitions on interest-based lending. If capital is paid out of cash flow, this can be NA. If proxy metrics are used to capture time value of capital, develop a best guess for the metric.

EXAMPLES:

PRODUCTION SECTOR

PRODUCTION ASSETS

Source of Capital

RATIONALE:

The availability of lending capital from particular sources is a direct measure of how the capital market assesses risk in the production sector. If a certain type of lender or investor is not willing to make capital available in the production sector at any price, it reveals that it is much riskier than other available investments.

METRIC:

A measure of the category of lenders or investors that are most typically used by producers in the sector. Second scoring method offered (after the semi-colon) if the supply chain (e.g., traders, processors, exporters) are the primary source of capital.

5	Unsecured business loans from banks/public stock offering;
4	Secured business loans from banks/venture capital; investment from elsewhere in the supply chain
3	Loans from banks secured by personal (not business) assets/Government subsidized private lending/Government-run loan programs/International aid agencies; secured loans from elsewhere in supply chain
2	Microlending/Family/Community-based lending/ Producer association lending group; loans from supply chain that significantly reduce margins
1	Mafia/No capital available; exploitative relationship from elsewhere in supply chain

SCORING GUIDANCE:

Please note in the worksheet explanation which scoring method was used (i.e. whether or not the supply chain is the primary source of capital). In the context of contract farming, inputs provided by a contractor would count towards this measure. Secured contractor inputs should be scored a 3 and unsecured inputs should score a 4.

EXAMPLES:

1. The source of capital in the U.S. catfish industry is diverse, but many farmers get capital through business loans at local banks and thus score a 4.

PRODUCTION SECTOR

PRODUCTION ASSETS

Functionality of Production Capital

RATIONALE:

The age of the facilities on farms used for producing fish, primarily hatcheries, growout facilities, and storage facilities, reflects several dimensions of sector wealth. First, it is a direct measure of wealth that has accumulated from the sector and reinvested in capital. Second, it is a measure of the potential wealth of the sector, as newer facilities will be more efficient and less costly to operate. Third, if farmers are willing to invest in new capital, it reflects an assessment that the sector will be profitable in the future.

METRIC:

Average age of the key durable producer capital unit (buildings, equipment, vessels). Ages are not assigned to scores due to differences in expected useful life, but buildings and industrial vessels have expected life of roughly 20 years.

5	Capital is new or up to date
4	Capital is older but well maintained, e.g., freshly painted
3	Capital is moderately well maintained
2	Maintenance is poor
1	Serious concerns about functionality and safety

SCORING GUIDANCE:

EXAMPLES:

1. The equipment and technology (e.g., the water movement systems) used in the U.S. catfish split pond production is fairly new (less than 8 years old) and most kept up to date and in excellent condition, so the industry scores a 5.

PRODUCTION SECTOR

RISK

Annual Total Revenue Volatility

RATIONALE:

Annual total production volatility is primarily a measure of riskiness of production. When future production levels are variable, it is difficult to make investment decisions and secure capital because future income streams are highly uncertain. High production volatility also presents an obstacle to developing final product markets, as large processors, exporters and retailers prefer to deal with products for which they can develop long-term contracts.

METRIC:

Ratio of the standard deviation of the first differences of annual total revenue to the mean of total revenue over the last 10 years. Best guess may be calculated based on shorter time series if data are not available.

5	Less than 0.15
4	0.15-0.22
3	0.22-0.40
2	0.40-1
1	Greater than 1

SCORING GUIDANCE:

See the excel worksheet labeled "Volatility" for the calculations. Make note of the years used for the calculation in the worksheet and make sure to assess whether the data from a shorter time-series is reasonable/representative. Note that this metric is de-trended, so scores cannot be determined by simply looking at trends.

EXAMPLES:

PRODUCTION SECTOR

RISK

Annual Production Volatility

RATIONALE:

Annual total production volatility is primarily a measure of riskiness of production. When future production levels are variable, it is difficult to make investment decisions and secure capital because future income streams are highly uncertain. High production volatility also presents an obstacle to developing final product markets, as large processors, exporters and retailers prefer to deal with products for which they can develop long-term contracts.

METRIC:

Ratio of the standard deviation of the first differences of annual total production to the mean of total production over the last 10 years. Best guess may be calculated based on shorter time series if data not available.

5	Less than 0.15
4	0.15-0.22
3	0.22-0.40
2	0.40-1
1	Greater than 1

SCORING GUIDANCE:

See the excel worksheet labeled "Volatility" for the calculations. Make note of the years used for the calculation in the worksheet and make sure to assess whether the data from a shorter time-series is reasonable/representative. Note that this metric is de-trended, so scores cannot be determined by simply looking at trends.

If precise historical data on production is not available, ask for an estimate of whether it was higher or lower last year, then ask if they were 10% different, 20% different, etc. This should be able to be completed for at least the past 3 years.

EXAMPLES:

PRODUCTION SECTOR

RISK

Intra-annual Production Volatility

RATIONALE:
High frequency (weekly or monthly) volatility in price is primarily a measure of the potential for wealth generation. High volatility may reflect inefficient regional regulation of production or inefficient farm-level management. Spikes in production can hinder wealth creation through several ways. First, concentrating harvest in a short period spikes supply and often suppresses price. Second, processing capacity must be established to handle spikes in supply and if it is not, product will be underutilized and costly per unit processed. Spikes in processing volume can compromise the yield and quality of the processed product. Finally, intra-annual volatility can make it difficult for processors to make forward contracts for their products and thus receive lower prices.

METRIC:
Ratio of the standard deviation of the weekly/monthly total production over the last three years to the mean of total weekly/monthly production. Best guess may be calculated based on shorter time series if data not available (e.g., if detailed data is only available for the past year).

5	Less than 0.15
4	0.15-0.22
3	0.22-0.40
2	0.40-1
1	Greater than 1

SCORING GUIDANCE:
See the excel worksheet labeled "Volatility" for the calculations. Make note of the years used for the calculation in the worksheet and make sure to assess whether the data from a shorter time-series is reasonable/representative. If precise historical data on production is not available, ask for an estimate of the percentage that production fluctuates each month. Observations of zeros (i.e., no production) are included.

EXAMPLES:

PRODUCTION SECTOR
RISK

Annual Price Volatility

RATIONALE:
Annual price volatility complements annual production volatility to capture the wealth generation potential in the sector. When future revenues are variable, it is difficult to make investment decisions and secure capital because future income streams are highly uncertain. High price volatility may reflect obstacles to developing final product markets, as large processors and exporters prefer to deal with products for which they can develop long-term contracts.

METRIC:
Ratio of the standard deviation of the first differences of annual farm-gate prices to the mean of farm-gate price over the last 10 years. Best guess may be calculated based on shorter time series if data not available.

5	Less than 0.13
4	0.13-0.20
3	0.20-0.30
2	0.30-0.85
1	Greater than 0.85

SCORING GUIDANCE:
See the excel worksheet labeled "Volatility" for the calculations. Make note of the years used for the calculation in the worksheet and make sure to assess whether the data from a shorter time-series is reasonable/representative. Note that this metric is de-trended so scores cannot be determined by simply looking at trends.

Price data may not be available for vertically integrated industries where price is not determined for transfers within a company. If farm-gate prices are not available, then wholesale prices should be used. If precise historical data on prices are not available, ask for an estimate of whether it was higher or lower last year, then ask if they were 10% different, 20% different, etc. This should be able to be completed for at least the past 3 years.

EXAMPLES:

PRODUCTION SECTOR

RISK

Intra-annual Price Volatility

RATIONALE:

Intra-annual price volatility complements intra-annual production volatility to capture the wealth generation potential in the sector. Price changes arise from shifts in demand or changes in supply. If price volatility is high, unconstrained producers could shift harvests from periods of low price to a period of higher price and increase profits.

METRIC:

Ratio of the standard deviation of average monthly farm-gate prices over the last three years to the mean farm-gate price. Best guess may be calculated based on shorter time series if data not available.

5	Less than 0.13
4	0.13-0.20
3	0.20-0.30
2	0.30-0.85
1	Greater than 0.85

SCORING GUIDANCE:

See the excel worksheet labeled "Volatility" for the calculations. Make note of the years used for the calculation in the worksheet and make sure to assess whether the data from a shorter time-series is reasonable/representative. If precise historical data on prices are not available, ask for an estimate of the percentage that prices fluctuates each month. Price data may not be available for vertically integrated fisheries where price is not determined for transfers within a company. If farm-gate prices are not available, then wholesale prices should be used. This metric aims to capture the extent to which prices vary over an entire season, so daily/weekly observations can be averaged to larger periods so that there are 10-20 observations during each season.

EXAMPLES:

PRODUCTION SECTOR

RISK

Spatial Price Volatility

RATIONALE:

The extent to which farm-gate price for the same product varies across different farms within the region reflects market integration. A market that is well integrated spatially will have similar prices, whereas isolated production or production that varies in how well they are connected to markets, thus posing greater financial risk, will have higher levels of spatial volatility.

METRIC:

Ratio of the standard deviation across data collection regions of average annual farm-gate price to the mean farm-gate price across data collection regions. Measure should be averaged over last three years. Best guess may be calculated based on shorter time series if data not available.

5	Less than 0.13
4	0.13-0.20
3	0.20-0.30
2	0.30-0.85
1	Greater than 0.85

SCORING GUIDANCE:

See the excel worksheet labeled "Volatility" for the calculations. Make note of the years used for the calculation in the worksheet and make sure to assess whether the data from a shorter time-series is reasonable/representative. If precise historical data on prices are not available, ask for an estimate of the percentage that prices fluctuates by region. Price data may not be available for vertically integrated fisheries where price is not determined for transfers within a company. If farm-gate prices are not available, then wholesale prices should be used.

EXAMPLES:

PRODUCTION SECTOR
RISK

Contestability and Legal Challenges

RATIONALE:
Legal challenges, protests and contentious public hearings reflect discontent with the management system. It is an indicator of a lack of social acceptance and a source of considerable risk. This diminishes the welfare that is accruing to the community if the sector is a source of discord.

METRIC:
This captures the degree to which political activity limits the ability to implement effective management regulations. Fish farming in some areas is prone to contestability due to cultural norms and institutions, while other discontent develops as a response to ineffective management.

5	No significant legal challenges, civil actions, or protests regarding the management system
4	Minor legal challenges slow implementation
3	Legal challenges, civil actions, or protests impede some management measures
2	Legal challenges, civil actions, or protests suspend major elements of the management system
1	Legal challenges, civil actions, or protests suspend or prohibit implementation of key management reforms and regulation

SCORING GUIDANCE:

EXAMPLES:

PRODUCTION SECTOR

FARM OWNERS

Earnings Compared to Regional Average Earnings

RATIONALE:
Farm owner earnings are a direct measure of the type of workers that are attracted to the industry. Earnings are normalized by average regional earnings to reflect whether the sector is able to attract the most talented workers. It also reflects how well the industry is generating wealth relative to the local standards.

METRIC:
Ratio of annual earnings per farm owner to the average earnings in the region. The owners are defined as those holding the ability to access, including rights and capital. Note that this is earnings from all sources, not just farming and is compared to the regional/national levels where the farmer conducts the majority of their economic activity.

5	More than 50% above the regional average
4	Between 10 and 50% above regional average
3	Within 10% of the regional average
2	Between 50% and 90% of the regional average
1	Less than half of the regional average

SCORING GUIDANCE:
Make sure that this variable and the following metrics in the owner category are scored for the people who hold the ability to access. In many cases, farmers hold the ability to access and own the capital, but in some cases, farms are owned by companies who contract temporary farmers in which case the farmers would be considered farm workers. This metric is meant to measure what type of people the sector attracts; thus we consider all income for an entire year from any sources. These earnings should be compared to regional/national levels depending on the economic sphere of the owners. Economic sphere is defined as the region where the owners conduct the majority of their economic activity, i.e. the village if all economic activity is within the village, but the nation if the owner participates in national markets as a consumer.

EXAMPLES:

PRODUCTION SECTOR
FARM OWNERS

Owner Wages Compared to Non-Aquaculture Wages

RATIONALE:
Farm owner wages are a direct measure of wealth that accumulates to owners. Wages are normalized by wages typical of alternate jobs within the region to provide an indicator of the relative standard of living afforded to workers. It also reflects whether the industry is attracting the most skilled farmers.

METRIC:
Ratio of the owner's average daily wage in this industry to the average daily wage of the owner's alternative occupation that is within their economic sphere (e.g., jobs in the village that the owner qualifies for if all economic activity is within the village, but if labor markets are fluid then this should be national average wage for jobs that the owner expects to be able to obtain).

5	More than 50% above the alternative wage
4	Between 10 and 50% above alternative wage
3	Within 10% of the alternative wage
2	Between 50% and 90% of the alternative wage
1	Less than half the alternative wage

SCORING GUIDANCE:
This is meant to measure the average personal opportunity cost of participating in the sector; thus the alternative wage should be the answer to the question "If you couldn't farm fish, how much would you get paid?" Look at the average daily wage for farm owners when participating in the sector and then compare it to the wage in their next best alternative. If the owners think that without the aquaculture sector they would be construction workers, then score based on construction worker wages, but if they think that they would be subsistence farming, then compare it to that wage. Again, this should all be relative to wages within their economic sphere so consider national wages if labor markets are fluid, but restrict the comparison to wages within the village/region if owners would not leave their local community to find work.

EXAMPLES:

PRODUCTION SECTOR

FARM OWNERS

Education Access

RATIONALE:

A community that is successfully using its resources and extracting wealth from its industries will be able to provide high levels of education to its children.

METRIC:

This metric is based on the highest level of education that is politically and financially accessible (available and affordable) to families of farm owners. This is not based on the actual attainment levels of farm owners and families.

5	Higher education is accessible
4	High school level education or advanced technical training is accessible
3	Middle school level education or simple technical training is accessible
2	Basic literacy and arithmetic training is accessible
1	Formal education is not accessible

SCORING GUIDANCE:

This metric is meant to capture whether farm owners can afford to provide education for their children. Just because there is a high school in the village does not mean that farmers can afford to send their children – consider school fees, tuition, and opportunity cost. Note that learning to farm fish as an on the job apprenticeship does not count as formal technical training. Advanced technical training involves science/technology and most apprenticeships in LDCs do not count and should be classified as simple technical training at the most.

EXAMPLES:

Production Sector
Farm Owners

Access to Health Care

Rationale:
A community that is successful in extracting wealth from the industry will be able to provide high levels of healthcare, ensuring quality of life and reducing health risk.

Metric:
This metric is based on the quality of health care that is politically and financially accessible (available and affordable) to farm owners and their families.

5	Global standard treatment for illness is accessible
4	Licensed doctors provide trauma, surgical and drug treatments
3	Nurses or medical practitioners provide emergency and routine drug treatments
2	Basic and simple drug treatment is accessible
1	Medical or drug treatment is not accessible

Scoring Guidance:
This metric is meant to capture whether farm owners can afford to provide health care for themselves and their families. Just because there is a clinic in the village does not mean that farmers can afford it – consider medical fees, travel time and opportunity cost. The WHO's health service coverage index is used as a basis for identifying whether local care facilities are capable of providing global standard care (http://apps.who.int/gho/portal/uhc-cabinet-wrapper-v2.jsp?id=1010501). If there is a global-standard hospital located in a major city a day's journey away, then global standard treatment for illness is not fully accessible because if there was a major trauma then the injured party would likely die before reaching medical assistance (unless there is a Coast Guard helicopter assigned to transport injured workers/family members). Score based on the health facilities that are used most frequently for routine procedures and somewhat urgent issues. Sectors that have established protocol to care for farmers in the event of emergencies should score slightly higher than those where there are no such measures in place.

Examples:

PRODUCTION SECTOR

FARM OWNERS

Social Standing of Farm Owners

RATIONALE:
This is a proxy for income associated with owning a fish farm, which may be easier to collect or more accurate than actual wage information. Social standing reflects whether the sector is able to attract the most talented workers in the community and signals the extent of wealth generation relative to the local standards.

METRIC:
This metric is based on the social standing of farmer owners within the community where they spend the majority of their time.

5	Among the most respected in the community, comparable with civic and religious leaders and professionals, such as doctors and lawyers
4	Comparable to management and white collar jobs
3	Comparable to skilled labor jobs
2	Comparable to unskilled blue collar or service jobs
1	Among the least respected, such as slaves or indentured servants

SCORING GUIDANCE:
This is meant to reflect the amount of esteem that farmers receive in their local community. Make sure that answers for this metric make sense relative to the social status of the other related occupations (farmer workers, processing managers, and processing workers). Consider their social status within their primary community. The comparison group should be in the region where farmers spend the majority of their time and income.

EXAMPLES:
1. In rural areas where U.S. catfish farming is conducted, farms heavily support local employment opportunities and the local communities and as such farm owners are very well respected (score of 5).

PRODUCTION SECTOR
FARM OWNERS

Proportion of Nonresident Owners

RATIONALE:
Benefits to the community or region relies on the ability to maintain local multipliers by keeping wealth in the region. Non-resident capital reflects that wealth will be leaving the region and failing to boost the regional economy. In developing economies, it may also reflect an inability of locals to generate sufficient capital.

METRIC:
Proportion of farm owners who are local. "Local" is defined as coming from, and spending their earnings within, the local community. Nationals who are transient nonresidents, or considered outsiders in the community, are not local.

5	95-100% local
4	70-95% local
3	35-70% local
2	5-35% local
1	Virtually no local owners

SCORING GUIDANCE:

EXAMPLES:
1. In the U.S. catfish industry, farm owners live and spend their earnings in the local communities, and so it is scored a 5.

PRODUCTION SECTOR
FARM WORKERS

Earnings Compared to Regional Average Earnings

RATIONALE:
Farm worker earnings are a direct measure of the type of workers that are attracted to the industry. Earnings are normalized by average regional earnings to reflect whether the sector is able to attract the most talented workers. It also reflects how well the industry is generating wealth relative to the local standards.

METRIC:
Ratio of annual earnings per farm worker to the average earnings in the region. Farm workers are defined as those depending on others for access. Note that this is earnings from all sources, not just farming and is compared to the regional/national levels where the farm worker conducts the majority of their economic activity.

5	More than 50% above the regional average
4	Between 10 and 50% above regional average
3	Within 10% of the regional average
2	Between 50% and 90% of the regional average
1	Less than half of the regional average

SCORING GUIDANCE:
Make sure that this variable and the following metrics in the farm worker category are scored for the people who depend on others for the ability to access; this would include moderately skilled farmers if the farms are owned by companies who contract temporary farmers. Make sure that the answers to the farm worker metrics make sense relative to the farm owners.

This metric is meant to measure what type of people the sector attracts; thus we consider all income for an entire year from any sources. These earnings should be compared to regional/national levels depending on the economic sphere of the workers. Economic sphere is defined as the region where the workers conduct the majority of their economic activity, i.e. the village if all economic activity is within the village, but the nation if the worker participates in national markets as a consumer.

EXAMPLES:

PRODUCTION SECTOR

FARM WORKERS

Worker Wages Compared to Non-Aquaculture Wages

RATIONALE:

Farm worker wages are a direct measure of wealth that accumulates to workers. Wages are normalized by wages typical of alternate jobs within the region to provide an indicator of the relative standard of living afforded to workers. It also reflects whether the industry is attracting the most skilled farm workers.

METRIC:

Ratio of the farm worker's average daily wage in this industry to the average daily wage of the worker's alternative occupation that is within their economic sphere. If all economic activity is within the village, the comparison is to jobs in the village that the worker qualifies for, but if the worker participates in national markets as a consumer and labor markets are fluid, then the comparison is with jobs within the nation that the worker qualifies for.

5	More than 50% above the alternative wage
4	Between 10 and 50% above alternative wage
3	Within 10% of the alternative wage
2	Between 50% and 90% of the alternative wage
1	Less than half the alternative wage

SCORING GUIDANCE:

This is meant to measure the average personal opportunity cost of participating in the sector; thus the alternative wage should be the answer to the question "If you couldn't work on the farm, how much would you get paid?" Look at the average daily wage for farm workers when participating in the sector and then compare it to the wage in their next best alternative. If the workers think that without the aquaculture sector they would be construction workers, then score based on construction worker wages, but if they think that they would be subsistence farming, then compare it to that wage. Again, this should all be relative to wages within their economic sphere so consider national wages if labor markets are fluid, but restrict the comparison to wages within the village/region if workers would not leave their local community to find work.

EXAMPLES:

PRODUCTION SECTOR

FARM WORKERS

Education Access

RATIONALE:

A community that is successful in extracting wealth from the industry will be able to provide high levels of education.

METRIC:

This metric is based on the highest level of education that is politically and financially accessible (available and affordable) to families of farm workers. This is not based on the actual attainment levels of farm workers and families.

5	Higher education is accessible
4	High school level education or advanced technical training is accessible
3	Middle school level education or simple technical training is accessible
2	Basic literacy and arithmetic training is accessible
1	Formal education is not accessible

SCORING GUIDANCE:

This metric is meant to capture whether farm workers can afford to provide education for their children. Just because there is a high school in the village does not mean that workers can afford to send their children – consider school fees, tuition, and opportunity cost. Note that learning to farm fish as an on the job apprenticeship does not count as formal technical training. Advanced technical training involves science/technology and most apprenticeships in LDCs do not count and should be classified as simple technical training at the most.

EXAMPLES:

PRODUCTION SECTOR
FARM WORKERS

Access to Health Care

RATIONALE:
A community that is successful in extracting wealth from the industry will be able to provide high levels of healthcare, ensuring quality of life and reducing health risk.

METRIC:
This metric is based on the quality of health care that is politically and financially accessible (available and affordable) to farm workers and their families.

5	Global standard treatment for illness is accessible
4	Licensed doctors provide trauma, surgical and drug treatments
3	Nurses or medical practitioners provide emergency and routine drug treatments
2	Basic and simple drug treatment is accessible
1	Medical or drug treatment is not accessible

SCORING GUIDANCE:
This metric is meant to capture whether farm workers can afford to provide health care for themselves and their families. Just because there is a clinic in the village does not mean that workers can afford it – consider medical fees, travel time and opportunity cost. The WHO's health service coverage index is used as a basis for identifying whether local care facilities are capable of providing global standard care (http://apps.who.int/gho/portal/uhc-cabinet-wrapper-v2.jsp?id=1010501). If there is a global-standard hospital located in a major city a day's journey away, then global standard treatment for illness is not fully accessible because if there was a major trauma then the injured party would likely die before reaching medical assistance (unless there is a Coast Guard helicopter assigned to transport injured workers/family members). Score based on the health facilities that are used most frequently for routine procedures and somewhat urgent issues. Sectors that have established protocol to care for farmers in the event of emergencies should score slightly higher than those where there are no such measures in place.

EXAMPLES:

PRODUCTION SECTOR
FARM WORKERS

Social Standing of Farm Workers

RATIONALE:
This is a proxy for income associated with working on a farm, which may be easier to collect or more accurate than actual wage information. Social standing reflects whether the sector is able to attract the most talented workers in the community and signals the extent of wealth generation relative to the local standards.

METRIC:
Measure is based on the social standing of farm workers within the community where they spend the majority of their time and income.

5	Among the most respected in the community, comparable with civic and religious leaders and professionals, such as doctors and lawyers
4	Comparable to management and white collar jobs
3	Comparable to skilled labor jobs
2	Comparable to unskilled blue collar or service jobs
1	Among the least respected, such as slaves or indentured servants

SCORING GUIDANCE:
This is meant to reflect the amount of esteem that farm workers receive in their local community. Make sure that answers for this metric make sense relative to the social status of the other related occupations (farm owners, processing managers, and processing workers). Consider their social status within their primary community. The comparison group should be in the region where farm workers spend the majority of their time and income.

EXAMPLES:
1. Many workers operating split pond catfish systems are skilled workers. The equipment and technology is more advanced that traditional pond systems and requires more skilled labor, so the industry scores a 3 on this metric.

PRODUCTION SECTOR

FARM WORKERS

Proportion of Nonresident Workers

RATIONALE:

Benefits to the community or region relies on the ability to maintain local multipliers by keeping wealth in the region. A large portion of non-resident employment reflects that wealth will be leaving the region and failing to boost the regional economy. In developing economies, it may also reflect an inability of locals to generate sufficient capital.

METRIC:

Proportion of farm workers who are local. "Local" is defined as coming from, and spending their earnings within, the local community. Nationals who are transient nonresidents, or considered outsiders in the community, are not local.

5	95-100% local
4	70-95% local
3	35-70% local
2	5-35% local
1	Virtually no local workers

SCORING GUIDANCE:

EXAMPLES:

PRODUCTION SECTOR

FARM WORKERS

Worker Experience

RATIONALE:

The rate at which workers turn over is an indirect measure of several key variables. First, it reflects wealth accumulation to workers because a worker will only stay if the wage is comparable to, or better than, other obtainable jobs. Second, worker longevity often means they are residents of the community and thus their earnings stay in the community and are spent locally rather than being sent away by immigrant workers. Third, experienced workers developed specialized knowledge and skills that make farming more efficient, so the sector is better able to reach its wealth generating potential.

METRIC:

Average years of experience of farm workers.

5	More than 10 years (skilled career worker)
4	5-10 years
3	3-5 years
2	1-3 years
1	0 full years of experience (mostly new workers each season)

EXAMPLES:

PRODUCTION SECTOR
FARM WORKERS

Age Structure of Workers

RATIONALE:
A widely distributed age structure is an indirect measure of several important variables. Broadly, it reflects both that experienced, older farm workers are willing to stay in the sector and that new workers are willing to enter the sector and that jobs are available. It reflects wealth accumulation to workers because an experienced worker will only stay, and new workers will only enter, if wages are comparable to or better than other obtainable jobs. Worker longevity often means they are residents of the community and thus their earnings stay in the community and are spent locally rather than being sent away by immigrant workers. Third, experienced workers developed specialized knowledge and skills that make farming more efficient, so the sector is better able to reach its wealth generating potential.

METRIC:
Age range of both farm owners and workers:

5	All working ages are well represented
4	Slightly skewed toward younger or older
3	Skewed toward younger or older
2	Almost entirely younger or older, but working age
1	Farmers primarily younger or older than working age

EXAMPLES:

PRODUCTION SECTOR

FARM WORKERS

Proportion of Income Spent on Food

RATIONALE:
Engel's curve is built upon observations that as income rises, the proportion of income spent on food declines. The proportion of income spent on food is another measure that reflects the wealth generation among farm workers.

METRIC:
Proportion of farm owners and workers' income that is spent on food.

5	Less than 10%
4	10-25%
3	25-40%
2	40-55%
1	More than 55%

EXAMPLES:

POST-HARVEST SECTOR PERFORMANCE

MARKETS

Farm-gate Price Compared to Historic High

RATIONALE:
If the sector is generating wealth, it is expected that sector will orient towards improving market access and development as opposed to competing for access and battling regulatory issues. This should be observable in stable or increasing farm-gate price.

METRIC:
The ratio of current farm-gate prices to the average of the three highest annual farm-gate prices in the past 10 years. Adjust by local CPI if inflation was significant.

5	Above 95%
4	85 to 95%
3	70 to 85%
2	50 to 70%
1	Below 50%

SCORING GUIDANCE:
In sectors where there are no historic data on prices, try to get participants to estimate general trends about whether prices have been rising or falling and by how much. If data are available, report in the excel worksheet labeled "Historical Data". Note that this is farm-gate prices and not wholesale or post-processing prices.

EXAMPLE:

POST-HARVEST SECTOR PERFORMANCE
MARKETS

Final Market Use

RATIONALE:
The use of the product that is finally consumed reflects the extent to which the sector, including farmers, processors and the supply chain, are maximizing the potential value.

METRIC:
The measure indicates the final market use of the product. Premium products are typically distinct to species, or species and source. This should be scored relative to global seafood trade, not to reflect the relative product quality within a given species.

5	Premium human consumption (premium quality and products)
4	High-value human consumption
3	Moderate-value human consumption
2	Low-value human consumption
1	Fish meal/animal feed/bait or non-consumptive

SCORING GUIDANCE:
Where a supply chain is diverse (i.e., there are multiple products such as frozen and fresh Atlantic salmon), score each and weight by value. In multispecies production systems, use the product form that dominates total value for each species and then weight each species by its contribution to total value across the sector. This metric is meant to be scored relative to the global seafood trade. This is not meant to reflect relative product quality within a given species (i.e. quality of Chilean salmon relative to Norwegian salmon). If we were scoring Chilean salmon then we should be scoring the product relative to the finest ahi tuna.

EXAMPLES:

POST-HARVEST SECTOR PERFORMANCE

MARKETS

International Trade

RATIONALE:

Maximizing wealth generation requires delivering the product to the people who value it the most. The level of exports reflects how well the sector has maximized wealth potential by accessing the market that is willing to pay the most for the product globally. Although exporting can be less profitable, such as high local demand for specialized products, generally the greatest returns are reached by a higher willingness to pay in international markets.

METRIC:

Percentage of the industry's value that is from fish exported to higher value international markets for consumption. Exports to markets that are not higher value should not count towards the score.

5	90-100% export
4	60-90% export
3	30-60% export
2	2-30% export
1	Virtually no export

SCORING GUIDANCE:

When products are exported for processing and then reimported for consumption this still counts as export. Note the emphasis on export to higher value markets. This means that Ghanaian aquaculture that distributes some smoked fish to Burkina Faso does not count as exports because this is not considered a higher value market. In addition, regional trade within a country should not be counted as exports.

The calculation should be: Total value of exports/Total value of production
Total value is calculated based on wholesale prices and quantities.

EXAMPLES:

1. About 5-10% of U.S. catfish production is exported. Exports achieve about the same price as product consumed domestically so this metric scores a 2.

POST-HARVEST SECTOR PERFORMANCE
MARKETS

Final Market Wealth

RATIONALE:
The income of the final consumers reflects the extent to which the sector is maximizing potential value. Products that are being sold in wealthier countries are competing favorably, reflecting high-quality, effective marketing and are drawing wealth to the sector.

METRIC:
Average per capita GDP of the consumer of the primary final product. If multiple important products or consumers/importers, weight by value:

5	Greater than 40,000USD
4	Greater than 19,400USD
3	Greater than 10,700USD
2	Greater than 3,700USD
1	Less than 3,700USD

EXAMPLE:
Bins are based the CIA's rankings of per capita GDP which can be found at https://www.cia.gov/library/publications/the-world-factbook/rankorder/2004rank.html. If there are many important product forms and importers, focus on the top five importers and weight the scores by value.

EXAMPLE:
1. Nearly all U.S. catfish production is consumed domestically. The per capita GDP of the U.S. is $59,500 and thus a score of 5.

POST-HARVEST SECTOR PERFORMANCE

MARKETS

Wholesale Price Compared to Similar Products

RATIONALE:

The extent to which the aquaculture sector is realizing its wealth generation is captured by comparing the price that sector receives with the price for substantially similar products from other sectors.

METRIC:

Ratio of average price for wholesale fish product from the fishery, to the global average price for similar products. Convert the price of fish to global currency for comparison (i.e. make sure that both prices are in USD when composing the ratio).

5	More than twice global average
4	120-200% global average
3	Within 20% of global average
2	50-80% of global average
1	Less than half global average

SCORING GUIDANCE:

For traded products, it is probably easiest to compare the export prices. The comparison between farmed and wild fish can be made if appropriate, but the comparison should be made to a similar product. Note that these are wholesale prices and not farm-gate prices. For multiproduct species, use wholesale price for the product form that dominates value.

EXAMPLES:

1. In the case of Atlantic salmon farmed in Norway, a similar product for comparison would be other farmed Atlantic salmon such as from Canada or Chile, and thus would score a 3.

POST-HARVEST SECTOR PERFORMANCE

MARKETS

Capacity of Firms to Export to the US and EU

RATIONALE:

Companies with unreliable, low quality or unsecure supply chains may not be able to export to the US or EU without detention. Access to these markets reflects the success of quality control systems and breadth of the market. It is also a measure of financial risk associated with international trade.

METRIC:

Percentage of a country's fish exports that meet US or EU health and labeling standards. This is usually a country level measure, though individual high-value industries sometimes develop their own supply chains; measure refers to all processing capacity for export, including to regional markets.

5	Over 90% meet US and EU health and labeling standards
4	50-90%
3	Less than 50%
2	A small amount of product meets US/EU standards
1	Banned in the US or EU, or cost of compliance with US/EU standards is prohibitive

SCORING GUIDANCE:

Note that this does not mean that the product necessarily *is* exported to the US/EU, it is based on the regulations and practices guiding the production and whether these satisfy the standards of these countries. There may be other importing countries with stricter standards than the US/EU; clearly, exceeding US/EU standards counts towards meeting them. In general, most fish that is dried/smoked in developing nations would not meet the standards for export.

EXAMPLES:

1. The USDA FSIS strictly regulates and monitors U.S. hybrid catfish products and thus ensuring that all products meet high quality health and labeling standards (score of 5).

POST-HARVEST SECTOR PERFORMANCE

MARKETS

Farm-gate to Wholesale Marketing Margins

RATIONALE:

The value-added by processing and marketing at the wholesale level is a direct measure of wealth accumulation in the processing sector. When compared across products, it can also represent how well the sector is realizing the maximum potential value of produced fish.

METRIC:

Increase in value of processed wholesale product from unprocessed farm-gate product. [Wholesale $/lb – Farm-gate $/lb]/(Farm-gate $/lb). Production of multiple product types should be scored by each type and then weighted by value.

5	More than 200% increase in value
4	100-200%
3	50-100%
2	10-50%
1	Less than 10% increase in value

SCORING GUIDANCE:

In sectors with multiple important product forms or multiple species, score each species/product and then weight by value (calculate the percentage of total revenue contributed by that species/product). Do this for the 5 dominant species/products that contribute the most to total revenue.

EXAMPLE:

POST-HARVEST SECTOR PERFORMANCE

MARKETS

Food Safety

RATIONALE:
Prevention of food safety issues is essential to attract consumer demand and develop strong and reliable markets.

METRIC:
Extent that the final products are safe to consume.

5	Safe to eat, no reported incidents in the past 5 years
4	Limited risk for food-bourne disease, non-lethal
3	Many cases of food-bourne disease, non-lethal
2	Serious illnesses that are potentially lethal, but no reported deaths
1	People die from consuming the product every year

SCORING GUIDANCE:

EXAMPLE:
1. There has been one recall on U.S. catfish in the past 5 years; however, it was a false positive, so this metric was scored a 5.

POST-HARVEST SECTOR PERFORMANCE

SUPPLY CHAIN PERFORMANCE

Processing Yield

RATIONALE:
Processing yield is a measure of the potential value of produced fish that is being realized as wealth. Yield will likely be higher in more efficient processing operations and those with a steady supply of fish. Scores will also be higher for processors who generate revenue streams for byproducts (bone, blood, etc.).

METRIC:
Ratio of actual processing yield (kilos/pounds) to the maximum yield technically achievable. Production of multiple product types should be scored by each type and then weighted by value.

5	At feasible frontier
4	Within 5% of the feasible frontier
3	Within 10%
2	Within 25%
1	Less than 75% of maximum yield

SCORING GUIDANCE:
In sectors where multi-species or multi-products are produced, score each species/product and then weight by value (calculate the percentage of total revenue contributed by that species/product) for the species/products that contribute the most to total revenue. When products go through multiple layers of processing, this metric refers to primary processing by the first buyer. The emphasis here is on the final weight compared to the technically feasible final weight and not on initial starting weight. Estimates of the technical frontier should consider the possibility of converting skin and bones into fish meal. However, if the primary product is fillets then there is no need to consider the yield on byproducts such as fish oil.

EXAMPLE:
1. Catfish processors obtain a 45% yield which is near the feasible frontier, and catfish byproducts are reduced into fish meal and oil, thus the U.S. catfish industry scores a 4.

POST-HARVEST SECTOR PERFORMANCE

SUPPLY CHAIN PERFORMANCE

Shrink

RATIONALE:
Shrink is the loss of target product that occurs from primary processing through to distribution and is a measure of the potential value that is lost. Product loss will likely be lower in more efficient processing operations and those with steady supply streams where there is more time to process better and develop customers who will pay premiums.

METRIC:
Percentage of product weight that is lost due to handling, spoilage, theft, bugs, or rats. This is very likely to be an estimate. Consider product that is lost between the farmer and the first wholesale buyer; do not consider product lost in retail stores or restaurant chains. Production of multiple product types should be scored by each type and then weighted by value.

5	Less than 5%
4	5-10%
3	10-25%
2	25-50%
1	More than 50%

SCORING GUIDANCE:
Shrink does not refer to the amount that fish weight changes as the product dries, it refers to lost/spoiled/mishandled product. This metric captures loss through the supply chain, until transferred to the retailer. In sectors where multi-species or multi-products are produced, score each species/product and then weight by value (calculate the percentage of total revenue contributed by that species/product), for the species/products that contribute the most to total revenue.

EXAMPLES:

POST-HARVEST SECTOR PERFORMANCE

SUPPLY CHAIN PERFORMANCE

Capacity Utilization Rate

RATIONALE:

A hindrance to wealth accumulation is an excess of processing capital. This may occur because production levels were much higher and it is difficult to downsize processing plants or because harvests are concentrated in a short period of time and economies are not to scale. Potential wealth is then consumed in maintaining a larger than necessary facility, or tying up capital in a facility that is not used to full capacity.

METRIC:

Days open for processing each year. Such days would not normally include religious or civic holidays, or weekly rest days. This should be full time employment days; when the plant is open but only operating at 10% capacity then this should only count for 10% of a day.

5	Virtually year-round
4	75-95% of days
3	50-75%
2	20%-50%
1	Less than 20%

SCORING GUIDANCE:

EXAMPLES:

POST-HARVEST SECTOR PERFORMANCE

SUPPLY CHAIN PERFORMANCE

Product Improvement

RATIONALE:
Processors can maximize the value of a product by marketing it with improvements that make it more appealing to consumers, who will then pay more for the product. Certification, value-enhancing branding, or value-added processing can increase wholesale and retail prices and thus improve the wealth of the sector.

METRIC:
Proportion of harvested meat weight going into certified, branded, fresh, fresh premium, portioned, live or value added products:

5	75-100% of landings are enhanced
4	50-75%
3	25-50%
2	1-25%
1	No landings have enhancements

SCORING GUIDANCE:
Preservation techniques that are used to keep product from spoiling, such as smoking, drying, salting and freezing, but do not add value should not count as product enhancement.

EXAMPLES:
1. Virtually all U.S. produced catfish goes to processing plants and is converted to frozen or fresh fillets, breaded nuggets or other value-added products, thus scoring a 5.
2. Eighty-five percent of farmed salmon in Norway is exported fresh, thus scoring a 5.

POST-HARVEST SECTOR PERFORMANCE

SUPPLY CHAIN PERFORMANCE

Proportion of Production Sold Fresh

RATIONALE:
Processors can maximize the value of a product by marketing it as fresh product, and thus make it more appealing to consumers who will then pay more for the product.

METRIC:
Proportion of production sold fresh leaving first processing.

5	Virtually all production is sold fresh
4	75-90%
3	50-75%
2	20-50%
1	Less than 20%

SCORING GUIDANCE:
This measure should consider the proportion of fish sold fresh after first processing.

EXAMPLES:
1. The majority of U.S. catfish leave the first processor as frozen fillets or breaded nuggets. Less than 20% is sold as fresh fillets.

POST-HARVEST SECTOR PERFORMANCE
SUPPLY CHAIN PERFORMANCE

Sanitation

RATIONALE:
Sanitation conditions in the harvest and processing areas serve as a direct measure of the community benefits that accrue to workers and to consumers than consume the products. Poor sanitation conditions reduce product quality and safety and can limit market access which prevents wealth benefits from being realized. The sanitation conditions within the processing plants also provide spillover benefits for the larger community as evidence from development economics which suggests that peer learning takes place when workers spread their increased knowledge of sanitation to neighbors and friends.

METRIC:
The state of the sanitation conditions in the production and processing areas. This measure is scored relative to global standards, not local standards.

5	Sanitation in harvest and processing areas meets global health standards
4	Basic treatment, but falls short of global standards
3	Human waste is adequately handled, but fish waste presents sanitation issues
2	Functional toilets are available, but fish or fish handlers exposed to untreated sewage
1	Functional toilets are not available in harvest or processing areas

SCORING GUIDANCE:
Pit latrines or toilets that are not improved, do not have proper drainage/sewage treatment, and do not allow for proper washing do not count as functional toilets.

EXAMPLES:
A processing facility that meets global health standards:

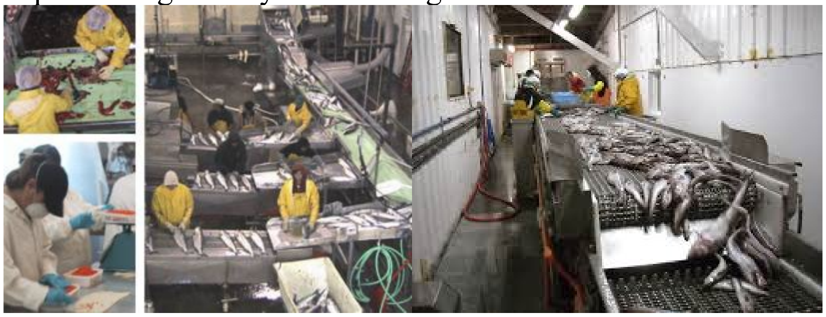

1. U.S. catfish processors are held to high health standards and regularly inspected by USDA FSIS, thus scoring a 5.

POST-HARVEST SECTOR PERFORMANCE
SUPPLY CHAIN PERFORMANCE

Local Support Businesses

RATIONALE:
The strength of supporting sectors is important to realize maximum economic gains in production and processing, and sourcing from local businesses is important to generate regional multipliers by keeping wealth in the region and improving community well-being. A profitable industry cluster can generate enough value to ensure all inputs to both the production and processing sectors are available for purchase locally.

METRIC:
Extent that supporting businesses are locally available. Support businesses are those that provide critical inputs (e.g., feed, seed, fertilizer, antibiotics, gear, technology, boat maintenance) or post-harvest functions (e.g., brokering, logistics).

5	All types of support are available locally
4	Some types of support are locally available
3	Few types of support are locally available
2	Local support is limited to variable inputs
1	Local support is not available

SCORING GUIDANCE:

EXAMPLES:

POST-HARVEST SECTOR PERFORMANCE

SUPPLY CHAIN PERFORMANCE

Availability of Support Businesses

RATIONALE:

The strength of supporting sectors is important to realize maximum economic gains in production and processing. Sales in the support sector are a direct measure of wealth accumulation in the support sector. However, they also reflect the ability of the industry to access and adopt new technology to make production more efficient and profitable, and the propensity for the industry to do so, as purchases of inputs support these businesses. Profitable industries with R&D and extension services will have a large number of specialized suppliers that conduct advanced services.

METRIC:

Extent that supporting businesses are available. Support businesses are those that provide critical inputs (e.g., feed, seed, fertilizer, antibiotics, gear, technology, boat maintenance) or post-harvest functions (e.g., brokering, logistics). This metric should measure whether inputs are available from local, national or international markets, whereas the previous metric should measure whether inputs are available locally.

5	All types of support are plentiful
4	Some types of support are capacity constrained or unavailable
3	Most types of support are capacity constrained or unavailable
2	Support limited to variable inputs
1	Industry support is not available

EXAMPLES:

POST-HARVEST SECTOR PERFORMANCE

SUPPLY CHAIN PERFORMANCE

Proportion of Feed Ingredients Sourced from Socially Sustainable Fisheries

RATIONALE:

Marine proteins from wild-capture fisheries, in addition to plant crops, are important feed components for most aquaculture industries. Improving socially responsibility in capture fisheries, agriculture and their supply chains are attracting greater attention from academics, NGOs, private and public institutions (Kittinger et al. 2017; Tickler et al. 2018). Feed fisheries are a potential area of the supply chain at increased risk for human rights violations.

METRIC:

Proportion of feed ingredients that are sourced from socially sustainable agriculture or aquaculture. Fisheries or crops harvested, processed or distributed under human rights or labor violations should count against the score.

5	100% of feed ingredients sourced from socially sustainable industries
4	90-99%
3	80-89%
2	70-79%
1	Less than 70% of feed ingredients sourced from socially sustainable industries

EXAMPLES:

POST-HARVEST SECTOR PERFORMANCE

POST-HARVEST ASSETS

Borrowing Rate Compared to Risk-Free Rate

RATIONALE:
The premium demanded by the capital market to make loans is a direct measure of financial risk in the sector. It is locally normalized to reflect the overall riskiness in the region and opportunities available to local capital.

METRIC:
Average ratio between the interest rate on loans made in the processing industry to risk-free rates over the last three years.

5	Less than 1.75; cf. 30-year conforming mortgage
4	Less than 2.5; cf. personal bank loan
3	Less than 4; cf. good credit card rates
2	Less than 7; cf. bad credit card rates
1	Greater than 7; usury

SCORING GUIDANCE:
Calculation: Interest rate in the processing industry / Risk-free interest rate (average over last 3 years).

Note that if businesses can access international credit markets, then the international risk-free rate (US 10-year Treasury bill) is an appropriate comparison, but if businesses are forced to use local credit markets then the benchmark should be local risk-free rates. Typically national/municipal government bonds will be the best representative of local risk-free rates. When scoring, it is often easier to ask the next question about the source of capital funds first and then ask about the rates that they pay. As long as there are credit transactions this metric should not be NA; strive to get some estimate of the interest rates that processors pay.

EXAMPLES:

POST-HARVEST SECTOR PERFORMANCE

POST-HARVEST ASSETS

Source of Capital

RATIONALE:

The availability of lending capital from particular sources is a direct measure of how the capital market assesses risk in the processing sector. If a certain type of lender or investor is not willing to make capital available in the processing sector at any price, it reveals that it is much riskier than other available investments.

METRIC:

A measure of the category of lenders or investors that are most typically used in the processing sector. Second scoring method offered if the supply chain (e.g., processors further up the supply chain, parent company, exporters) are the primary source of capital.

5 Unsecured business loans from banks/public stock offering;

4 Secured business loans from banks/venture capital; investment from elsewhere in the supply chain

3 Loans from banks secured by personal (not business) assets/Government subsidized private lending/Government-run loan programs/International aid agencies; secured loans from elsewhere in supply chain

2 Microlending/Family/Community-based lending; loans from supply chain significantly reduce margins

1 Mafia/No capital available; exploitative relationship from elsewhere in supply chain

SCORING GUIDANCE:

Please note in the worksheet explanation which scoring method was used (i.e., whether or not the supply chain is the primary source of capital). Processors could be obtaining credit from middlemen, fish traders or their parent company.

EXAMPLES:

POST-HARVEST SECTOR PERFORMANCE

POST-HARVEST ASSETS

Age of Facilities

RATIONALE:

The age of the facilities used in processing fish, primarily processing plants and storage facilities, reflects several dimensions of sector wealth. First, it is a direct measure of wealth that has accumulated from the sector and reinvested in capital. Second, it is a measure of the potential wealth of the sector, as newer facilities will be more efficient and less costly to operate. Third, if processors are willing to invest in new capital, it reflects an assessment that the sector will be profitable in the future.

METRIC:

Average age of the key durable processing capital unit (plants).

5 Capital is new or up to date
4 Capital is older but well maintained, e.g., freshly painted
3 Capital is moderately well maintained
2 Maintenance is poor
1 Serious concerns about functionality and safety

SCORING GUIDANCE AND EXAMPLES:

POST-HARVEST SECTOR PERFORMANCE

PROCESSING MANAGERS

Earnings Compared to Regional Average Earnings

RATIONALE:

Processing manager earnings are a direct measure of the type of workers that are attracted to the industry. Earnings are normalized by average regional earnings to reflect whether the sector is able to attract the most talented workers. It also reflects how well the industry is generating wealth relative to the local standards.

METRIC:

Ratio of annual earnings per owner/manager to the regional average earnings. This metric can include wealth accumulated to traders/middlemen if they represent an important part of the supply chain. Note that this is earnings from all sources, not just fish farming and is compared to the regional/national levels where the owner/manager conducts the majority of their economic activity.

5	More than 50% above the regional average
4	Between 10 and 50% above regional average
3	Within 10% of the regional average
2	Between 50 and 90% of the regional average
1	Less than half of the regional average

SCORING GUIDANCE:

This metric is meant to measure what type of people the sector attracts; thus we consider all income for an entire year from any sources. These earnings should be compared to regional/national levels depending on the economic sphere of the owners. Economic sphere is defined as the region where the owners conduct the majority of their economic activity, i.e. the village if all economic activity is within the village, but the nation if the owner participates in national markets as a consumer. Make sure that this variable and the following metrics in the owner category are scored for the people who own the means of processing; this could be traders if the primary market is fresh.

EXAMPLES:

Post-Harvest Sector Performance

Processing Managers

Manager Wages Compared to Non-Fish Farming Wages

RATIONALE:
Processing owner or manager wages are a direct measure of wealth that accumulates to managers. Wages are normalized by wages typical of alternate jobs within the region to provide an indicator of the relative standard of living afforded to workers. It also reflects whether the industry is attracting the most skilled managers.

METRIC:
Ratio of processing owner's average daily wage in this industry to average daily wage of the owner's alternative occupation that within their economic sphere (e.g., jobs in the village that the owner qualifies for if all economic activity is within the village, but if labor markets are fluid then this should be national average wage for jobs that the owners/managers expect to be able to obtain).

5	More than 50% above the alternative wage
4	Between 10 and 50% above the alternative wage
3	Within 10% of the alternative wage
2	Between 50 and 90% of the alternative wage
1	Less than half of the alternative wage

SCORING GUIDANCE:
This is meant to measure the average personal opportunity cost of participating in the industry, thus the alternative wage should be the answer to the question of "If you couldn't process fish how much would you get paid?" Look at the average daily wage for processing owners/managers when participating in the sector and then compare it to the wage in their next best alternative. If the processing owners think that without the industry they would be lawyers then look up the wages of lawyers, but if they think that they would be subsistence farming then compare it to that wage. Again, this should all be relative to wages within their economic sphere so consider national wages if labor markets are fluid, but restrict the comparison to wages within the village/region if owners seldomly leave their local community and do not have the means to do so.

EXAMPLES:

POST-HARVEST SECTOR PERFORMANCE
PROCESSING MANAGERS

Education Access

RATIONALE:
A community that is successful in extracting wealth from the industry will be able to provide high levels of education.

METRIC:
This metric is based on the highest level of education that is politically and financially accessible (available and affordable) to families of processing owners/managers. This is not based on the actual attainment levels of current processing owners/managers and families.

5	Higher education is accessible
4	High school level education or advanced technical training is accessible
3	Middle school level education or simple technical training is accessible
2	Basic literacy and arithmetic training is accessible
1	Formal education is not accessible

SCORING GUIDANCE:
This metric is meant to capture whether processing owners/managers can afford to provide education for their children. Just because there is a high school in the village does not mean that they can afford to send their children – consider school fees, tuition, and opportunity cost. Note that learning to fish or process fish as an on the job apprenticeship does not count as formal technical training. Advanced technical training involves science/technology and most apprenticeships in LDCs do not count and should be classified as simple technical training at the most.

EXAMPLES:

POST-HARVEST SECTOR PERFORMANCE

PROCESSING MANAGERS

Access to Health Care

RATIONALE:
A community that is successful in extracting wealth from the industry will be able to provide high levels of healthcare, ensuring quality of life and reducing health risk.

METRIC:
This metric is based on the quality of health care that is politically and financially accessible (available and affordable) to processing owners and their families.

5	Global standard treatment for illness is accessible
4	Licensed doctors provide trauma, surgical and drug treatments
3	Nurses or medical practitioners provide emergency and routine drug treatments
2	Basic and simple drug treatment is accessible
1	Medical or drug treatment is not accessible

SCORING GUIDANCE:
This metric is meant to capture whether processing owners can afford to provide health care for themselves and their children. Just because there is a clinic in the village does not mean that they can afford it – consider medical fees, travel time and opportunity cost. The WHO's health service coverage index is used as a basis for identifying whether local care facilities are capable of providing global standard care (http://apps.who.int/gho/portal/uhc-cabinet-wrapper-v2.jsp?id=1010501). If there is a global-standard hospital located in a major city a day's journey away, then global standard treatment for illness is not fully accessible because if there was a major trauma then the injured party would likely die before reaching medical assistance (unless there is a Coast Guard helicopter assigned to transport injured workers/family members). Score based on the health facilities that are used most frequently for routine procedures and somewhat urgent issues. Note that the scores for this metric are likely to be the same across processing owners and workers if they and their families live in the same communities and make enough money to afford the health care that is offered there. It will be different if owners/managers can afford to travel to urban centers for emergency or surgical procedures while workers cannot.

EXAMPLES:

POST-HARVEST SECTOR PERFORMANCE

PROCESSING MANAGERS

Social Standing of Processing Managers

RATIONALE:

This is a proxy for income associated with owning a processing plant, which may be much easier to collect than actual wage information. Social standing reflects whether the sector is able to attract the most talented workers in the community and signals the extent of wealth generation relative to local standards.

METRIC:

This metric is based on the social standing of processing owners/managers within the community where they spend the majority of their time.

5	Among the most respected in the community, comparable with civic and religious leaders and professionals, such as doctors and lawyers
4	Comparable to management and white collar jobs
3	Comparable to skilled labor jobs
2	Comparable to unskilled blue collar or service jobs
1	Among the least respected, such as slaves or indentured servants

SCORING GUIDANCE:

This is meant to reflect the amount of esteem that processing owners receive in their local community. Make sure that answers for this metric make sense relative to the social status of the other fishery occupations (farmers, farm workers, and processing workers). Consider their social status within their primary community. The comparison group should be the region where they spend the majority of their time and income.

EXAMPLES:

POST-HARVEST SECTOR PERFORMANCE

PROCESSING MANAGERS

Nonresident Ownership of Processing Capacity

RATIONALE:

Benefits to the community or region relies on the ability to maintain local multipliers by keeping wealth in the region. Non-resident capital reflects that wealth will be leaving the region and failing to boost the regional economy. In developing economies, it may also reflect an inability of locals to generate sufficient capital to process.

METRIC:

Proportion of farm-gate value processed by regionally owned processing capital. “Local” is defined as coming from, and spending their earnings within, the local community. Nationals who are transient nonresidents, or considered outsiders in the community, are not local.

5	95-100% local
4	70-95% local
3	35-70% local
2	5-35% local
1	Virtually no local processing ownership

SCORING GUIDANCE AND EXAMPLES:

POST-HARVEST SECTOR PERFORMANCE
PROCESSING WORKERS

Earnings Compared to Regional Average Earnings

RATIONALE:
Processing worker earnings are a direct measure of the type of agents who are attracted to the industry. Earnings are normalized by average regional earnings to reflect whether the sector is able to attract the most talented workers. It also reflects how well the industry is generating wealth relative to the local standards.

METRIC:
Ratio of annual earnings per processing worker to the average earnings in the region. Note that this is earnings from all sources, not just processing, and is compared to the regional/national levels where the processing worker conducts the majority of their economic activity.

5	More than 50% above the regional average
4	Between 10 and 50% above the regional average
3	Within 10% of the regional average
2	Between 50 and 90% of the regional average
1	Less than half of the regional average

SCORING GUIDANCE:
This metric is meant to measure what type of people the sector attracts; thus we consider all income for an entire year from any sources. These earnings should be compared to regional/national levels depending on the economic sphere of the workers. Economic sphere is defined as the region where the workers conduct the majority of their economic activity, i.e. the village if all economic activity is within the village, but the nation if the worker participates in national markets as a consumer.

Make sure that this variable and the following metrics in the worker category are scored for the people who work for wages and not those who own the processing facilities or who are self-employed and engaged in trading/selling the fish.

EXAMPLES:

POST-HARVEST SECTOR PERFORMANCE

PROCESSING WORKERS

Worker Wages Compared to Non-Fish Farming Wages

RATIONALE:

Processing worker wages are a direct measure of wealth that accumulates to processing workers. It is normalized by wages typical of alternate jobs within the region to provide an indicator of the relative standard of living afforded to workers. It also reflects whether the industry is able to attract the most skilled workers.

METRIC:

Ratio of the processing worker's average daily wage in this industry to the average daily wage of the worker's alternative occupation that is within their economic sphere. If all economic activity is within the village, the comparison is to jobs in the village that the worker qualifies for, but if the worker participates in national markets as a consumer and labor markets are fluid, then the comparison is with jobs within the nation that the worker qualifies for.

5	More than 50% above the alternative wage
4	Between 10 and 50% above the alternative wage
3	Within 10% of the alternative wage
2	Between 50 and 90% of the alternative wage
1	Less than half of the alternative wage

SCORING GUIDANCE:

This is meant to measure the average personal opportunity cost of participating in the sector; thus the alternative wage should be the answer to the question "If you couldn't work in processing, how much would you get paid?" Look at the average daily wage for processing workers when participating in the sector and then compare it to the wage in their next best alternative. If the workers think that without the processing wages they would be construction workers, then score based on construction worker wages, but if they think that they would be subsistence farming, then compare it to that wage. Again, this should all be relative to wages within their economic sphere so consider national wages if labor markets are fluid, but restrict the comparison to wages within the village/region if workers seldom leave their local community and do not have the means to do so.

EXAMPLES:

POST-HARVEST SECTOR PERFORMANCE

PROCESSING WORKERS

Education Access

RATIONALE:
A community that is successful in extracting wealth from the industry will be able to provide high levels of education.

METRIC:
This metric is based on the highest level of education that is politically and financially accessible (available and affordable) to families of processing workers. This is not based on the actual attainment levels of processing workers and families.

5 Higher education is accessible
4 High school level education or advanced technical training is accessible
3 Middle school level education or simple technical training is accessible
2 Basic literacy and arithmetic training is accessible
1 Formal education is not accessible

SCORING GUIDANCE:
This measure is meant to capture whether processing workers can afford to provide education for their children. Just because there is a high school in the village does not mean that they can afford to send their children – consider school fees, tuition, and opportunity cost. Note that learning to fish or process fish as an on the job apprenticeship does not count as formal technical training. Advanced technical training involves science/technology and most apprenticeships in LDCs do not count and should be classified as simple technical training at the most.

EXAMPLES:

POST-HARVEST SECTOR PERFORMANCE

PROCESSING WORKERS

Access to Health Care

RATIONALE:
A community that is successful in extracting wealth from the industry will be able to provide high levels of healthcare, ensuring quality of life and reducing health risk.

METRIC:
This metric is based on the quality of health care that is politically and financially accessible (available and affordable) to processing workers and their families.

5	Global standard treatment for illness is accessible
4	Licensed doctors provide trauma, surgical and drug treatments
3	Nurses or medical practitioners provide emergency and routine drug treatments
2	Basic and simple drug treatment is accessible
1	Medical or drug treatment is not accessible

SCORING GUIDANCE:
This metric is meant to capture whether processing workers can afford to provide health care for themselves and their children. Just because there is a clinic in the village does not mean that they can afford it – consider medical fees, travel time and opportunity cost. The WHO's health service coverage index is used as a basis for identifying whether local care facilities are capable of providing global standard care (http://apps.who.int/gho/portal/uhc-cabinet-wrapper-v2.jsp?id=1010501). If there is a global-standard hospital located in a major city a day's journey away, then global standard treatment for illness is not fully accessible because if there was a major trauma then the injured party would likely die before reaching medical assistance (unless there is a Coast Guard helicopter assigned to transport injured workers/family members). Note that the scores for this metric are likely to be the same across processing owners and workers if they and their families live in the same communities and make enough money to afford the health care that is offered there. It will be different if owners/managers can afford to travel to urban centers for emergency or surgical procedures while workers cannot.

EXAMPLES:

Social Standing of Processing Workers

RATIONALE:
This is a proxy for income associated with working in processing plants, which may be much easier to collect than actual wage information. Social standing reflects whether the sector is able to attract the most talented workers in the community and signals the extent of wealth generation relative to local standards.

METRIC:
Measure is based on the social standing of processing workers within the community where they spend the majority of their time and income.

5 Among the most respected in the community, comparable with civic and religious leaders and professionals, such as doctors and lawyers
4 Comparable to management and white collar jobs
3 Comparable to skilled labor jobs
2 Comparable to unskilled blue collar or service jobs
1 Among the least respected, such as slaves or indentured servants

SCORING GUIDANCE:
This is meant to reflect the amount of esteem that processing workers receive in their local community. Make sure that answers for this metric make sense relative to the social status of the other related occupations (processing owners, farmers and farm workers). Consider their social status within their primary community. The comparison group should be the region where they spend the majority of their time and income.

EXAMPLES:

POST-HARVEST SECTOR PERFORMANCE

PROCESSING WORKERS

Proportion of Nonresident Employment

RATIONALE:
Benefits to the community or region relies on the ability to maintain local multipliers by keeping wealth in the region. A large portion of non-resident employment reflects that wealth will be leaving the region and failing to boost the regional economy. In developing economies, it may also reflect an inability of locals to generate sufficient capital.

METRIC:
Proportion of processing workers who are local. “Local” is defined as coming from, and spending their earnings within, the local community. Nationals who are transient nonresidents, or considered outsiders in the community, are not local.

5	95-100% local
4	71-95% local
3	36-70% local
2	5-35% local
1	Virtually no local processing workers

SCORING GUIDANCE AND EXAMPLES:

POST-HARVEST SECTOR PERFORMANCE

PROCESSING WORKERS

Worker Experience

RATIONALE:
The rate at which workers turn over is an indirect measure of several key variables. First, it reflects wealth accumulation to workers because a worker will only stay if the wage is comparable to, or better than, other obtainable jobs. Second, worker longevity often means they are residents of the community and thus their earnings stay in the community and are spent locally rather than being sent away by immigrant workers. Third, experienced workers developed specialized knowledge and skills that make farming more efficient, so the sector is better able to reach its wealth generating potential.

METRIC:
Average years of experience of processing workers.

5	More than 10 years (skilled career workers)
4	5-10 years
3	3-5 years
2	1-3 years
1	0 full years of experience (mostly new workers each season)

EXAMPLE:

AQUACULTURE PERFORMANCE INDICATORS

INPUTS

ENABLING THE CREATION OF SUSTAINABLE INCOMES AND ECOSYSTEM HEALTH

This section identifies 66 inputs that may lead to the generation of sustainable livelihoods and healthy ecosystems (see Table 2 below). Each metric (last column in Table 2) is individually explained in the following pages and is accompanied by examples from the set of existing case studies. In addition, each metric includes a rationale that demonstrates the existing theoretical or empirical arguments from the literature that justify the inclusion of the metric.

Supplementary Table 2. Aquaculture Performance Indicators–Inputs

Dimension	Component	Metric
Macro Factors	National Environment	Environmental Performance Index (EPI)
	Exogenous Factors	Natural Disasters and Catastrophes<br>Drought<br>Pollution Shocks and Accidents<br>Level of Chronic Pollution - Production Effects<br>Level of Chronic Pollution - Consumption Effects
	Governance	Governance Quality<br>Governance Responsiveness
	Economic Conditions	Index of Economic Freedom<br>Gross Domestic Product (GDP) Per Capita
Property Rights	Land Rights	Proportion of Production with Property or Lease Right<br>Transferability Index<br>Security Index<br>Durability Index<br>Flexibility Index<br>Exclusivity Index
Co-management	Collective Action	Proportion of Farmers in Industry Organizations<br>Farmer Organization Influence on Management<br>Farmer Organization Influence on Business & Marketing
	Participation	Days in Stakeholder Meetings<br>Industry Financial Support for Management
	Community	Leadership<br>Social Cohesion
	Gender	Business Management Influence<br>Resource Management Influence<br>Labor Participation in Production Sector<br>Labor Participation in Processing Sector
Management	Management Inputs	Management Expenditure Compared to Farm-Gate Value<br>Enforcement Capability<br>Management Jurisdiction<br>Generations separated by selective breeding<br>Coordination of regulatory authorities<br>Level of Subsidies<br>Percentage of marine ingredients<br>Traceability of feed inputs<br>R&D<br>Private R&D
	Data	Biological data collection<br>Market and economic data
	Management Methods	Regional disease control<br>Genetic management<br>Discharge/effluent control<br>Antibiotic use<br>Antibiotic use practices<br>Food safety services<br>Animal welfare/handling practices<br>Damage compensation/management<br>Access to Water<br>Land or water zoning/management
Supply Chain	Markets & Market Institutions	Transparency of Farm-gate price<br>Availability of Farm-gate Price & Quantity Information<br>Number of Buyers<br>Degree of Vertical Integration<br>Level of Tariffs<br>Level of Non-tariff Barriers<br>Contribution to Economy
	Infrastructure	International Shipping Service<br>Road Quality Index<br>Technology Adoption in Production<br>Technology Adoption in Processing<br>Extension Service<br>Reliability of Utilities/Electricity<br>Access to Ice & Refrigeration
Production	Producer characteristics	Scale of farm<br>Integrated culture<br>Production under contract farming

MACRO FACTORS

GENERAL ENVIRONMENTAL PERFORMANCE

Environmental Performance Index (EPI)

RATIONALE:
Wealth creation is dependent on the general condition of the environment and environnmental regulation. An Environmental Performance Index (EPI) has been developed to evaluate: 1) environmental health and 2) ecosystem vitality (Esty et al. 2008) at the national level for the majority of countries around the globe.

METRIC:
The EPI considers factors such as disease, water quality, air pollution, biodiversity, natural resources and climate change. The EPI ranges from 1-100. Score is by 2020 EPI quintiles:

5	EPI of 61.6-100
4	46.5-61.5
3	39.2-46.4
2	32.8-39.1
1	1-32.7

SCORING GUIDANCE:
Visit the EPI website at https://epi.envirocenter.yale.edu/epi-topline. The score used should be the aggregate "Environmental Performance Index" score and not the score for one of the individual components. Make sure that the raw score is not reported, but instead place it within one of the above bins and determine whether this is a score of 1-5. If the country is not given an EPI score, a best guess can be made based on prevailing environmental conditions and conditions in neighboring countries. If the industry is transnational, then weight the EPI scores of each country depending on the portion of production value that occur in the country.

MACRO FACTORS

EXOGENOUS ENVIRONMENTAL FACTORS

Natural Disasters and Catastrophes

RATIONALE:
Even a well-managed aquaculture sector can fail to accumulate wealth if exogenous events or conditions threaten production or production capital/capacity. This metric is intended primarily to identify when management inputs will not be correlated with outcomes for reasons exogenous to the aquaculture sector. In particular, this metric incorporates the effect of natural disasters such as hurricanes on value of production.

METRIC:
The extent to which production values are affected by natural disasters such as earthquakes, volcanoes, tsunamis, hurricanes and typhoons. These are typically one-time events, not long-term ecosystem scale shifts induced by climate change (e.g., shifts in temperature or salinity). Production values can be affected directly by production loss or damage to production capacity.

5	Production value unaffected by disaster
4	Production value reduced by less than 10%
3	Production value reduced by 10-30%
2	Production value reduced by more than 30%
1	Production value almost completely eliminated by disaster

SCORING GUIDANCE:
This score should be based on empirical or anecdotal evidence of natural disasters affecting production values. Even if an earthquake had no effect on crop loss because they can submerge offshore net pens, it may have destroyed tender vessels or other equipment that would lead to a reduction in the ability to bring the the fish to market. Note that this does not refer to potential or theoretical natural disasters but only to the effects of actual historic events. Natural disasters that occurred in the past should only be included in the score if they continue to affect production values in the present.

EXAMPLES:

MACRO FACTORS

EXOGENOUS ENVIRONMENTAL FACTORS

Drought

RATIONALE:
Even a well-managed aquaculture sector can fail to accumulate wealth if exogenous events or conditions threaten production or production capital/capacity. This metric is intended primarily to identify when management inputs will not be correlated with outcomes for reasons exogenous to the aquaculture sector. In particular, this metric incorporates the effect of drought on production values.

METRIC:
The extent to which production value in the reference year is affected by drought.

5	Production value unaffected by shocks
4	Production value reduced by less than 10%
3	Production value reduced by 10-30%
2	Production value reduced by more than 30%
1	Production value almost completely closed by shocks

SCORING GUIDANCE:
This score should be based on empirical or anecdotal evidence of drought affecting production values. Note that this should not be based on the potential for adverse effects of drought, but only on the effect of actual droughts that have occurred. Droughts that occurred in the past should only be included in the score if they continue to affect production values in the present.

EXAMPLES:

Macro Factors

Exogenous Environmental Factors

Pollution Shocks and Accidents

RATIONALE:
Even a well-managed aquaculture sector can fail to accumulate wealth if exogenous events or conditions threaten production or production capital/capacity. This metric is intended primarily to identify when other management inputs will not be correlated with outcomes for reasons exogenous to the aquaculture sector. In particular, this metric incorporates the effect of pollution shocks that have been shown to affect the value of production independent of management action.

METRIC:
The extent to which production value in the reference year is affected by pollution shocks, such as oil spills, industrial accidents, peak runoff events, or theft. These are typically one-time events, not chronically high levels of pollution.

- 5 Production value unaffected by shocks
- 4 Production value reduced by less than 10%
- 3 Production value reduced by 10-30%
- 2 Production value reduced by more than 30%
- 1 Production value almost completely closed by shocks

SCORING GUIDANCE:
This score should be based on empirical or anecdotal evidence of pollution shocks affecting production values. Note that this should not be based on the potential for theoretical accidents, but only on the effect of actual shocks that have occurred. Also note that the influence of runoff or dumping that occurs every year should not show up in this metric; that is considered chronic pollution and it is included in the next metric. Oil spills and other industrial accidents that occurred in the past should only be included in the score if they continue to affect production values in the present.

EXAMPLES:

MACRO FACTORS

EXOGENOUS ENVIRONMENTAL FACTORS

Level of Chronic Pollution (Production Effects)

RATIONALE:

Even a well-managed aquaculture sector can fail to accumulate wealth if exogenous events or conditions threaten production or production capital/capacity. This metric is intended primarily to identify when management inputs will not be correlated with outcomes for reasons exogenous to the aquaculture sector. In particular, this metric incorporates the effect of chronic pollution that has been shown to affect production values.

METRIC:

Extent to which chronic pollution, such as from industrial or agricultural runoff, affects production. Chronic pollution can be either always present, or frequently recurring, such as after each moderate rainfall. This measure should reflect how chronic pollution of land or water influences production of the organism. This should not consider the effect of chronic pollution on consumptive behavior which is captured below.

5	Not detectable
4	Minimal detectable levels
3	High levels detected
2	Pollution affects growth of organisms
1	Pollution leads to severe decline in production

SCORING GUIDANCE:

This metric should be based on empirical or anecdotal evidence of chronic pollution affecting production of the organism. Note that the emphasis for this metric is on the impact of pollution on the ability to produce the organism; demand/consumption effects will be captured in the next metric. Also note that this metric should not include the impact of one-time pollution shocks or accidents as that was captured in the last metric.

EXAMPLES:

Macro Factors

Exogenous Environmental Factors

Level of Chronic Pollution - Consumption Effects

RATIONALE:

Even a well-managed aquaculture sector can fail to accumulate wealth if exogenous events or conditions threaten production or production capital/capacity. This metric is intended primarily to identify when management inputs will not be correlated with outcomes for reasons exogenous to the aquaculture sector. In particular, this metric incorporates the effect of chronic pollution that has been shown to affect production values through decreased consumer demand.

METRIC:

The extent that chronic pollution limits consumption. Chronic pollution can be either always present, or frequently recurring, such as after each moderate rainfall. Note that the emphasis for this metric is on how consumers perceive the fish as result of information about pollution; the effects of pollution on the ability to produce the species was captured in the previous metric.

5	No consumption affected
4	Minimal consumption affected
3	Official consumption advisories
2	Temporarily ban harvest for consumption
1	Completely closed for consumption

SCORING GUIDANCE:

This metric should be based on empirical or anecdotal evidence of chronic pollution affecting consumption. Note that the emphasis for this metric is on how consumers perceive the fish as result of information about pollution; the effects of pollution on production were captured in the previous metric. Also note that this metric should not include the impact of one-time pollution shocks or accidents as that was captured in a previous metric. If local markets are unaffected by pollution because local consumers are unaware of the threat or do not care about its health effects, the industry should obtain a high score. However, if there is a sense that export is impeded because foreign market consumers have concerns about pollution (and this is the binding constraint, rather than health codes or market limitations) then the score should reflect this.

EXAMPLES:

MACRO FACTORS
GOVERNANCE

Governance Quality

RATIONALE:
Good governance, starting with a functional central government, can be an essential condition for sustainable aquaculture and wealth creation. The World Bank has developed a Worldwide Governance Indicator which considers six dimensions: Voice & Accountability, Political Stability and Absence of Violence/Terrorism, Government Effectiveness, Regulatory Quality, Rule of Law and Control of Corruption (Kaufman, et al. 2008).

METRIC:
Average of four indicators in the World Bank's Governance Indicators, each scored [-2.5, 2.5]

- Government Effectiveness
- Regulatory Quality
- Rule of Law
- Control of Corruption

5	Above 0.92 (highest-performing 2010 quintile)
4	0.10 to 0.92
3	-0.43 to 0.10
2	-0.81 to -0.43
1	Below -0.81 (lowest-performing 2010 quintile)

SCORING GUIDANCE:
Visit the website http://info.worldbank.org/governance/wgi/index.aspx#reports. Click on "Interactive Data Access" and then the "Table View" tab. Select the country and the most recent year of data available from the drop down menu. Average the "Governance Score" for the four indicators listed above (Note: that some indicator values may be negative). Make sure that the raw score is not reported, but instead place it within one of the above bins and determine whether this is a score of 1-5. If the industry is transnational then weight the WGI scores of each country depending on the portion of total revenue that occurs in the country.

MACRO FACTORS

GOVERNANCE

Governance Responsiveness

RATIONALE:
Good governance, starting with a functional central government, can be an essential condition for sustainable aquaculture and wealth creation. The World Bank has developed a Worldwide Governance Indicator which considers six dimensions: Voice & Accountability, Political Stability and Absence of Violence/Terrorism, Government Effectiveness, Regulatory Quality, Rule of Law and Control of Corruption (Kaufman, et al. 2008).

METRIC:
Average of two indicators in the World Bank's Governance Indicators, each scored [-2.5, 2.5]

- Voice and Accountability
- Political Stability

5	Above 0.96 (highest-performing 2010 quintile)
4	0.41 to 0.96
3	-0.24 to 0.41
2	-0.82 to -0.24
1	Below -0.82 (lowest-performing 2010 quintile)

SCORING GUIDANCE:
Visit the website http://info.worldbank.org/governance/wgi/index.aspx#reports. Click on "Interactive Data Access" and then the "Table View" tab. Select the country and the most recent year of data available from the drop down menu. Average the "Governance Score" for the two indicators listed above (Note: that some indicator values may be negative). Make sure that the raw score is not reported, but instead place it within one of the above bins and determine whether this is a score of 1-5. If the industry is transnational then weight the WGI scores of each country depending on the portion of total revenue that occurs in the country.

MACRO FACTORS

ECONOMIC CONDITION

Index of Economic Freedom

RATIONALE:

Wealth creation is dependent on the institutional setting and economic conditions in a given country. The Heritage Foundation/Wall Street Journal's Index of Economic Freedom (IEF) reflects the overall economic freedom of the nation within the aquaculture sector operates (Miller and Holmes, 2009). The *Index of Economic Freedom* includes 10 broad institutional factors:

- Business freedom
- Trade freedom
- Fiscal freedom
- Government size
- Monetary freedom
- Investment freedom
- Financial freedom
- Property rights
- Freedom from corruption
- Labor freedom

Construction of the index relies on several other studies for its data sources, including the World Bank's *Doing Business* Economist Intelligence Unit (The World Bank 2009a), the US Department of Commerce, the World Bank's *World Development Indicators* (The World Bank, 2009b), Eurostat, International Monetary Fund reports, Transparency International's, *Corruption Perceptions Index* (Transparency International, 2009) and several other documents.

METRIC:

The score from the Heritage Foundation's Index of Economic Freedom for the country within the industry operates. Bins defined based on 2010 percentiles.

5	IEF of 69.2-100
4	62.5-69.1
3	57.1-62.4
2	50.5-57.0
1	1-50.5

SCORING GUIDANCE:

Visit the website http://www.heritage.org/index/default then click on "Explore the Data" and find the country in the list. Consult the "Overall Score" column. Make sure that the raw score is not reported, but instead place it within one of the above bins and determine whether this is a score of 1-5. If the country does not have an overall score then average the dimensions for which it is scored, but make note of which columns are missing data in the worksheet. If the industry is transnational then weight the IEF scores of each country depending on the portion of total revenue that occurs in the country.

Macro Factors
Economic Condition

Gross Domestic Product (GDP) Per Capita

Rationale:
Richer nations are more likely able to afford the institutions and technological factors that are necessary for effective management and sustainable wealth generation.

Metric:
Country's per capita GDP on a purchasing power parity basis.

5	Greater than 40,000USD
4	Greater than 19,400USD
3	Greater than 10,700USD
2	Greater than 3,700USD
1	Less than 3,700USD

Scoring Guidance:
Find the CIA's rankings of per capita GDP at https://www.cia.gov/library/publications/the-world-factbook/rankorder/2004rank.html. Bins are based on the 2017 quintiles of the CIA's rankings of per capita GDP. If the industry is transnational then weight the GDP of each country depending on the portion of total revenue that occurs in the country.

Property Rights

Land Rights

Proportion of Production with Property or Lease Rights

Rationale:

Land tenure systems determine who can use what resources, for how long, and under what conditions. The strength of property rights is believed to be correlated with decision-making power and wealth generation. Property rights to land are defined as the institutions that grant exclusive rights to control land. These rights are sometimes granted through land ownership, but can be temporarily distributed using land leases. Here, the definition of land includes land, the water column, submerged land or other spatially defined areas.

Metric:

The proportion of total production value where farmers have property or lease rights to land.

5	Virtually all
4	70-95%
3	35-70%
2	5-35%
1	Virtually none

Scoring Guidance:

Property rights are defined here as the institutions that grant ownership or exclusive rights and control of land. This can be a temporary or permanent right to hold and control land. Having property rights does not necessarily infer ownership; land leases are considered short-term property rights. Ownership of the land is typically a strong property right, whereas leasing land is typically a weaker and short-term property right. Property rights can be defined legally through official laws or policies or through informal customs, and the property right can be held by individuals or communities such as in some collective farming systems.

Examples:

1. Nearly all U.S. catfish farmers own the land where farming activities are conducted, thus scoring a 5 on this metric.
2. In the case of Atlantic salmon farm in nearshore pens, the farmers hold a lease granting them control of the surface water and water column, thus also scoring a 5 on this metric.

LAND RIGHTS

PROPERTY RIGHTS

Transferability Index

RATIONALE:

Transferability of rights is essential for a functioning market to allocate resources to their best use. If property rights are not transferable, financing can be undermined because the property right may not be accepted as collateral. This measure should reflect the extent that the property right is transferable through sale and if markets are efficient and transparent. Limits on how much property one entity can hold should count against the score if they are restricting growth of enterprises.

METRIC:

Extent to which the property right is transferable. NA if there is no property right.

5	Very Strong: Fully transferable through well-established, efficient market institutions
4	Strong: Fully transferable, but institutions are poor or illiquid
3	Moderate: Transferable, but with severe restrictions on who can hold, or how much
2	Weak: Transferable only under highly restricted and limited conditions
1	Property rights not transferable

SCORING GUIDANCE:

EXAMPLES:

LAND RIGHTS

PROPERTY RIGHTS

Security Index

RATIONALE:
When rights are insecure, farmers may be more exploitative with resources and land. Insecurity and uncertainty can arise from crime, civil unrest, war, government instability or the government's use of eminent domain.

METRIC:
Extent to which the government threatens to reduce or eliminate the property rights. NA if there is no property right.

5	Very Strong: Property rights are completely respected by the government
4	Strong: Rights are mostly respected by the government and generally survive changes in government administration
3	Moderate: Rights are at risk of retraction with changes in administration
2	Weak: Rights are highly threatened or there is high political uncertainty
1	None: Property rights are not protected

SCORING GUIDANCE:
In some cases, the leasor of land may be other farmers or nongovernmental entities and can present another source of insecurity and uncertainty that should count against the score.

EXAMPLES:

LAND RIGHTS

PROPERTY RIGHTS

Durability Index

RATIONALE:

Short-duration rights creates more exploitative behavior and can undermine investment and financing.

METRIC:

Duration of the property right. NA if there is no property right.

5	Very Strong: > 10 years to perpetuity
4	Strong: 6 to 10 years
3	Moderate: 1 to 5 years
2	Weak: Seasonal
1	None: None/daily

SCORING GUIDANCE AND EXAMPLES:

This metric should reflect the legal durability of the right. For example, if a shellfish farmer can obtain a submerged land lease lasting for 5 years without reapplication, then they should score a 3.

LAND RIGHTS

PROPERTY RIGHTS

Flexibility Index

RATIONALE:

When the timing of harvest, production technology and other decisions on farming practices are within the rightholders' control, decision-making should lead to more efficient outcomes. Low scores will reflect restrictions and regulations that force inefficiencies. Restrictions can be set by the state or government entity or by the property owner in the case that the access right is leased.

METRIC:

Ability of right holders to be flexible in use of the production area, timing and production technology employed. Low scores will reflect restrictions that force inefficiencies. NA if there is no property right.

5	Very Strong: All decisions on production practices and production area are in the rightholder’s control
4	Strong: Minimal restrictions on production practices and production area
3	Moderate: Modest restrictions on production practices and production area
2	Weak: Significant restrictions on production practices and production area
1	Production practices and production area are not in the owner’s control

SCORING GUIDANCE AND EXAMPLES:

LAND RIGHTS

PROPERTY RIGHTS

Exclusivity Index

RATIONALE:

Under strong property rights, all decisions and access to the property are controlled by the rightholder, otherwise, wealth creation can be undermined. This metric measures intrusion by outsiders who can directly affect production through theft, or indirectly by degrading land quality or infrastructure.

METRIC:

Ability of right holders to exclude those who do not have the right from affecting production or the market. NA if there is no property right.

5	Very Strong: All decisions and access to land are controlled by the rightholder
4	Strong: Little intrusion of those without rights
3	Moderate: Modest intrusion on land and use by those without rights
2	Weak: Significant intrusion on land by those without rights
1	None: Completely unrestricted open access, despite putative right

SCORING GUIDANCE:

EXAMPLE:

Co-Management

Collective Action

Proportion of Farmers in Industry Organizations

Rationale:
Co-management is increasingly being integrated into aquaculture development projects and represents a power- and cost-sharing partnership that can capitalize on knowledge and capacity of user groups and the government. In theory, the degree to which producers are organized into cooperatives or associations that can act collectively to influence management and coordinate business arrangements can contribute to more legitimate, sustainable and effective management systems.

Metric:
Proportion of production where the primary producers consider themselves to be members of organized associations. This captures whether the producers are organized to influence outcomes.

5	Virtually all
4	70-95%
3	35-70%%
2	5-35%
1	Virtually none

Scoring Guidance:
This metric measures whether or not farmers are involved in organizations that are capable of influencing management or coordinating business transactions. The next two scores indicate how effective these organizations are at influencing management or coordinating joint business arrangements.

Examples:

1. It is estimated that between 80 and 90% of catfish farms are part of the Catfish Farmers of America Association.

Co-Management
Collective Action

Farmer Organization Influence on Management

Rationale:
Producer organizations can influence management and access by directly managing access rights or by taking political action to influence regulatory policy and access. Such participatory management schemes may facilitate outcomes that improve wealth accumulation to producers. Producers possess specialized knowledge of their resources and may contribute effectively to management plans. In addition, improved compliance with regulations is believed to be a benefit of participatory management.

Metric:
Qualitative measure of how much influence producing organizations have, either directly or through political collective action, on management, regulation and access to land and water.

5	Farmer organizations effectively determine management and regulations
4	Farmer organizations have significant influence in determining management and regulations
3	Farmer organizations are politically active, but not controlling
2	Farmer organizations conduct social or informal monitoring and management
1	Farmer organizations make no active effort or have no capacity to influence management

Scoring Guidance:
This measures how effective farmer organizations are at influencing de facto management. It may be difficult to distinguish between associations that are focused on joint marketing or management collective action, but it should be possible to get an idea of the organizations' goals if direct meetings are arranged.

Examples:

CO-MANAGEMENT

COLLECTIVE ACTION

Farmer Organization Influence on Business & Marketing

RATIONALE:

Producer organizations can influence management and access by directly managing access rights or by taking political action to influence regulatory policy and access. Such participatory management schemes may facilitate outcomes that improve wealth accumulation to producers. Producers possess specialized knowledge of their resources and may contribute effectively to management plans. In addition, improved compliance with regulations is believed to be a benefit of participatory management.

METRIC:

Qualitative metric of how much influence producing organizations have, either directly or through political collective action, on business operations and marketing.

5	Farmer organizations cooperatively determine marketing and operational details
4	Extensive joint marketing
3	Large subgroups facilitating marketing; joint purchasing
2	Small subgroups cooperating in purchasing or operations
1	No active effort or capacity to influence business operations

SCORING GUIDANCE:

This measures how effective farmer organizations are at conducting joint business or marketing. It may be difficult to distinguish between associations that are focused on joint marketing or management collective action, but it should be possible to get an idea of the organizations' goals if direct meetings are arranged.

EXAMPLES:

1. The Norwegian Seafood Council is not an industry organization but rather a generic marketing organization. However, the NSC is paid for by industry fees and regularly seeks input from the salmon industry, thus scores a 5.

CO-MANAGEMENT

PARTICIPATION

Days in Stakeholder Meetings

RATIONALE:
This metric is a proxy for the efficiency of the management process and stakeholder participation. Stakeholder participation is a means to incorporate specialized knowledge into management, in addition to improving legitimacy and compliance to regulations. However, it may also increase management costs and time required to implement management.

METRIC:
Days in stakeholder meetings per year spent by a participant in the production sector who is active in management. Note these are days with meetings, not FTE days. Include federal meetings with public participation.

5	More than 24 per year
4	12-24
3	6-11
2	1-5
1	None

SCORING GUIDANCE:
This includes time in meetings of farmer organizations directed at management, as well as at private and public meetings of management bodies themselves. The meetings with management must provide an opportunity for stakeholder input, and not simply be public hearings viewable by stakeholders. The metric refers to stakeholders who are active in management, who probably attend more days than the average stakeholder.

EXAMPLES:

CO- MANAGEMENT
PARTICIPATION

Industry Financial Support for Management

RATIONALE:
If the industry pays for the costs of management, it is likely that efficiency will be improved and the concomitant control over management exerted by the industry will lead to improved outcomes for producers, especially wealth generation. Some researchers claim that user participation is a key determinant of whether a management system generates equity, resilience, efficiency and stewardship.

METRIC:
Proportion of management costs paid for by the producing or processing sectors. Fees for new licenses/permits and taxes on production should be counted.

5	Virtually all
4	50-95%
3	5-50%
2	1-5%
1	None

SCORING GUIDANCE:
In some cases, the industry supports research and/or management costs. This does not include money from development agencies or NGOs that are more closely aligned with government than the industry.

EXAMPLES:

CO- MANAGEMENT
COMMUNITY

Leadership

RATIONALE:
There is strong empirical and theoretical evidence that strong community leadership can alleviate common property dilemmas in fisheries and other natural resources (Gutierrez et al. 2011; Ostrom 1990). Researchers hypothesize that prominent community leaders are particularly important in situations where central governments have limited control and may be responsible for successfully managing resources and securing the livelihoods of communities depending on them.

METRIC:
Qualitative measure of whether the farming community has strong leadership capable of envisioning and implementing effective management (this role may be provided by processors). Bins 2 and 4 may be scored as midpoints between descriptions.

- 5 Widely recognized individual leader, or small group of individual leaders, who provides vision for management and is able to attract stakeholders to that vision
- 3 Ex officio leadership stations that maintain management institutions, but are not currently providing strong vision
- 1 No recognized leader providing vision for stakeholders

SCORING GUIDANCE:
Most industries with or without formal organizations have someone whose job it is to be a leader. This person may be the leader, but the individual may not hold a formal post. This metric captures the effectiveness of that leader at catalyzing change for the better.

EXAMPLES:

CO- MANAGEMENT
COMMUNITY

Social Cohesion

RATIONALE:
There is empirical and theoretical evidence that strong social cohesion can alleviate common property dilemmas by helping participants coordinate mutually beneficial solutions. Researchers hypothesize that social cohesion is particularly important in situations where central governments have limited control over regulations and may be responsible for successfully managing resources and securing the livelihoods of communities depending on them.

METRIC:
Measure of whether the producers are socially connected and interact regularly in farming and non-farming spheres. Score one point for each of the following:

- Common locations for gathering and meeting on a regular basis for non-farming business, culture or commerce
- Presence of shared social norms that facilitate transactional trust
- Presence of shared public institutions (government, schools, markets)
- Absence of differences in social status or caste that prevent interaction
- Absence of religious differences and/or conflict
- Absence of cultural, ethnic or tribal differences that obstruct interaction

5	6 points
4	5 points
3	3-4 points
2	1-2 points
1	0 points

SCORING GUIDANCE:
Please be sure to note the social cohesion attributes that were present within the FPI worksheet.

EXAMPLES:

CO- MANAGEMENT
GENDER

Business Management Influence

RATIONALE:
Women play important roles in fisheries, aquaculture and seafood value chains. Commercialization and privatization of small-scale production may lead to marginalization and exclusion of vulnerable groups of people including women. This metric will enable hypothesis testing related to gender equality and performance of the community within the production and post-production sectors.

METRIC:
Extent of women's influence (not just participation) in the management of farming and processing businesses, including decision-making, ownership and financing. This will not typically include development project staff or other "outsiders." Bins 2 and 4 may be scored as midpoints between descriptions.

5	Business management dominated by women
3	Business management is balanced between women and men
1	Business management dominated by men

EXAMPLES:
1. In the U.S. catfish industry, women and men play nearly equal roles in business operations of the production and processing sectors, thus scoring a 3.

CO- MANAGEMENT
GENDER

Resource Management Influence

RATIONALE:
Women play important roles in fisheries, aquaculture and seafood value chains. Commercialization and privatization of small-scale production may lead to marginalization and exclusion of women. This metric will enable hypothesis testing related to gender equality and performance of the community within the production and post-production sectors.

METRIC:
Extent of women's influence (not just participation) in the management, including scientific research and resource access decisions. This will not typically include development project staff or other "outsiders." Bins 2 and 4 may be scored as midpoints between descriptions.

5	Resource management dominated by women
3	Resource management is balanced between women and men
1	Resource management dominated by men

EXAMPLE:

CO- MANAGEMENT
GENDER

Labor Participation in Production Sector

RATIONALE:
Women play important roles in fisheries, aquaculture and seafood value chains. Commercialization and privatization of small-scale production may lead to marginalization and exclusion of women. This metric will enable hypothesis testing related to gender equality and performance of the community within the production and post-production sectors.

METRIC:
Proportion of those involved in the production sector labor pool, either as farmers or farm workers who are women.

5	80-100% are women
4	60-80% are women
3	40-60% are women
2	20-40% are women
1	Less than 20% are women

EXAMPLES:
1. The majority of U.S. catfish farmers and workers are men, and thus this metric scores a 1.

Co- Management
Gender

Labor Participation in Processing Sector

Rationale:
Women play important roles in fisheries, aquaculture and seafood value chains. Commercialization and privatization of small-scale production may lead to marginalization and exclusion of women. This metric will enable hypothesis testing related to gender equality and performance of the community within the production and post-production sectors.

Metric:
Proportion of those involved in the post-production labor pool, as buyers, sellers, managers or workers who are women.

5	80-100% are women
4	60-80% are women
3	40-60% are women
2	20-40% are women
1	Less than 20% are women

Examples:

MANAGEMENT

MANAGEMENT INPUTS

Management Expenditure to Farm-Gate Value

RATIONALE:
This is a measure of the cost of aquaculture management in proportion to the value of production. Efficiency in management is essential for ensuring that human well-being is properly aligned environmental and economic objectives.

METRIC:
Government, industry, and aid agency expenditures on management activities including research, enforcement, and management capacity development (but not infrastructure) relative to the farm-gate value of production.

5	Less than 5% of farm-gate value
4	5-25%
3	25-50%
2	50-100%
1	More than the farm-gate value

EXAMPLE:

MANAGEMENT

MANAGEMENT INPUTS

Enforcement Capability

RATIONALE:
There is empirical and theoretical evidence that poorly enforced management is ineffective at controlling environmental impacts, while efficiently using resources is critical to maintaining community well-being.

METRIC:
Qualitative measure of enforcement capacity. Enforcement capacity includes that of the government, fishing organization or any other group that can effectively enforce management.

5	Strong capacity to enforce regulations in all producing areas
4	Capacity to enforce regulations in most producing areas
3	Capacity to enforce in some producing areas, other areas have very limited capacity
2	Capacity to enforce only near major cities
1	No capacity to enforce

SCORING GUIDANCE:
Different industries, with different management systems and different opportunities to subvert rules, have different enforcement needs. Although the most common case is related to space, and thus described in the scoring metric, the scoring should be based on how effectively enforcement capacity meets enforcement needs.

EXAMPLES:

1. Despite being conducted in rural areas of the U.S. there is strong capacity to ensure enforcement of regulations on catfish farms, so this metric is scored a 5.

MANAGEMENT

MANAGEMENT INPUTS

Management Jurisdiction

RATIONALE:

It is hypothesized that industries operating under a single management jurisdiction or with a formal system for joint management will be more effectively and efficiently managed.

METRIC:

This metric measures the extent that the industry in within a single management jurisdiction and, if in multiple jurisdictions, the level of coordination between the management jurisdictions.

5	Industry cluster is within a single management jurisdiction, or multiple jurisdictions have an effective, formal system for joint management throughout the range
4	Effective coordinating institution facilitates joint management throughout the region of primary importance
3	There is a coordination structure, but it does not have binding authority
2	Informal institutions for coordinating management
1	Jurisdictions effectively manage the same industry independently

EXAMPLES:

MANAGEMENT

MANAGEMENT INPUTS

Generations Separated by Selective Breeding

RATIONALE:
Most economists agree that an advanced breeding program is essential to improve economically important traits in farmed organisms. The key objectives of most selective breeding programs are to domesticate the organism and to increase productivity and quality, making better use of feed, water and land resources. Some scientists argue that selective breeding programs can inadvertently do harm to wild fish populations when domesticated fish escape from farms, while others argue that the more domesticated the organism, or the more generations separated from the wild strain, the less probable that the fish will survive and reproduce in wild environments (Lorenzen et al. 2012).

METRIC:
Measures the number of generations the produced organism is separated from the wild strain.

5	10 or more generations
4	5-9 generations
3	2-4 generations
2	1 generation
1	No selective breeding

SCORING GUIDANCE:
The number of generations separated is likely to vary slightly by age of the breeding company. Score this measure based on the level of selective breeding for the majority of production value.

EXAMPLES:

1. In Norway, traits for growth, disease resistance and quality characteristics have been selected for through ~12 generations of selective breeding. Thus, scoring a 5 on this metric.

MANAGEMENT

MANAGEMENT INPUTS

Coordination of Regulatory Authorities

RATIONALE:
There is evidence that industries with weak regulatory oversight can have harmful effects on the environment, food safety and health of the community. On the other hand, industries regulated by multiple, uncoordinated authorities and agencies have failed to grow and prosper. Fragmented and complex regulatory systems can deter investors and developers due to high degrees of uncertainty and high start up costs.

METRIC:
Level of coordination between regulatory authorities within a region.

5	The regulatory system is efficiently coordinated
4	Regulatory oversight of multiple functions, but there are many agencies and costly or inefficient regulatory practices
3	Regulatory oversight of multiple functions, but regulatory inefficiency is a major impediment to business development
2	Regulatory oversight of only a few functions
1	No regulatory oversight

SCORING GUIDANCE:

MANAGEMENT

MANAGEMENT INPUTS

Level of Subsidies

RATIONALE:
Subsidies distort market pricing and may increase effort levels at the expense of resources. Lower subsidies are indicative of greater market efficiency.

METRIC:
Measures the use of subsidies in the industry. Score one point for each of the following key subsidy categories that are present:

- Fuel subsidies (not including reduced highways taxes)
- Insurance subsidies
- Capital or capital loan subsidies
- Price support (through inputs or direct payments).

5	No subsidies
4	1 subsidy category
3	2 subsidy categories
2	3 subsidy categories
1	4 subsidy categories

EXAMPLES:

MANAGEMENT

MANAGEMENT INPUTS

Percentage of Marine Ingredients

RATIONALE:
It is hypothesized that increased aquaculture production will increase demand for and exploitation of wild capture fisheries for fishmeal in aquafeeds and consequently threaten the sustainability of wild capture fisheries (Naylor et al. 2000; Deutsch et al. 2007). Production of herbivorous fish, shellfish and other organisms that do not rely on marine ingredients may be more environmentally friendly. A lower percentage of marine ingredients across similar industries or through time may be indicative of improved knowledge and innovation in aquafeeds and nutrition.

METRIC:
This measures the percentage of marine ingredients in aquafeeds including both fishmeal and fish oil.

5	Less than 1% of feed is marine ingredients
4	1-15%
3	16-30%
2	31-45%
1	More than 45% of feed is marine ingredients

SCORING GUIDANCE:
If the produced species do not receive direct feed inputs of fish meal or fish oil (e.g., clams), they should score a 5. Note, this score should reflect what is practiced by the industry, not based on the nutritional requirements of the species.

EXAMPLE:
1. U.S. catfish feeds are mainly plant-based, typically containing soybean meal, cottonseed meal, corn and wheat byproducts, so this metric scores a 5.
2. Clam farming in Cedar Key, Florida also scores a 5 because there are no feed inputs.
3. Typically between 15 and 25% of Atlantic salmon feeds are fishmeal and fish oil, thus receiving a score of 3.

MANAGEMENT

MANAGEMENT INPUTS

Traceability of Feed Inputs

RATIONALE:
Transparency in seafood supply chains has traditional received most attention with respect to issues of food safety. More recently, traceability in seafood is providing avenues to combat environmental, legal and human rights issues in seafood production. It is hypothesized that traceability of feed inputs is critical for environmentally and socially sustainable industries.

METRIC:
Extent that marine ingredients in aquafeeds are traceable throughout the supply chain.

5	All marine ingredients are fully traceable
4	Three-quarters of marine ingredients are traceable
3	Half of marine ingredients are traceable
2	A quarter of marine ingredients are traceable
1	Virtually none of the marine ingredients are traceable

SCORING GUIDANCE:

MANAGEMENT

MANAGEMENT INPUTS

R&D

RATIONALE:

R&D is essential for productivity enhancing innovations that can decrease production costs and consumer prices and increase the number of product forms. In salmon aquaculture, R&D has played a critical role in the evolution of feeds and disease management.

METRIC:

Ratio of R&D funding to farm-gate value of production.

5	4.0% or greater
4	2.5 - 3.9%
3	1.0 – 2.4%
2	Less than 1.0%
1	No R&D funding

SCORING GUIDANCE:

This will likely be a rough estimate, and it should consider both private and public R&D.

MANAGEMENT

MANAGEMENT INPUTS

Private R&D

RATIONALE:
Some argue that R&D for agricultural and aquacultural purposes should be conducted exclusively in public institutions. However, it is more probable that a mix of public and private R&D will lead to more sustainable resource use and wealth generation.

METRIC:
Extent that R&D is conducted by the private sector as opposed to universities and public research stations.

5	Virtually all research is conducted by the private sector
4	Most research is conducted by the private sector, limited research at universities and public research stations
3	Equal shares of private and public research
2	Most research is conducted by the universities and public research stations, limited research at by the private sector
1	Virtually all research is conducted at universities and public research stations

EXAMPLES:
1. Most catfish research is conducted by universities, university extension, and public research stations like the USDA, although some research does come out of the private sector. Thus this metric scores a 2.

Management
Data

Biological Data Collection

Rationale:
Most researchers agree that a successful aquaculture industry requires a high degree of knowledge on the organism's biology, nutrition, reproduction and disease to effectively control the production process and the process of obtaining that information may facilitate cooperation amongst industry stakeholders.

Metric:
Extent that biological data is available for the organism. This includes knowledge on the species' biology, growth, reproduction, physiology and disease.

5	Highly advanced data collection on species' biology, growth patterns, reproduction, physiology and disease
4	Good data collection on species' biology, growth patterns, reproduction, physiology and disease
3	Adequate knowledge of species' biology, growth patterns, reproduction, physiology and disease
2	Limited knowledge of species' biology, growth patterns, reproduction, physiology and disease
1	Virtually no knowledge of species' biology, growth patterns, reproduction, physiology and disease

Examples:

Management
Data

Market and Economic Data

RATIONALE:
Most researchers agree that an industry will be more effective in achieving social and economic goals if data is collected to evaluate policy and management changes. It is hypothesized that provision of timely, third-party data will improve management and wealth generation of participants.

METRIC:
Extent that economic data is available. Price, trade, input factor use and production costs are examples of economic data.

5	Consistently collected economic data are available - third party provision of timely data
4	Consistently collected economic data are available
3	Limited reliable economic data collected
2	Available data based on small sample sizes
1	No economic data is centrally collected

EXAMPLES:

MANAGEMENT

MANAGEMENT METHODS

Regional Disease Control

RATIONALE:
Evidence suggests that disease has been one of the most challenging issues facing global aquaculture and one of the largest sources of economic loss. This metric will allow for testing whether biosecurity/fish health plans and a strong veterinary service are effective at reducing losses and supporting wealth generation.

METRIC:
This metric measures the extent to which an effective regional biosecurity and veterinary service exists.

5	Effective regional biosecurity/animal health plan and regional veterinary service exist with power to force abandonment of production
4	Regional biosecurity/animal health plan and regional veterinary service exists but does not have power to force abandonment of production
3	Regional biosecurity/animal health plan exists, but a reliable regional veterinary service doesn't exist
2	Regional biosecurity/animal health plan exists, but it is not consistently implemented or lacks a strong scientific basis
1	No regional governance of biosecurity and animal health exists

EXAMPLES:

MANAGEMENT

MANAGEMENT METHODS

Genetic Management

RATIONALE:
Some researchers argue that only organisms native to the region, or non-reproducing organisms, should be farmed to minimize the genetic and ecological impacts of aquaculture on natural ecosystems. Others argue that culture of an organism outside its natural range may prevent detrimental effects on the ecosystem given it will likely be so unfit in the environmental that it will be unable to survive and reproduce (Lorenzen et al. 2012).

METRIC:
Measure of whether the produced species is native to the region and is genetically modified. Note this should measure what is actually practiced by the industry, and reflects how well management can regulate the industry.

5	Native species, triploidy or reduced fertility through temperature or pressure shock
4	Native species, reproducing
3	Genetically modified, non-transgenic
2	Genetically modified, transgenic
1	Introduced species

SCORING GUIDANCE:
The production of triploid organisms using pressure or temperature shock is not considered genetic modification, and is scored a 5 as this process reduces fertility and potential impacts of escapees interbreeding with wild populations. Non-transgenic genetic modified organisms are defined as organisms where the genome was modified but another species' genes was not introduced into the genome (e.g. the gene for browning can be silenced in apples). Transgenic organisms are most commonly referred to by the use of GMOs and result from a process where the organism's genome is changed by the addition of a gene from another species.

Management

Management Methods

Discharge/Effluent Control

RATIONALE:
There is increasing use of economic instruments for managing interactions between aquaculture and the environment. Perhaps, the most commonly used approach is use of pollution fees or taxes which influences polluting behavior. Market-based permitting systems are also becoming more popular for managing emissions of greenhouse gases and have been proposed to manage nutrient discharge from aquaculture farms. Non-economic approaches to management are command and control approaches such as restrictions on water use, feed limits and production moratoriums.

METRIC:
Measures the use of economic instruments in effluent/discharge management.

5	Regional effluent management using market-based permit systems
4	Regional effluent management using pollution fees or taxes
3	Regional effluent management using command and control approaches
2	Regional effluent management using command and control approaches, but compliance is weak
1	No regional effluent management

SCORING GUIDANCE:

MANAGEMENT
MANAGEMENT METHODS

Antibiotic Use

RATIONALE:
Intensification and high densities increase the likelihood of pathogen outbreaks in animal production systems which can negatively impact production rates and quality. In some cases, this has created a reliance on antibiotics and some prophylactic use of antibiotics especially in countries where regulatory limits and monitoring are not adequate. However, antibiotic usage is highly variable. For instance, Norwegian salmon production would score a 5 as antibiotic use has dramatically declined to less than 1 g/mt in recent decades, whereas Chilean salmon production would score a 1 as antibiotic use is estimated between 600 and 700g/mt.

METRIC:
Measures the level of antibiotic use using two scales; a quantitative scale in grams per mt of production and a qualitative scale when data are not available.

5	No antibiotic use or use is among the lowest in animal protein production; less than 1 g/mt of harvested product
4	Antibiotic use is low; between 1-10 g/mt of product
3	Antibiotic use is moderate; 10-100 g/mt of product
2	Antibiotic use is high; 100-400 g/mt of product
1	Antibiotic use is very high and among the highest in animal protein production; more than 400 g/mt of product

EXAMPLES:
1. Antibiotics are only used in very small quantities in U.S. catfish fingerling production. They are not used during growout. Thus, scoring a 5 on this metric.

MANAGEMENT

MANAGEMENT METHODS

Antibiotic Use Practices

RATIONALE:

Intensification and high densities increase the likelihood of pathogen outbreaks in animal production systems which can negatively impact production rates and quality. In some cases, this has created a reliance on antibiotics and some prophylactic use of antibiotics especially in countries where regulatory limits and monitoring are not adequate. More so, antibiotics are being used on a continuous basis as a growth stimulus and such practices are considered harmful in their potential to increase antibiotic resistance.

METRIC:

A measure of the circumstances under which antibiotics are used: as a treatment for disease, disease prevention (i.e., a prophylactic) and as a growth stimulus.

5	No antibiotic use
4	Antibiotics used for limited time periods to treat identified disease
3	Antibiotics used for treating disease and used infrequently as a prophylactic
2	Antibiotics used for treating disease and used frequently as a prophylactic
1	Continuous use of antibiotics as a prophylactic or as a growth stimulus

SCORING GUIDANCE:

MANAGEMENT
MANAGEMENT METHODS

Food Safety Services

RATIONALE:
Food safety services encompasses managing a wide range of chemical, biological and other emerging agents including antibiotics, antifungals and agrochemicals found in farmed organisms.

METRIC:
Measures the extent to which an effective food safety service exists.

5	Effective regional agency responsible for monitoring and regulating quality and safety standards, and inputs used in production according to US and EU standards
4	Effective regional agency responsible for monitoring and regulating quality and safety standards, and inputs used in production, but does not fully meet US and EU standards
3	Regional agency responsible for food safety exists, but only considers part of the value chain and/or monitoring is not sufficient
2	Regional agency responsible for food safety exists, but is ineffective and monitoring is low
1	No regional food safety services

EXAMPLES:
1. The U.S. catfish industry is regulated and monitored by a federal agency - the USDA Food Safety and Inspection Service, thus scoring a 5.

Management

Management Methods

Animal Welfare/Handling Practices

RATIONALE:
It is hypothesized that better animal welfare and handling practices will minimize stress, disease susceptibility, and as a result, will improve fish survival, product quality and wealth generation.

METRIC:
Measures adherence to animal welfare and handling standards.

5	Effective regional agency responsible for monitoring and regulating animal welfare standards, and handling practices according to US and EU guidelines
4	Effective regional agency responsible for monitoring and regulating animal welfare standards, and handling practices, but does not fully meet US and EU guidelines
3	Regional agency responsible for animal welfare exists, but is inconsistently enforced
2	Regional agency responsible for animal welfare exists, but is not at all enforced
1	No regional animal welfare/handling services

SCORING GUIDANCE:
Aquaculture sectors that are accountable to international animal welfare guidelines (e.g., OIE) should score a 5.

MANAGEMENT

MANAGEMENT METHODS

Damage Compensation/Management

RATIONALE:
Damage compensation programs can provide stability to farmers' income by managing risk and minimizing economic effects of crop loss caused by natural disasters and hazards. Although some argue these mechanisms can become alternative methods to transferring subsidies.

METRIC:
Measure of the mechanism by which primary production risk is covered. This is referring to protection of the biomass against production shocks.

5	Commercial insurance
4	Government provided insurance
3	Government handouts after reporting
2	Ad hoc handouts after severe crisis
1	None

SCORING GUIDANCE:
In the context of contract farming, if the contractor has ownership of the product and therefore holds the risk, this should reflect the damage compensation the contractor has access to.

EXAMPLES:

1. Very few catfish farms participate in crop insurance programs since they are capped by revenue. Farmers only receive government support following severe crises, thus scoring a 2.

MANAGEMENT

MANAGEMENT METHODS

Access to Water

RATIONALE:
Secure access to water of sufficient quality is important for successful aquaculture. In some systems, access to quality water can be simple, while in others, access to water is more complicated, highly governed and contentious, and can severely restrict production.

METRIC:
The extent that access to water influences production.

5	Strong access to water resources and all decisions on use are within rightholder's control
4	Access to water resources is good and causes few interruptions to production
3	Occasional water shortages influence production
2	Poor access to water poses risk and uncertainty in production
1	Water availability strongly influences production process

SCORING GUIDANCE:
Offshore cage farming will typically score a 4 as they usually have good access to water but have limited control of water resources.

EXAMPLES:
1. U.S. catfish farmers have good access to water resources and have strong control of decisions pertaining to water use, thus scoring a 5.

MANAGEMENT

MANAGEMENT METHODS

Land or Water Zoning/Management

RATIONALE:
Problems can arise due to the lack of spatial planning of aquaculture development including the spatial planning that doesn't consider social and economic objectives. These include disease outbreaks, productivity issues, social conflicts and lost benefits of farm clusters.

METRIC:
Measures the extent to which aquaculture development is spatially planned and managed.

5	Effective regional planning of aquaculture development that considers the environmental, economic and social objectives of development
4	Regional planning of aquaculture development that considers some objectives of development but not all
3	Regional planning of aquaculture development that considers only one objective of development
2	Regional planning of aquaculture development that is ineffective or is not followed
1	No regional plans for aquaculture zoning; spatially unplanned aquaculture development

SCORING GUIDANCE:

SUPPLY CHAIN

MARKETS & MARKET INSTITUTIONS

Transparency of Farm-Gate Price

RATIONALE:
Fair and efficient price discovery systems are essential for efficient production and wealth creation. The ability of producers to move among farm-gate buyers to those offering the best prices on a per-harvest basis.

METRIC:
Proportion of harvest sold in a transparent competitive pricing mechanism such as an auction or centralized farm-gate to wholesale market wherein sellers interact with many buyers and prices are public information.

5	Virtually all
4	70-95%
3	35-70%
2	5-35%
1	Virtually none

EXAMPLES:

1. Farmed salmon in Norway are not sold at auction, but prices are transparent. The Norwegian Seafood Council publishes weekly salmon prices online and are publicly available, thus this industry scores a 4.

SUPPLY CHAIN

MARKETS & MARKET INSTITUTIONS

Availability of Farm-Gate Price & Quantity Information

RATIONALE:

Market transparency is essential for efficient production and wealth creation. Market transparency is characterized by readily available, accurate price and quantity information. Fair and efficient price discovery systems are essential for efficient production schemes and wealth creation.

METRIC:

Scores the ability of the market to provide timely information to producers to which they can react by changing what or when they bring fish to market.

5	Complete, accurate price and quantity information available to market participants immediately
4	Reliable price and quantity information is available prior to the next market clearing
3	Price information is available but no timely quantity information
2	Price and quantity information are inaccurate, lagged or available to only a few
1	No information available

EXAMPLES:

1. Japan's Tsukiji market is probably the only example of a score of 5 in seafood markets. Complete quantity and farm-gate price information is immediately available to producers. Other examples of 5 would be the crude oil and corn markets in the U.S.

SUPPLY CHAIN

MARKETS & MARKET INSTITUTIONS

Number of Buyers

RATIONALE:
This metric is an indicator of relative market power. If the market is dominated by a single (or very few) buyers or sellers, price will favor the side with greater market power.

METRIC:
Typical number of buyers of farm-gate product accessible to a seller in a given market. If there are many production regions, this is the buyers per region. If producers are generally indentured to a single buyer through credit relationships, there is one buyer.

5	Highly competitive
4	4-6 buyers
3	2-3 competing buyers
2	A small number of coordinating buyers
1	There is one buyer

EXAMPLES:

SUPPLY CHAIN

MARKETS & MARKET INSTITUTIONS

Degree of Vertical Integration

RATIONALE:

Vertical integration facilitates the flow of information from the retailer to the production sector and tends to reduce transaction costs between market levels.

METRIC:

Proportion of production where the primary producer and primary processor/distributor are same firm. The role of vertical integration here is to ensure production and delivery of fish under a common management, increasing efficiency and reducing transactions costs.

5	Virtually all
4	70-95%
3	35-70%
2	5-35%
1	Virtually none

EXAMPLES:

1. About 40% of U.S. catfish is produced by vertically integrated companies where production and processing occur under one firm, so this scores a 3.

SUPPLY CHAIN

MARKETS & MARKET INSTITUTIONS

Level of Tariffs

RATIONALE:
Lower tariffs broaden the market, improve price discovery, and increase the opportunity to create wealth.

METRIC:
Official tariff rates charged for exports or imports to consumption markets.

5	Virtually none
4	0.5%-2.5%
3	2.5-5%
2	5%-10%
1	Over 10%

EXAMPLES:

SUPPLY CHAIN

MARKETS & MARKET INSTITUTIONS

Level of Non-Tariff Barriers

RATIONALE:

Lower non-tariff barriers broaden the market, improve price discovery, and increase the opportunity to create wealth.

METRIC:

Nontariff barriers include: quantity restrictions (import quotas), regulatory restrictions, investment restrictions, customs restrictions and direct government intervention.

5	Are not used to limit international trade
4	Have very limited impact on international trade
3	Act to impede some international trade
2	Act to impede a majority of potential international trade
1	Act to effectively impede a significant amount of international trade

EXAMPLES:

SUPPLY CHAIN
MARKETS & MARKET INSTITUTIONS

Contribution to Economy

RATIONALE:
Diversification of the economy is important for positive community outcomes and quality of life. High scores reflect the dependence of the community on the aquaculture sector.

METRIC:
This is the proportion of community jobs related to the sector (including both farming and processing jobs) and reflects the extent of diversification in the local economy.

5 Virtually all community members participate in jobs related to the sector
4 70-95%
3 35-70%
2 5-35%
1 Virtually none of the community members participate in the jobs related to the sector

SCORING GUIDANCE:

EXAMPLES:
1. The U.S. catfish industry operates in rural areas of the South, where employment alternatives are very limited and catfish production drives the local economy. Virtually all community members participate in jobs related to catfish farming, processing or in jobs related to providing inputs to the catfish industry. Thus this scored a 5.

SUPPLY CHAIN

INFRASTRUCTURE

International Shipping Service

RATIONALE:

In order to have access to a broader market, competitively priced international shipping is essential. Many argue that there are large economic returns from increased international trade and access to global markets.

METRIC:

The quality of the service available to access global high value markets, such as the US or EU (regardless of whether product currently exported). Average of the two measures (one for ocean shipping and another one for air shipping).

5	Ocean shipping services are readily available at lower than average rates
4	Ocean shipping services are readily available at average rates
3	Ocean shipping services are readily available at higher than average rates
2	Ocean shipping services are available but irregular
1	International shipping is not available at reasonable rates

5	Air shipping services are readily available at lower than average rates
4	Air shipping services are readily available at average rates
3	Air shipping services are readily available at higher than average rates
2	Air shipping services are available but irregular
1	International shipping is not available at reasonable rates

SCORING GUIDANCE:

Indicate the scores for ocean and air shipping in the FPI worksheet and provide the average score rounded to the nearest ones place.

EXAMPLES:

SUPPLY CHAIN
INFRASTRUCTURE

Road Quality Index

RATIONALE:
The quality of roads is directly related to the ability of firms to distribute their products, minimize transportation cost and create wealth.

METRIC:
Travel time-weighted average road quality between the fishery's primary port and the most practical export shipping port for exported product. For non-exported product measure road quality between the primary port and the major consumption center.

5	High-quality paved roads and extensive highways
4	Primarily paved two-lane roads and moderate highway
3	Primarily paved two-lane roads and minimal highway
2	Paved two-lane roads and well-graded gravel roads
1	Poorly maintained gravel or dirt roads

SCORING GUIDANCE:
It may be that value is not maximized by accessing the current primary market, but road quality prevents accessing the higher-value market.

This is an example of a 5 (paved and high speed): This is an example of a 1 (pitted and slow):

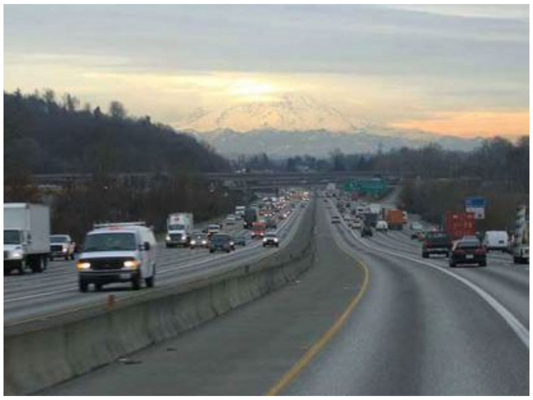

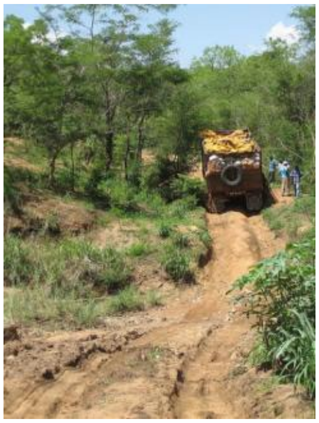

SUPPLY CHAIN
INFRASTRUCTURE

Technology Adoption in Production

RATIONALE:
The availability of the latest farming technology is important for firms to maintain global competitiveness and create wealth. Robotics and automation in aquaculture improves environmental control, reduces risk of catastrophic losses, can reduce production costs and improve product quality among other benefits.

METRIC:
Measures the extent that advanced technology is used in production. Examples of sophisticated technology includes automated feeding systems, automated water quality control, automated biomass and parasite monitoring, remotely operated vehicles, etc.

5	Adoption of sophisticated technology in production and monitoring
4	Some sophisticated technology is common, but not tall technology is available
3	Some use of sophisticated technology, but some technology is difficult to obtain
2	Most sophisticated technology is prohibitive
1	No sophisticated technology is used

SCORING GUIDANCE:
Examples of sophisticated technology includes automated feeding systems, automated water quality control, automated biomass and parasite monitoring, remotely operated vehicles, etc.

EXAMPLES:

SUPPLY CHAIN

INFRASTRUCTURE

Technology Adoption in Processing

RATIONALE:

The availability of the latest processing technology is important for firms to maintain global competitiveness and create wealth.

METRIC:

Average level of technology employed in the processing sector.

5	Adoption of sophisticated technology in processing; virtually all processing is performed by machine
4	Some sophisticated technology is common, but not all technology is available
3	Some use of sophisticated technology, but some technology is difficult to obtain
2	Most sophisticated technology is prohibitive
1	No sophisticated technology is used; virtually all processing is performed manually

SCORING GUIDANCE:

EXAMPLES:

SUPPLY CHAIN
INFRASTRUCTURE

Extension Service

RATIONALE:
Extension services are successful in many countries for transferring technology and information about best management practices, new technology, market conditions and regulatory changes. This information is often essential in a widely dispersed industry to help maximize returns and generate wealth. In the context of agriculture, there is some disagreement about whether the diffusion of information from extension services spreads easily among neighbors (Ryan and Gross, 1943) or whether the benefits from extension services accrue unequally and tend to benefit those least in need of assistance (Goss, 1979).

METRIC:
Degree to which government or NGOs help producers improve farming techniques or management through extension activities.

5	Broad extension service with field offices and close linkage with research community
4	Extension service with moderate field coverage and adequate linkage with the research community
3	Extension service, but with weak links to the research community
2	Minimal, poorly supported extension service
1	No extension service

EXAMPLES:
1. The U.S. catfish industry is supported by broad extension services that spans the entire production area. Extension services are tightly coupled with university research programs, thus scoring a 5.

SUPPLY CHAIN

INFRASTRUCTURE

Reliability of Utilities/Electricity

RATIONALE:
Reliable utilities are essential for firms to function efficiently and generate wealth. Reliable power sources reduce risk in production and in the supply chain.

METRIC:
Measurement of the source and reliability of electricity in the supply chain.

5 Reliable electrical grid provides power in sufficient quantity to prevent product loss
4 Supply chain relies on grid, but maintain backup generators
3 Supply chain relies on own generation capacity
2 Supply chain sometimes loses product due to condition or irregular fuel supply for generators
1 Reliable generators or fuel supply not available

SCORING GUIDANCE:
Note that this should be scored with a global context with respect to reliable electricity. It is important to ask about backup plans and recent losses in product value.

EXAMPLES:
1. Farmers in the U.S. catfish industry rely on the public electrical utilities but maintain backup generators to prevent product loss, thus scoring a 4.

SUPPLY CHAIN

INFRASTRUCTURE

Access to Ice & Refrigeration

RATIONALE:

Ice or refrigeration is essential for quality control and broadening the market. Without access to refrigeration technologies seafood products rapidly deteriorate and market access and timing of delivery are restricted thus hindering wealth generation.

METRIC:

The extent that ice and refrigeration is available, regardless of whether or not it is used.

5 Ice is available in various forms and in sufficient capacity to support fresh icing of all fish that needs to be iced
4 Ice is available in various forms, but quantity limits prevent applying to entire catch throughout supply chain
3 Ice is available in limited form and quantity, and thus applied only to most valuable portions of catch
2 Ice is available but capacity constrained; ice often reused, or used through melting stage
1 Ice quantities are extremely limited

EXAMPLES:

PRODUCTION
PRODUCER CHARACTERISTICS

Scale of Farm

RATIONALE:
Economies of scale may be important for wealth generation. Large scale enterprises may benefit from reduced cost per unit production, improved access to capital and better technology. However, some argue that small scale enterprises are more environmentally friendly, can cater to niche markets and can reduce risk by cultivating complementary species.

METRIC:
This measures the scale of the producer or farm in the industry.

5 Large scale, multi-plant, multi-national enterprise
4 Large scale, multi-plant enterprise
3 Medium, multi-plant enterprise
2 Family-run commercial enterprise
1 Primarily family-run subsistence enterprise

SCORING GUIDANCE:
Many industries are comprised of small and large-scale companies. This should measure should reflect the scale of the farms that contribute to the majority of production value. In the case of contract farming, this is not a measure of the contractor but rather the farm operator.

EXAMPLES:
1. The majority of U.S. catfish is produced on farms that are large-scale and part of a multi-farm enterprise, thus scoring a 4.

PRODUCTION

PRODUCER CHARACTERISTICS

Integrated Culture

RATIONALE:
Integrated multi-trophic aquaculture is an increasingly cited approach to improving the environmental problems associated with aquaculture (namely waste). IMTA emphasizes an ecosystem approach by farming fed species such as finfish in close proximity to species such as shellfish, seaweed and suspension feeders that extract the nutrient byproducts from the water column. Inclusion of this metric will allow hypothesis testing on the environmental and economic implications of IMTA compared to other forms of aquaculture.

METRIC:
The degree of polyculture used in the production systems.

5 Integrated multi-trophic culture
4 Aquaponics
3 Polyculture of 3 or more fish species
2 Polyculture of 2 fish species
1 Monoculture

SCORING GUIDANCE:

EXAMPLES:
1. U.S. catfish are produced alone in split ponds. No other species are produced simultaneously, thus scoring a 1.

PRODUCTION

PRODUCER CHARACTERISTICS

Proportion Under Contract Farming

RATIONALE:

Contract farming is commonly promoted as part of agricultural development policy. The literature suggests that participation in contract farming leads to improved income and food security benefits (Bellemare 2012). Contract farming in context of aquaculture is understudied and inclusion of this metric will allow hypothesis testing on the effects of contract farming on welfare.

METRIC:

This is the proportion of production produced under contracts.

5 Virtually all
4 70-95%
3 35-70%
2 5-35%
1 Virtually none

SCORING GUIDANCE:

Production under contracts includes marketing contracts, where the contractor involvement in production is minimal and contracts set a price, quantity and quality and delivery schedule, and production contracts where contractor involvement in production in high typically through ownership of the commodity and/ or responsibility for inputs and services such as feeds, fingerlings, and veterinary services.

EXAMPLES:

1. The exact proportion of production produced under contracts in the U.S. catfish industry is unknown, but the scorer was largely certain that it is between 5 and 35%, and thus it received a score of 2.

Case Studies: Norwegian Atlantic Salmon and Florida Cedar Key Clams

Profile: Atlantic Salmon, Norway (2019)

PRODUCTION SYSTEM

- The industry produces two very similar species, Atlantic salmon (about 95% and rainbow trout also known as salmon trout. The industry is served by two breeding companies that handle proprietary strains for different companies, a limited number of brood stock producers who deliver fertilized eggs to smolt plants. These are hatched and then kept in fresh water tanks at the smolt plant between 6 and 12 months before they are transferred to sea locations. The fish are then kept in sea pens for 14-20 months before being transported to a harvesting plant by a well boat. Each location has 6 to 12 netpens with a production between 1200 and 7000 mt. Feed is by far the most important input with a cost share of a bit over 50%.
- A breeding program was instituted by the fish farmers organization in the early 1970s and is still in operation. Originally the focus of the breeding program was growth rate, but today most program has between two and four factor one is selecting for primarily related to disease resistance. The main fish health issue is sea or salmon lice, although a number of diseases are common but generally well handled by treatment, prevention r forced harvesting.

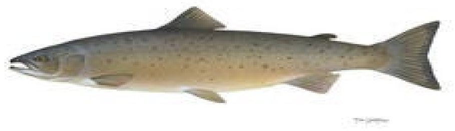

- The industry produces about 1.3 million mt of Atlantic salmon and 70,000 mt salmon trout at a total value of $9.2 bill.
- There are about 100 production companies where the smallest produces about 1,200 mt while the largest produces more than 200,000 mt. The average company produces about 14,000 mt.
- A license that allow 780 mt of biomass to be held cost about $10 mill and allow an annual production of about 1,200 mt. Net pens, feeding barges for two locations to harvest every year will be about $2mill in total

MANAGEMENT SYSTEM

- With one exception, all regulations are national, but enforced by local offices. The two most important are the directorate of fisheries who handle production and the food safety authority who handle diseases. Licenses are awarded by the national government, but the locations are awarded by the municipality where the plant is to be located, and they can refuse, forcing the company to take the license elsewhere.
- Most regulations are based on a single law on aquaculture production. However, the industry also have to fallow general rules with pollution and animal welfare as the most important.
- The producers lease water column from the government. In 2020 a production fee was introduced, but until then there were no annual expences. From 2002, a farmer has had to pay for new licenses. The rights are relatively weak de jure, but so far no leases has been voided except by no-use, and a lease that is not used for 5 years are forfeited.
- Compliance, monitoring and enforcement is quite good

Markets and Product Forms

- 284 exporters ship the main product, whole fresh salmon to 102 destinations in the period 2004-2004. The second most important product, fresh salmon fillets is shipped by 221 exporters to 84 destinations. The market is global, but in most years between 60% and 70% goes to the EU. All is for human consumption and more than 95% of production is exported.
- The main product by far is whole fresh salmon that is further processed closer to the consumer market to product forms that are still fresh never frozen. In total, about 90% of the exports are fresh, while most of the reminder is frozen. Most of the largest producers have exports fully integrated to the company and some also have subsidiaries in several of the larger markets, while there are also some large independent exporters as well as a large number of small and at times more specialized exporters. For some of the large companies long-term contracts are important, in particular in relation to large buyers who often by directly, while smaller exporters do not use contracts but can hedge with futures contracts, and tend to serve more complex supply chains where the fish change owner a number of times. Secondary processing often takes place in the country where the fish eventually is consumed, although in particular Poland act as a processing hub for central Europe with a focus on Germany.
- There are many potential buyers at all levels and the market is highly competitive
- A single farm produce between 1,200 mt and 7,000 mt, but a company may own many farms. The harvesting and primary processing plants are increasing in size. Many handles about 50,000 mt, some are even larger, and the smallest handles around 20,000 mt. Most companies are pure salmon (and salmon trout) companies

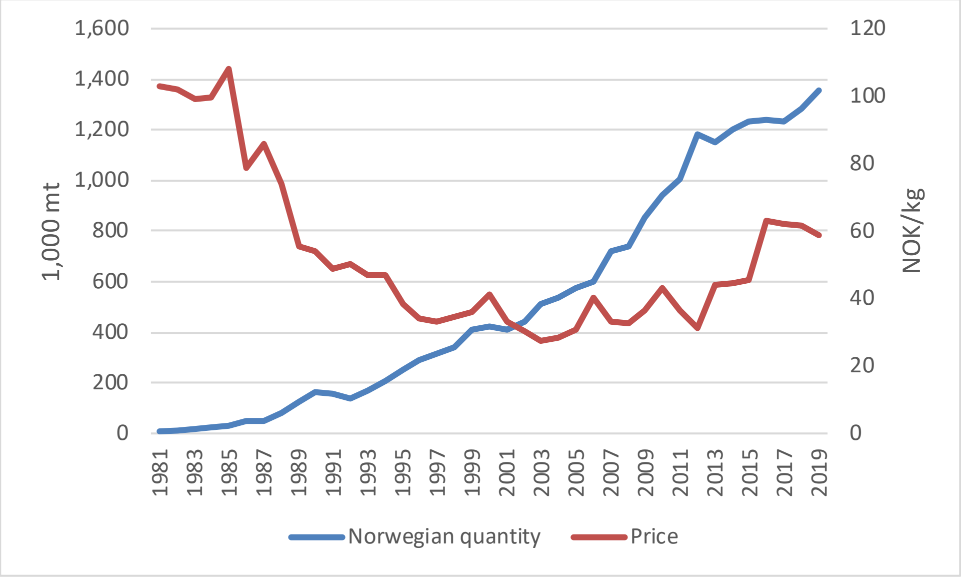

Supplementary Figure 1. Norwegian farmed salmon production and price.

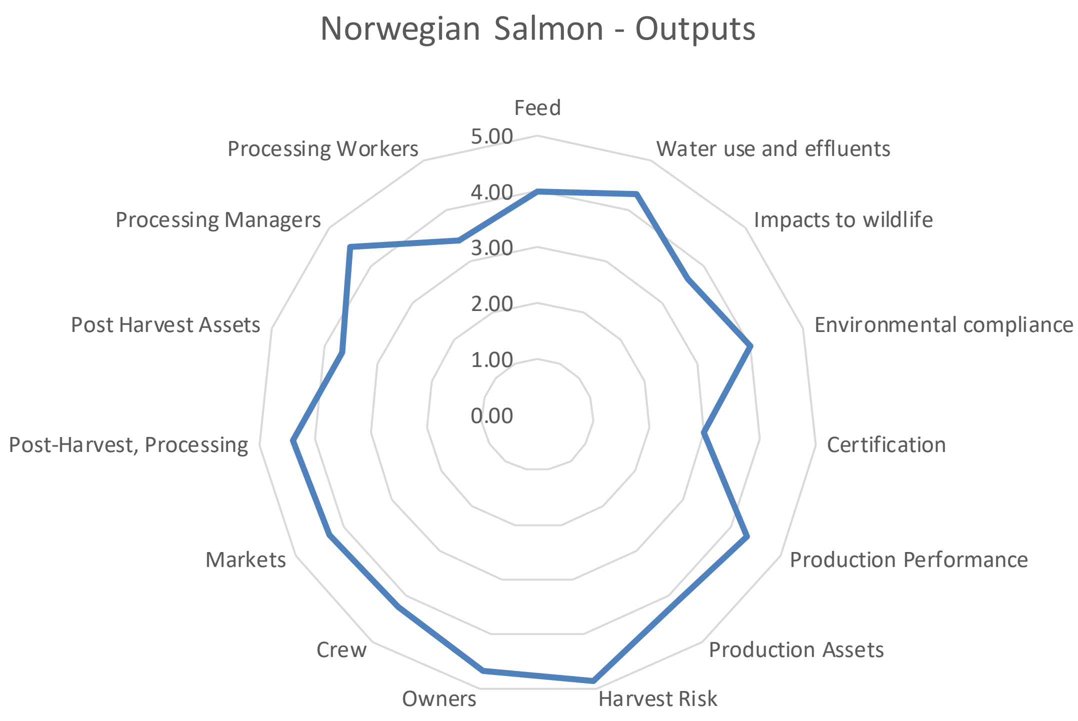

Supplementary Figure 2. Output scores for Norwegian Atlantic salmon, 2019.

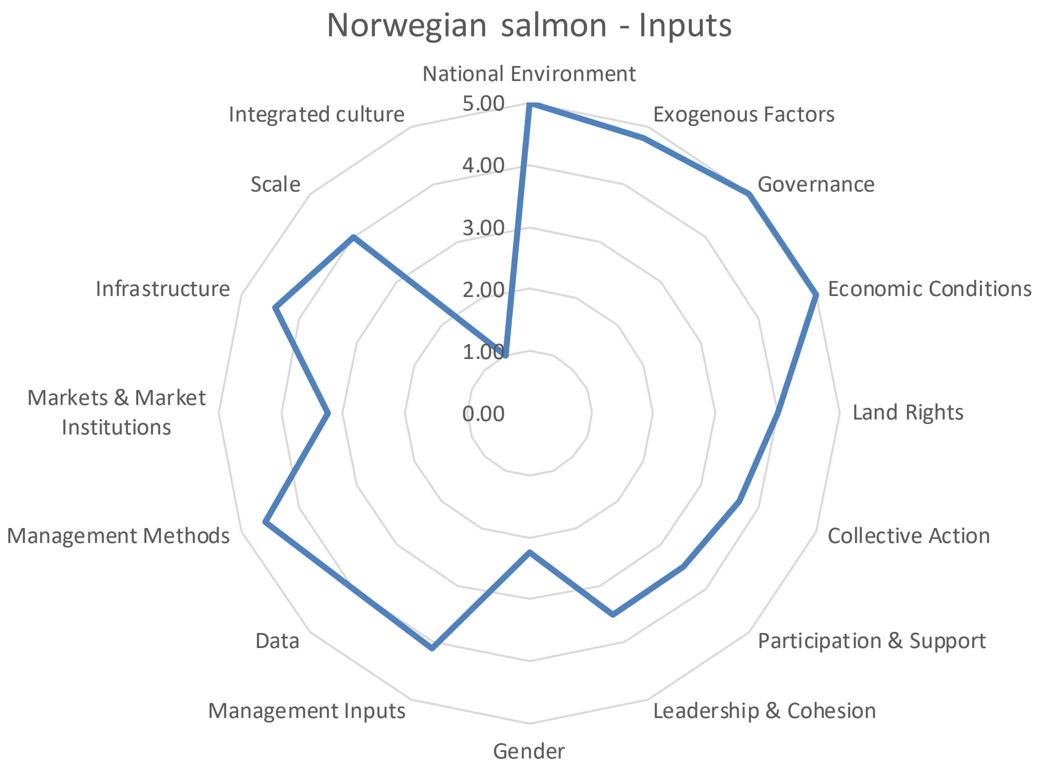

Supplementary Figure 3. Input scores for Norwegian Atlantic salmon, 2019.

Profile: Cedar Key Clams, Florida, USA (2019)

Production System

- The focus of this assessment is culture of the northern hard clam *Mercenaria mercenaria* in the Gulf of Mexico of Florida. Most production occurs in Cedar Key. In 2018, 95 clam operations reported sales totaling $14.3 million across 12 counties in Florida. The industry has had strong growth in the last decade.
- There are three stages of production: hatchery, nursery and growout. There are about 8 hatchery operations producing nearly half a billion seed annually. Temperature manipulation is used in the hatcheries to induce spawning. Egg and larvae are reared under controlled conditions in large tanks and fed cultured marine phytoplankton/microalgae for 10-14 days. The larvae settle out of the water column and are maintained in downwellers for another 30-45 days until approximately 1mm. The clam seed is then planted in nursery systems which are typically raceways or floating upwellers. There are about 40 nursery operations in Florida. In the nursery, seawater is pumped over the seed mass providing phytoplankton and oxygen. It takes about 6-12 weeks for the seed to reach 5-6 mm which is the minimum size for planting for growout. There are about 250 growout operations and 1,500 acres of state-owned submerged land off of 12 counties being used for growout. Seed is planted year round and thus clams can be harvested year round. Most growers use polyester mesh bag for growout. About 15,000 seed are planted into 4ftx4ft bags. Bags are typically staked into the bottom using PVC pipe and are typically planted in units or rows. After 3-6 months, seed of about 12-15mm are transferred to bags of larger mesh sizes at a density of 50-85/sq ft. Clams are grown for 12-18 months and harvested when they reach market size of ~1 inch. Many farmers use nets and screens to reduce predation. A winch or roller rig is used to harvest the bags since they become buried in the bottom sediments.

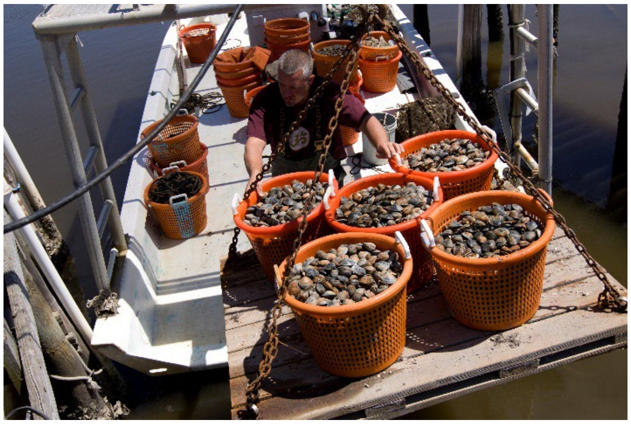

- The industry has no feed or chemical inputs. Broodstock are the offspring of wild New England stocks. There is some selection for shell type/color.
- Annual profits for clam farmers are estimated around $30-35,000.

Management System

- In Florida, state statutes established in the 1980s provide authority for leasing sovereign submerged lands. The Florida Department of Agriculture and Consumer Services (FDACS) Division of Aquaculture is responsible for the state of Florida's permitting and leasing program. Leases have a 10-year term, are renewable and transferable. There is a minimum of 100,000 clam seed must be planted per acre per year. Most leases are 2-4

acres. No mechanical harvest is permitted. Farmers must have harvested product at the processor within a certain time period (~10 hours) following harvest depending on season.

MARKETS AND PRODUCT FORMS

- Clams are rinsed, graded by size and sold live. The primary market is for littleneck clams (1inch). Average wholesale price is $0.12-$0.17 per clam. More than 75% of production is shipped live outside of the local area by about 20 wholesalers.

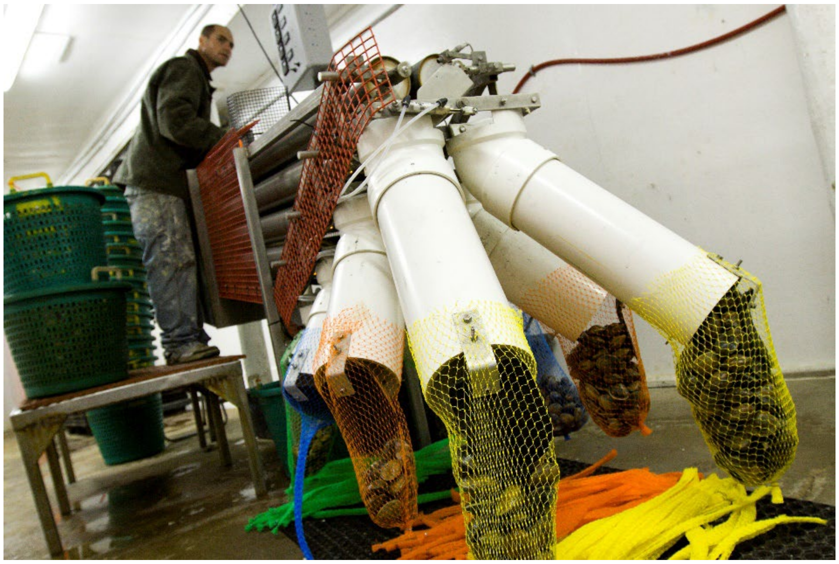

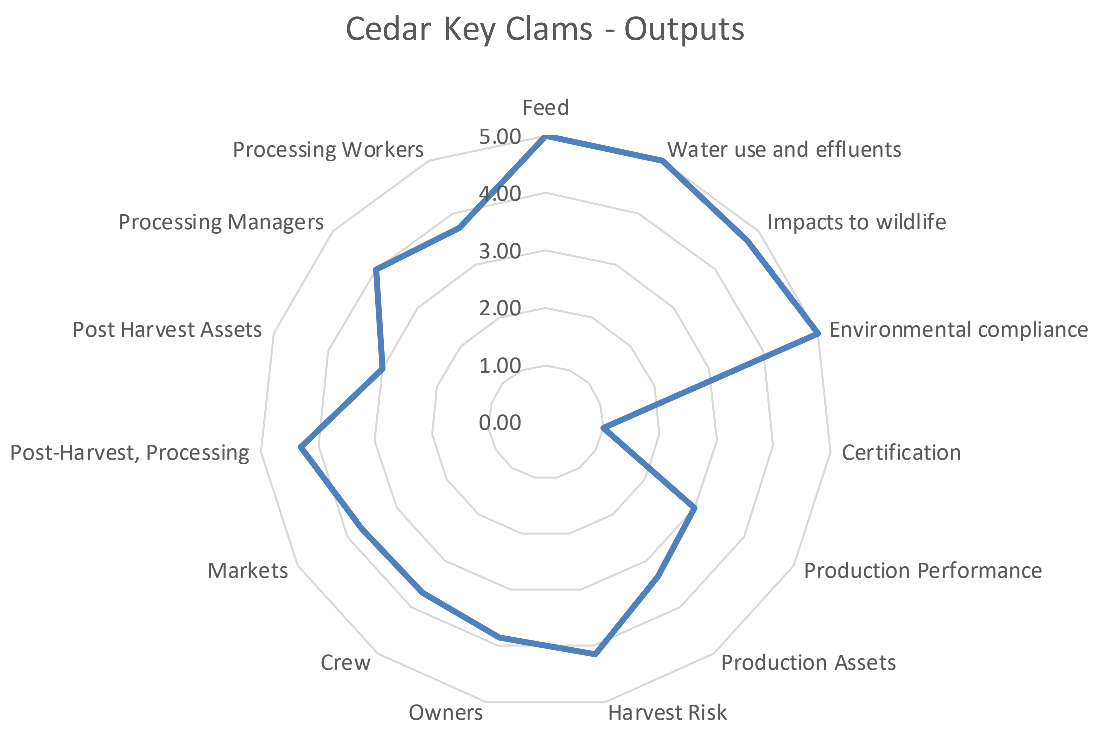

Supplementary Figure 4. Output scores for Cedar Key clams, 2019.

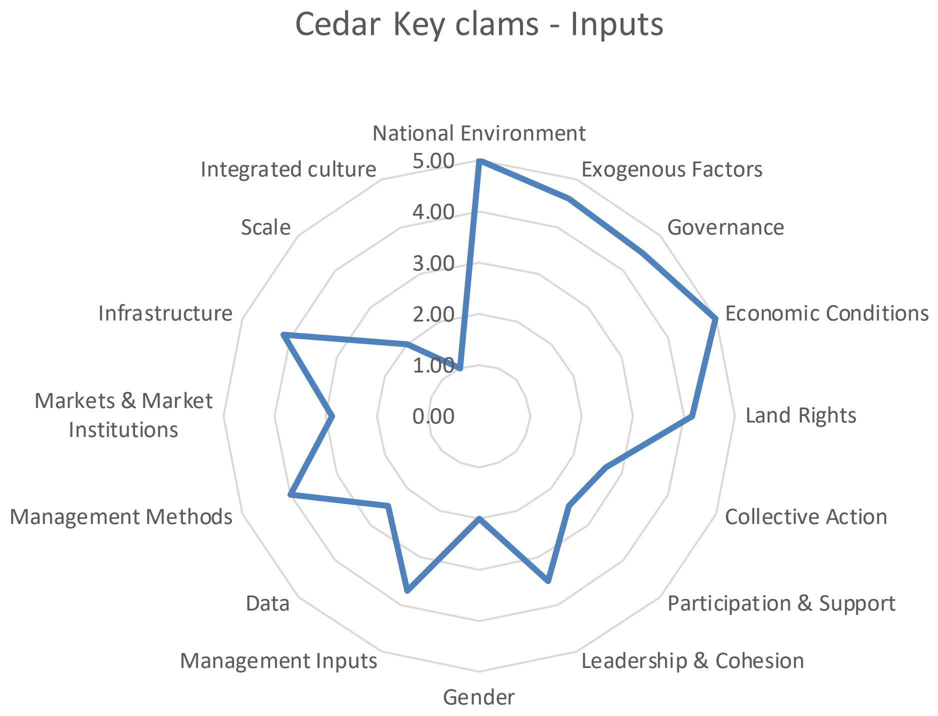

Supplementary Figure 5. Input scores for Cedar Key clams, 2019.

Supplementary Table 3. Aquaculture Performance Indicators – Outputs

Dimension	Component	Metric	Norwegian Atlantic salmon		Florida Cedar Key clams	
Environmental Health	Feed-related impacts	Sustainability of aquatic feed sources	4		5	
		Sustainability of non-marine feed ingredients	4	4.00	5	5.00
	Water use and effluents	Impact of discharge (nutrient emissions)	4		5	
		Non-nutrient emissions	4		5	
		Freshwater use	5	4.33	5	5.00
	Impacts to wildlife	Wildlife mortality	4		5	
		Benefits to wildlife	1		4	
		Ecological impacts of escaped fish	4		5	
		Genetic impacts of escaped fish	4		4	
		Parasite and disease transmission	2		5	
		Site use	5		5	
		Land Use	4		5	
		GHG emissions	5	3.63	5	4.75
	Environmental compliance	Compliance with environmental law	4	4.00	5	5.00
	Certification	Proportion of production with 3rd party certification	3	3.00	1	1.00
Production Sector	Production Performance	Production Technology	5		3	
		Adult feed	4		1	
		Juvenile survival rate	5		2	
		Juvenile production	5		3	
		Selective breeding and production time	5		1	
		Survival trend	4		4	
		Survival rate	4		2	
		Proportion of production affected by disease and parasites	4		4	
		Proportion of production affected by predation	5		4	
		Proportion of production that escapes	5		5	
		Proportion of production lost to handling and unspecified loss	4		5	
		Production costs compared to historic low	2	4.33	2	3.00
	Production Assets	Ratio of Asset Value to Gross Earnings	3		1	
		Total Revenue Compared to Historic High	5		5	
		Asset (Permit, Quota, etc...) Value Compared to Historic High	5		5	
		Borrowing Rate Compared to Risk-free Rate	3		3	
		Source of Capital	4		3	
		Functionality of Production Capital	5	4.17	3	3.33
	Risk	Annual Total Revenue Volatility	5		5	
		Annual Production Volatility	5		5	
		Intra-annual Production Volatility	5		3	
		Annual Price Volatility	4		5	
		Intra-annual Price Volatility	5		3	
		Spatial Price Volatility	5		4	
		Contestability & Legal Challenges	5	4.86	4	4.14
	Farm Owners	Earnings Compared to Regional Average Earnings	5		3	
		Owner Wages Compared to Non-Aquaculture Wages	5		2	
		Education Access	5		5	
		Access to Health Care	5		5	
		Social Standing of Farm Owners	5		3	
		Proportion of Nonresident Owners	3	4.67	5	3.83

Supplementary Table 3 (continued). Aquaculture Performance Indicators – Outputs

Dimension	Component	Metric	Norwegian Atlantic salmon		Florida Cedar Key clams	
Production Sector	Farm Workers	Earnings Compared to Regional Average Earnings	4		2	
		Worker Wages Compared to Non-Aquaculture Wages	4		3	
		Education Access	5		5	
		Access to Health Care	5		5	
		Social Standing of Workers	3		2	
		Proportion of Nonresident Workers	4		5	
		Worker Experience	4		3	
		Age Structure of Workers	4		5	
		Proportion of income spent on food	5	4.22	3	3.67
Post-Harvest Sector	Markets	Farm-gate Price Compared to Historic High	5		5	
		Final Market Use	4		4	
		International Trade	5		1	
		Final Market Wealth	5		5	
		Wholesale Price Compared to Similar Products	3		3	
		Capacity of Firms to Export to the US & EU	5		5	
		Farm-gate to Wholesale Marketing Margins	3		3	
		Food safety	4	4.29	4	3.71
	Supply Chain Performance	Processing Yield	5		5	
		Shrink	5		4	
		Capacity Utilization Rate	4		3	
		Product Improvement	5		5	
		Proportion of production sold fresh	4		5	
		Sanitation	5		4	
		Local Support Businesses	4		4	
		Availability of Support Businesses	5		4	
		Proportion of feed ingredients sourced from socially responsible sectors	3	4.40	5	4.30
	Post-Harvest Assets	Borrowing Rate Compared to Risk-free Rate	3		3	
		Source of Capital	4		3	
		Age of Facilities	4	3.67	3	3.00
	Processing Managers	Earnings Compared to Regional Average Earnings	5		3	
		Manager Wages Compared to Non-fish farming Wages	5		3	
		Education Access	5		5	
		Access to Health Care	5		5	
		Social Standing of Processing Managers	4		3	
		Nonresident Ownership of Processing Capacity	3	4.50	5	4.00
	Processing Workers	Earnings Compared to Regional Average Earnings	3		2	
		Worker Wages Compared to Non-fish farming Wages	3		3	
		Education Access	5		5	
		Access to Health Care	5		5	
		Social Standing of Processing Workers	2		2	
		Proportion of Nonresident Employment	2		5	
		Worker Experience	4	3.43	4	3.71

Supplementary Table 4. Aquaculture Performance Indicators - Inputs

Indicator	Metric	Norwegian Atlantic Salmon		Florida Cedar Key Clams	
National Environment	Environmental Performance Index (EPI)	5	5.00	5	5.00
Exogenous Factors	Natural Disasters and Catastrophes	4		5	
	Drought	5		5	
	Pollution Shocks and Accidents	5		5	
	Level of Chronic Pollution - Production Effects	5		4	
	Level of Chronic Pollution - Consumption Effects	5	4.80	4	4.60
Governance	Governance Quality	5		5	
	Governance Responsiveness	5	5.00	4	4.50
Economic Conditions	Index of Economic Freedom	5		5	
	Gross Domestic Product (GDP) Per Capita	5	5.00	5	5.00
Land Rights	Proportion of Production with Property or Lease Right	5		5	
	Transferability Index	4		4	
	Security Index	4		5	
	Durability Index	5		4	
	Flexibility Index	3		3	
	Exclusivity Index	3	4.00	4	4.17
Collective Action	Proportion of Farmers in Industry Organizations	5		4	
	Farmer Organization Influence on Management	3		2	
	Farmer Organization Influence on Business & Marketing	3	3.67	2	2.67
Participation & Support	Days in Stakeholder Meetings	5		2	
	Industry Financial Support for Management	2	3.50	3	2.50
Leadership & Cohesion	Leadership	3		2	
	Social Cohesion	4	3.50	5	3.50
Gender	Business Management Influence	1		2	
	Resource Management Influence	2		3	
	Labor Participation in Production Sector	2		2	
	Labor Participation in Processing Sector	4	2.25	1	2.00
Management Inputs	Management Expenditure Compared to Farm-Gate Value	4		4	
	Enforcement Capability	5		5	
	Management Jurisdiction	4		5	
	Generations separated by selective breeding	5		2	
	Coordination of regulatory authorities	4		4	
	Level of Subsidies	5		5	
	Percentage of marine ingredients	3		5	
	Traceability of feed inputs	3		1	
	R&D	5		5	
	Private R&D	3	4.10	1	3.70
Data	Biological data collection	4		3	
	Market and economic data	4	4.00	2	2.50
Management Methods	Regional disease control	5		5	
	Genetic management	3		4	
	Discharge/effluent control	3		1	
	Antibiotic use	5		5	
	Antibiotic use practices	5		5	
	Food safety services	5		5	
	Animal welfare/handling practices	5		5	
	Damage compensation/management	5		2	
	Access to Water	5		4	
	Land or water zoning/management	5	4.60	4	4.00

Supplementary Table 4 (continued). Aquaculture Performance Indicators – Inputs

Indicator	Metric	Norwegian Atlantic Salmon		Florida Cedar Key Clams	
Markets & Market Institutions	Transparency of Farm-gate price	4		1	
	Availability of Farm-gate Price & Quantity Information	4		2	
	Number of Buyers	4		4	
	Degree of Vertical Integration	4		3	
	Level of Tariffs	3		5	
	Level of Non-tariff Barriers	3		4	
	Contribution to Economy	2		3	
	Production under contract farming	2	3.25	1	2.88
Infrastructure	International Shipping Service	4		4	
	Road Quality Index	4		5	
	Technology Adoption in Production	5		3	
	Technology Adoption in Processing	5		3	
	Extension Service	3		5	
	Reliability of Utilities/Electricity	5		4	
	Access to Ice & Refrigeration	5	4.43	5	4.14
Scale	Scale of farm	4	4.00	2	2.00
Integrated culture	Integrated culture	1	1.00	1	1.00

Supplementary Table 5. Aquaculture case studies (n=57)

**Case Study**	**Production (mt)**	**Lead scorer**	**Funding**
Bangladesh carp polyculture	1,468,516	M. Dey	Unfunded
Bangladesh pangasius	458,307	M. Dey	Unfunded
Bangladesh tilapia	390,559	M. Dey	Unfunded
Brazil shrimp	650,000	R. Pincinato	Unfunded
Brazil tambaqui	140,000	R. Pincinato	Unfunded
Brazil tilapia	270,000	R. Pincinato	Unfunded
Canada salmon	118,630	F. Asche, R. Tveteras	NFR
Chile mussels	379,096	C. Chávez	SIDA/FONDAP
Chile pelillo	15,869	C. Chávez	SIDA/FONDAP
Chile salmon	701,984	F. Asche, R. Tveteras	NFR
China bighead carp	3,130,301	L. Liu	Unfunded
China common carp	2,896,669	L. Liu	Unfunded
China crayfish	2,089,604	L. Liu	Unfunded
China crispy grass carp	50,000	L. Liu	Unfunded
China crucian carp	2,748,519	L. Liu	Unfunded
China grass carp	5,533,083	L. Liu	Unfunded
China Pacific oyster	1,183,979	L. Liu	Unfunded
China Qianjiang crayfish	143,800	B. Che	Unfunded
China silver carp	3812899	L. Liu	Unfunded
China tilapia	1,231,162	L. Liu	Unfunded

China whiteleg shrimp	1,815,550	L. Liu	Unfunded
China Zhanjiang shrimp	181,790	B. Che	Unfunded
Costa Rica shrimp	1,010	N. Valverde	SIDA
Costa Rica tilapia	5,624	N. Valverde	SIDA
Denmark rainbow trout	32,730	R. Nielsen	Unfunded
Ecuador shrimp	679,985	J. Anderson	Unfunded
Greece seabass and sea bream	139,100	J. Guillen, I. Llorente	Unfunded
India carp	5,770,000	P. Sudhakaran	Unfunded
India oysters	555	P. Sudhakaran	Unfunded
India shrimp	800,000	G. Kumar	Unfunded
Indonesia milkfish and shrimp	162,778	R. Nielsen	Unfunded
Indonesia tilapia and whiteleg shrimp	1,931,421	R. Nielsen	Unfunded
Myanmar carp polyculture	1,121,000	K. Fitzsimmons	World Bank
Myanmar extensive 'trap and hold' shrimp	35,694*	K. Fitzsimmons	World Bank
Myanmar intensive shrimp	35,694*	K. Fitzsimmons	World Bank
Myanmar pond-reared tilapia	69,472	K. Fitzsimmons	World Bank
Myanmar soft-shell crab	2,883	K. Fitzsimmons	World Bank
Nigeria catfish	316, 727	N. Chukwuone	SIDA
Norway salmon	1,364,042	F. Asche, R. Tveteras	NFR
Philippines FMA6 mussels and oysters	30,139	J. Flores	World Bank
Philippines FMA6 tilapia	172,024	J. Flores	World Bank
Philippines FMA9 mangrove crab	8,495	J. Flores	World Bank
Philippines FMA9 P. monodon	15,406	J. Flores	World Bank
Philippines milkfish	193,650	J. Flores	World Bank
Philippines seaweed	1,500,000	J. Flores	World Bank
Scotland salmon	203,881	F. Asche, R. Tveteras	NFR
Spain mussels	214,000	J. Guillen, I. Llorente	Unfunded
Spain sea bass and seabream	32,879	J. Guillen, I. Llorente	Unfunded
Sweden blue mussels	1,986	H. Eggert	Unfunded
Tanzania prawns	11000	B. Tibesigwa	SIDA
Thailand Shrimp	383,170	J. Anderson	Unfunded
US catfish	154,580	G. Kumar	Unfunded
US Cedar Key clams	4,089	T. Garlock	Unfunded
US Gulf of Mexico oysters	216	T. Garlock	Unfunded
US Rhode Island oysters	611	J. Anderson	Unfunded
Vietnam pangasius	1,519,000	L. Nguyen	Unfunded
Vietnam shrimp	877,200	L. Nguyen	Unfunded

* The precise production volume could not be distinguished between extensive and intensive shrimp production in Myanmar.
