# Supplementary material for: Environmental, economic, and social sustainability in aquaculture: the aquaculture performance indicators
Source: Nat Commun. 2024 Jun 20;15:5274. doi: 10.1038/s41467-024-49556-8 (PMC11190207; doi:10.1038/s41467-024-49556-8)
Supplement: Supplementary file 2 — Peer Review File [file 41467_2024_49556_MOESM2_ESM.pdf]

nature portfolio

Peer Review File

Environmental, Economic and Social Sustainability in Aquaculture

Open Access This file is licensed under a Creative Commons Attribution 4.0 International License, which permits use, sharing, adaptation, distribution and reproduction in any medium or format, as long as you give appropriate credit to the original author(s) and the source, provide a link to the Creative Commons license, and indicate if changes were made. In the cases where the authors are anonymous, such as is the case for the reports of anonymous peer reviewers, author attribution should be to 'Anonymous Referee' followed by a clear attribution to the source work. The images or other third party material in this file are included in the article's Creative Commons license, unless indicated otherwise in a credit line to the material. If material is not included in the article's Creative Commons license and your intended use is not permitted by statutory regulation or exceeds the permitted use, you will need to obtain permission directly from the copyright holder. To view a copy of this license, visit http://creativecommons.org/licenses/by/4.0/.

Reviewers' Comments:

Reviewer #1:
Remarks to the Author:
This article explores the environmental, economic, and social sustainability of global aquaculture systems using a dataset of 57 aquaculture systems around the world for the period 2020-2021. The 57 aquaculture systems represent 41% of global aquaculture production. The analysis is based on the Aquaculture Performance Indicators (API), which measure the performance of outputs (n=88) and inputs (n=66) across these 57 systems based on a scale of 1-5. The authors use the results of the API analysis to test whether the three pillars of sustainability—economic, environmental, and social—are complementary or competitive. The article shows that the three pillars are generally complementary, indicating that aquaculture systems that are economically sustainable are also socially and environmentally sustainable. The authors note, however, that global aquaculture is heterogenous and that the three pillars do not hold up in all cases. The paper also compares the results of the API with the Fisheries Performance Index (FPI) –developed by the same group at the University of Florida—and shows that the correlation between environmental and economic indicators is lower in the API than the FPI, whereas the correlation coefficients between environmental and social indicators is considerably higher in the API than the FPI. The correlation between the economic and social indicators is slightly higher in the API than the FPI.

Introducing a dataset that can measure and compare different aspects of sustainability across the global aquaculture industry is greatly desired as aquaculture researchers and the aquaculture industry strive to improve sustainable practices. It is also important for showing the degree to which aquaculture out-performs (or under-performs) capture fisheries in terms of sustainability across the three pillars. Despite the merits of such an approach, however, this article requires substantial revision and more information and insight before it can be published in Nature Communications. My comments follow:

General comments pertaining to the API:

1. Information on the API can be found in Anderson et al (2020) in the references: https://fpilab.org/aquaculture-performance-indicators/. From that site, one can contact some of the authors to obtain the API manual. The reader is left with questions about biases in data collection, particularly when expert opinion in needed to score performance outputs and inputs when actual data do not exist. There is no indication of who the scorers are for the API, or what expertise they might have. What is the source of funding for the API data collection? My suggestion is that the API itself needs to be written up and published in a peer-reviewed journal with full access to the data so the results can be replicated or built upon by others for further analysis. Any conflicts of interest also need to be transparent. Such an effort would help clarify how the indicators listed in S1 and S2 are measured. Researchers who are interested in building on the results could also then see how each output and input performance indicator is scored from 1 to 5—what are the criteria in each case for a ranking of 1 versus a ranking of 5? Given the information that the reader can glean about the API at this stage, it seems quite subjective.

2. The API is based on data collected between 2020 and 2021. Are there issues regarding this time that might skew the results? This period represents the COVID-19 years when prices and markets were influenced by the pandemic. Were the data from the FPI collected during the same period, or (as it seems from the text and references) 3-5 years earlier?

3. The 57 aquaculture systems within the API are listed in S.3., and a few questions arise:

a. Nigerian catfish and Tanzania prawns are included, but there are no tilapia systems analyzed for Africa despite the relative importance of these systems for African aquaculture. Why did the API skip over Egypt? Or other tilapia systems in sub-Saharan Africa?

b. China’s carp systems are all listed individually, but many carp systems are polycultures in China. Why are these polyculture systems not represented (see, for example, Chiu, A., Li, L., Guo, S., Bai, J., et al. (2013), Feed and fishmeal use in the production of tilapia and carps in China. Journal of Aquaculture.)
c. Why was a seaweed system chosen for the Philippines but not for China or Indonesia where most seaweed production occurs?
d. It would be useful to list the volume of production in each of these systems in S.3 (e.g., how they add up to 41% of global production). I assume they were measured in wet weight.
e. For Indonesia, why is milkfish and shrimp combined into one category? And similarly, why is tilapia and whiteleg shrimp placed in the same category? I would assume the performance rankings would differ for species within these groupings.

Now moving through the manuscript:

4. The framing of the argument in the introduction seems a bit strange. Reference is made initially to the 3 pillars of sustainability citing the Naylor et al. (2000) paper in Nature, but that paper was specifically focused on whether aquaculture provided a net addition to world fish supplies or a net loss due to the use of wild fish in feeds (an ecological perspective), and it did not directly address economic or social sustainability. In lines 72 to 84, the Naylor (2000) paper is compared to papers written 20 years later when the sustainability performance of aquaculture had evolved. The time inconsistency sticks out here. It might be more constructive to compare the more recent papers on aquaculture performance to the Naylor et al (2021) paper in Nature.

5. In lines 77 to 79, the authors note that “the potential trade-offs between the environment and aquaculture production are commons issues that at least in principle can be addressed with improved governance”. This point is certainly true with respect to the sustainability of capture fisheries such as the Peruvian anchovy that are used to produce fishmeal and fish oil. A big shift in the move away from wild fish in feeds has also been due to the economics of feeds. Prior to 2000, fishmeal made from capture fisheries was relatively inexpensive (and highly nutritious), so it made sense for producers to have high inclusion rates of FM in their feeds. This situation changed in the 2000s when fishmeal prices escalated, and alternative feed sources became more attractive. The market has played a significant role in the industry’s use of wild fish in feeds.

6. With regards to synergies and trade-offs (lines 160 ff): The correlation between environmental and economic pillars for aquaculture was 0.33 (p-value 0.012). The significance of this result is not strong, and the correlation is low at 0.33, which does not inspire great confidence in the result. It is not clear what the reader should take away from this particular result. The other correlations are higher and more significant. It would be useful if the authors could provide some specific illustrations from their data to bring these statistics to light.

7. In general, the authors note that the aquaculture industry is heterogeneous and that a more nuanced interpretation of sustainability is warranted. Again, some specific examples of the heterogeneity would bring out such nuance for the readers. Some of this nuance is brought out on page 14 (lines 223 ff) when comparing, for example, molluscs to salmon. The discussion of salmon itself did not reveal much nuance, despite quite differing experiences in Norway (widely considered to be quite sustainable across all pillars) versus Chile (which has had challenging sustainability issues, particularly related to pathogens, siting, and the misuse of antimicrobials).

8. Related to the previous point, it is unclear how to read and interpret Figure 4. What is the standardized score from 2 to -2? Much more information needs to go into the legend for that figure. For example, how is risk measured? From Table S1, risk encompasses metrics of volatility—but over what period? How is the volatility compared to other segments of the food system?

9. New material is introduced into the conclusion, such as green financing and consumer markets, that

is not backed by evidence in the main body of the paper. Moreover, the final sentence of the conclusion is weak, as it raises the potential for consumer markets to drive sustainability, but then notes that incentives for such behavior do not exist in the Global South where most aquaculture production occurs. I would suggest re-working the conclusion.

10. I found the result of comparable sustainability metrics between freshwater and marine systems to be quite interesting and relevant for policy discussions. More attention to this result, and what underpins this result, would be interesting for scholars and policymakers in this field.

11. The reference section is very comprehensive.

Reviewer #2:
Remarks to the Author:
The authors apply a set of published aquaculture performance indicators to 57 discrete aquaculture systems from across multiple continents/species/product types etc. They utilize an expert judgement based approach to score against three pillars of sustainability and look for potential complimentarity and trade-offs between these pillars, for each system. The paper uses capture fisheries against which discrete aquaculture systems are compared and materializes several key statements about 'sustainability' of these systems - some of which support and some of which contrast existing narratives/perceptions in the academic literature and wider, in society.

I found the paper easy to read and somewhat intuitive, providing a useful high-level viewpoint that may be useful to those considering wider policies for national aquatic food production. In this context, although it is useful and perhaps expected, to compare aquaculture to the fishery (as alternative ways of obtaining aquatic foods), one is farming and the other uses a hunter-gatherer approach, so perhaps not the only comparison that could and should have been presented in the analysis. I would have liked to have seen the data for aquaculture (and fisheries) normalized against other farmed food sectors (e.g. beef, chicken etc) to provide a truer-indication of sustainability of aquatic foods (from aquaculture) and these alternative farmed meat types. This may have assisted in helping to negate some of the more negative views about sustainability of aquaculture in the literature and wider, mentioned in the literature. Through this lens, perhaps aquaculture would not appear as 'controversial' as proposed in the opening statements of the paper? If possible, adding this analysis would strengthen the paper considerably but if I am honest, I don't know whether this request is feasible given potential to apply the API or similar metrics to these terrestrial systems.

The paper may also be strengthened by mention of other sustainability metrics recently proposed for the sector, including in Nature journals (e.g. the One Health Aquaculture approach outlined in Nature Food) and, by referencing of UN/FAO level statements on sector sustainability.

Overall, I enjoyed reading the paper and believe it will make a useful addition to the literature around aquaculture sustainability - particularly if it can be strengthened as proposed above.

Reviewer #3:
Remarks to the Author:
This paper provides, the best of my knowledge, the first application of the Aquaculture Performance Indicators (APIs) – a methodology (based on the Fisheries Performance Indicators) to provide a broad scale assessment of the (global) aquaculture sector. The paper applies the APIs to a global assessment based on an impressively large number of country/sector/species assessments that together enable the authors to make broad scale comparisons of the social, economic and environmental performance of the sector globally. It is novel in its ambitions and analysis. However, while I think the paper offers

an important contribution to understanding the relative sustainability impact of the sector, there are a number of points where the messaging and focus can be improved.

First, the paper begins with a set of statements, based on a short review of the literature, arguing that the literature on aquaculture sees social, environmental and economic objectives as often incompatible. While I recognise the authors cannot review the literature in its entirety, nor report on such a review in a short paper like this, I do think there is a degree of selection bias in the papers reviewed. As an example, the paper cites Naylor et al. 2000 in a rather prominent way at the start of the paper – which does argue that there are key trade offs – but does not really engage with the substantially different argument in the Naylor et al. 2021 paper. Other statements around assumptions that social and economic trade offs are assumed not to exist (line 113) and assumptions of the relationship between companies and social benefits (Lines 88-102), and that it is not obvious that environmental trade offs are inevitable (line 84) are also not clearly supported. It is also not clear whether some authors, such as Belton and Little (as quoted on Line 280) claim in such definitive terms that large corporations benefit at the cost of smallholders.

Furthermore, while I think the authors are right to questions such assumptions, it is not always clear that the literature really makes the claims the authors are saying it does. For instance, the Hilborn et al. 2020 paper is far from a definitive case of showing that mangroves can be protected – nor is it clear what the relation to aquaculture is here. Gephart et al., 2021a; Koehn et al., 2022 show more than aquaculture is performing well in some dimensions. The statements as such risk setting up a series of so called 'straw men'. My suggestion to the authors is to use less definitive statements of conflict vs. complimentarity, and instead focus on the methodological value of using a systematic and comprehensive framework for assessing the three pillars of sustainability at a global scale. This would be convincing enough.

Second, it is not at all apparent (to me at least) why the authors have made the comparison with fisheries and the FPIs. There is of course a clear methodological link between the APIs and FPIs, but it is not clear why the results of both need to be compared. The goal of the paper is clearly to establish the value of the APIs as a framework and how the data can show the variation in sustainability outcomes that can challenge broad (and often poorly supported) perceptions of aquaculture. Why then compare to fisheries? Including this analysis would need a far more convincing justification – alternatively, the comparison may be better positioned in a completely separate paper (that would in fact be of interest in its own right).

Third, and related to the previous point, the paragraph that concludes that largely compares fisheries and aquaculture finishes with the more narrow observation that "from a sustainability perspective, the water bodies make little difference". As currently written, this statement appears a non sequitur in the paragraph – and section as a whole. The section makes a broad comparison between the trade-offs and synergies between fisheries and aquaculture sustainability. The paragraph then starts wit the diversity of aquaculture, turns to the importance of freshwater production and ends with a comparison of freshwater and marine. Given that the authors make the comparison so prominent in the introduction (line 65), I would suggest refocusing this section of the paper on exactly this statement. It is, as the authors well know, an important statement to make and I would be interested to see stronger evidence from the API data in support of this statement.

Fourth, I was intrigued by the international trade section. The authors make various claims for export vs domestic production in high and low income countries – with various claims that export aquaculture outperforms, or is at least no worse than domestic oriented production. There are generally interesting findings. I have three points here:

(1) What I would like to see is some explanation as to how domestic and export oriented production can be distinguished from the results given the growing ambiguity of many sectors around the world (shrimp in many SE Asia countries are orienting towards growing domestic consumption for instance).

(2) I am also not convinced by the comparison with case studies (following my first point); I don't think the statements made in this paper are necessarily more convincing than the bias of a case study approach as now presented. Said differently, I would encourage the authors to avoid such comparisons and instead let the very detailed data speak for itself.

(3) On line 290, the authors argue that " the results support the notion that production for export and domestic markets are from different production systems and are linked to different performance outcomes although seemingly operating under similar governance systems, and that the dynamics are further nuanced by development status". I am not clear what this statement is actually saying because the results are not presented at a high enough level of precision to support this claim. As such, there seems a lot open to interpretation here. The interpretation of the authors is that "The results suggest that market incentives may have the highest potential in obtaining higher sustainability standards". While this may well a fair statement I cannot follow the reasoning the authors use to get to such a claim from the results presented.

Fifth, the authors state in the conclusion of the paper that there is considerable variation in the degree of sustainability in the industry – matching the variation of production systems and species grown around the world. This is a fair statement based on a general observation. But I would like more detail that is currently presented. For instance, Figure 3 in my view does not show a high degree of variation. Quite the contrary, it shows modest variation in both dimensions of the figure. Figure 5 in contrast does show higher variation based on output dimensions. However, there is a lot of information embedded in this figure that is not explained clearly in the text – yes the authors given an impression by species of where performance is better or worse, but it takes some work to try and follow the authors on the broader comparisons of social, economic and environmental that are being drawn out of this figure. This left me thinking whether there are specific trends that should be drawn out of such a complex figure? And overall, what kinds of variation are most prominent from such a figure?

Finally, the final paragraph of the conclusion makes some assertions that the results can be instructive for policy, R&D and informing the role of market incentives for sustainability. I am not sure what this value is exactly. How could, for instance, three claims in the paper on marine vs. freshwater, intensive vs. extensive and domestic vs export lead to clearer advice – or at least a reflection on what can be improved on in policy terms. Such a statement would extend the paper beyond the claim that the APIs provide insight into the variation of the sector to indicate what the key challenges are for the sector in sustainability terms and what, based on the APIs, might be a programme of improvement (where needed).

Responses to reviewer 1's comments on Environmental, Economic and Social Sustainability in Aquaculture: The Aquaculture Performance Indicators

Thank you very much for taking the time to read our paper carefully and for providing us a set of very useful comments and suggestions, which we strongly believe improved the paper. Below we reproduce your comments in plain text and how we addressed your comments and suggestions in *italics*.

This article explores the environmental, economic, and social sustainability of global aquaculture systems using a dataset of 57 aquaculture systems around the world for the period 2020-2021. The 57 aquaculture systems represent 41% of global aquaculture production. The analysis is based on the Aquaculture Performance Indicators (API), which measure the performance of outputs (n=88) and inputs (n=66) across these 57 systems based on a scale of 1-5. The authors use the results of the API analysis to test whether the three pillars of sustainability—economic, environmental, and social—are complementary or competitive. The article shows that the three pillars are generally complementary, indicating that aquaculture systems that are economically sustainable are also socially and environmentally sustainable. The authors note, however, that global aquaculture is heterogenous and that the three pillars do not hold up in all cases. The paper also compares the results of the API with the Fisheries Performance Index (FPI) –developed by the same group at the University of Florida—and shows that the correlation between environmental and economic indicators is lower in the API than the FPI, whereas the correlation coefficients between environmental and social indicators is considerably higher in the API than the FPI. The correlation between the economic and social indicators is slightly higher in the API than the FPI.

Introducing a dataset that can measure and compare different aspects of sustainability across the global aquaculture industry is greatly desired as aquaculture researchers and the aquaculture industry strive to improve sustainable practices. It is also important for showing the degree to which aquaculture out-performs (or under-performs) capture fisheries in terms of sustainability across the three pillars. Despite the merits of such an approach, however, this article requires substantial revision and more information and insight before it can be published in Nature Communications. My comments follow:
General comments pertaining to the API:

1. Information on the API can be found in Anderson et al (2020) in the references: https://fpilab.org/aquaculture-performance-indicators/. From that site, one can contact some of the authors to obtain the API manual. The reader is left with questions about biases in data collection, particularly when expert opinion in needed to score performance outputs and inputs when actual data do not exist. There is no indication of who the scorers are for the API, or what expertise they might have. What is the source of funding for the API data collection? My suggestion is that the API itself needs to be written up and published in a peer-reviewed journal with full access to the data so the results can be replicated or built upon by others for further analysis. Any conflicts of interest also need to be transparent. Such an effort would help clarify how the indicators listed in S1 and S2 are measured. Researchers

who are interested in building on the results could also then see how each output and input performance indicator is scored from 1 to 5—what are the criteria in each case for a ranking of 1 versus a ranking of 5? Given the information that the reader can glean about the API at this stage, it seems quite subjective.

Sorry. The link was a place holder and it will eventually contain a link to the manual. A paper with a closer description of the APIs is under preparation targeting a special issue of Aquaculture Economics and Management, and we have therefore included the indicators as well as the aggregation categories as supplement S1 in the current paper.

As indicated in the methods section the data collection approach has been pragmatic, and all the data is collected by authors of this paper. In supplement table S3 the leader(s) of the scoring team and funding when applicable is listed.

2. The API is based on data collected between 2020 and 2021. Are there issues regarding this time that might skew the results? This period represents the COVID-19 years when prices and markets were influenced by the pandemic. Were the data from the FPI collected during the same period, or (as it seems from the text and references) 3-5 years earlier?

The API assessments were conducted between 2020 and 2021, meaning the reference year of the scores were for 2018 and 2019, and thus were not influenced by COVID-19. This is now noted in the methods section.

The FPI data was collected between 2011 and 2019. We acknowledge that these data were collected during a different time period than the APIs. While this is not ideal, we do not believe it is a significant issue for the analysis as the indicators are assessing structural issues that are not likely to change significantly over relatively short time spans. This is now noted in the methods sections. In addition, it is the only dataset with global coverage of fisheries and aquaculture that has consistent data for the three dimensions of sustainability, and it sets up future research as the two data collection systems hopefully will facilitate comparison of the fisheries and aquaculture sectors also on a finer scale.

3. The 57 aquaculture systems within the API are listed in S.3., and a few questions arise:
a. Nigerian catfish and Tanzania prawns are included, but there are no tilapia systems analyzed for Africa despite the relative importance of these systems for African aquaculture. Why did the API skip over Egypt? Or other tilapia systems in sub-Saharan Africa?

- We recognize explicitly that the 57 aquaculture case studies are not a random sample in the methods section of the revised version of the paper, and now also specify the omission of Egypt even more explicitly. It is not omitted because we did not try. We use a combination of purposive and convenience sampling to collect data on sectors with high relative importance in global aquaculture production, albeit there are sectors that were not within our capacity to assess. While we certainly would have preferred a systematic sampling approach, this was not feasible with the available funds. Rather, as indicated in table S3 we have obtained some support to collect API in different projects while most of the data has been provided voluntarily at the researchers own time.

b. China's carp systems are all listed individually, but many carp systems are polycultures in China. Why are these polyculture systems not represented (see, for example, Chiu, A., Li, L., Guo, S., Bai, J., et al. (2013), Feed and fishmeal use in the production of tilapia and carps in China. Journal of Aquaculture.)

The Chinese production system is highly diverse. The farmers that were interviewed in our cases consistently referred to their pond systems as e.g. grass carp ponds because it was only one species that was sold and as such that was the basis for the activity. Other species were in all cases kept in the ponds for environmental and/or fish health reasons, but were kept by the harvesting crew at the end of the production cycle. The distinction between mono culture and poly culture is difficult in many systems as other species are present in the production system for various purposes. Our categorization is based on whether more than one species is regarded as an end product. This is now discussed in the methods section.

c. Why was a seaweed system chosen for the Philippines but not for China or Indonesia where most seaweed production occurs?

This is due to the same reason mentioned above for our data collection approach in general. While we have researchers in our group with strong links to China and Indonesia, none of them have any expertise associated with the seaweed industries, and were not comfortable with scoring them. As with tilapia in Egypt, we tried to find someone willing to assess the Chinese seaweed industry or a significant part of it, but was not able to.

d. It would be useful to list the volume of production in each of these systems in S.3 (e.g., how they add up to 41% of global production). I assume they were measured in wet weight.

Estimated production volumes (in live weight) were added to Table S3.

e. For Indonesia, why is milkfish and shrimp combined into one category? And similarly, why is tilapia and whiteleg shrimp placed in the same category? I would assume the performance rankings would differ for species within these groupings.

They are in one category because milkfish and shrimp, and tilapia and whiteleg shrimp are commonly cultured together in earthen ponds in Indonesia. Hence, both systems are truly polyculture systems and thus the assessment was conducted for the production systems, not the individual species. According to the evaluators, this was the only way to obtain information about the system as the farmers are not able to separate the production processes.

Now moving through the manuscript:

4. The framing of the argument in the introduction seems a bit strange. Reference is made initially to the 3 pillars of sustainability citing the Naylor et al. (2000) paper in Nature, but that paper was specifically focused on whether aquaculture provided a net addition to world fish supplies or a net loss due to the use of wild fish in feeds (an ecological perspective), and it did not directly address economic or social sustainability. In lines 72 to 84, the Naylor (2000) paper is compared to papers written 20 years later when the sustainability performance of aquaculture had evolved. The time inconsistency sticks out here. It might be

more constructive to compare the more recent papers on aquaculture performance to the Naylor et al (2021) paper in Nature.

Yes, we probably stretched it too far. We have now replaced the Naylor et al. (2000) citation with more recent papers illustrating potential environmental impacts of aquaculture production including Thomas et al. (2017), Henriksson et al. (2018), Ahmed et al. (2019) and Wang et al. (2020). We have also added a reference (Troell et al., 2014), which is more recent but also warn that further development of aquaculture may reduce food security to the very poorest. And the Naylor et al. (2021) paper is referenced later in the paragraph and several times as it has a much more nuanced perspective on the performance of aquaculture than the above papers.

5. In lines 77 to 79, the authors note that "the potential trade-offs between the environment and aquaculture production are commons issues that at least in principle can be addressed with improved governance". This point is certainly true with respect to the sustainability of capture fisheries such as the Peruvian anchovy that are used to produce fishmeal and fish oil. A big shift in the move away from wild fish in feeds has also been due to the economics of feeds. Prior to 2000, fishmeal made from capture fisheries was relatively inexpensive (and highly nutritious), so it made sense for producers to have high inclusion rates of FM in their feeds. This situation changed in the 2000s when fishmeal prices escalated, and alternative feed sources became more attractive. The market has played a significant role in the industry's use of wild fish in feeds.

We added the following "Market incentives can also be important such as how rising prices for fishmeal and fish oil incentivized the industry to reduce fishmeal inclusion rates and explore alternative protein sources (Tacon 2020; Naylor et al. 2021)."

6. With regards to synergies and trade-offs (lines 160 ff): The correlation between environmental and economic pillars for aquaculture was 0.33 (p-value 0.012). The significance of this result is not strong, and the correlation is low at 0.33, which does not inspire great confidence in the result. It is not clear what the reader should take away from this particular result. The other correlations are higher and more significant. It would be useful if the authors could provide some specific illustrations from their data to bring these statistics to light.

We acknowledge in the text that the correlation was low, and we actually think this is an important result as it indicates that the link between the economic performance of an aquaculture producer and the surrounding ecosystem is weak and therefore that the incentives to improve environmental performance for aquaculture producers on average is relatively weak. We have modified the text to more clearly describe why a weaker correlation is expected for aquaculture compared to fisheries and why this may be a challenge.

We have now expanded the paragraph and write " The productivity of capture fisheries is intricately linked to habitat availability and quality, and thus destruction of habitat reduces the abundance and diversity of fish stocks for future capture. When fisheries are properly managed, the tragedy of the commons is mitigated and the long-term economic benefits of healthy fish stocks are reaped by fishers (Costello et al., 2008; Birkenbach et al., 2017;

Asche et al., 2018). In aquaculture, the farmer can influence the productivity of the system, and more so the more closed the production cycle is, and also limit the impact of and dependence on surrounding ecosystem. Therefore, degradation of the habitat through pollution or other means is not as tightly coupled to the farm's future production level. This also suggest that in many systems, aquaculture producers have weak incentives to limit environmental externalities. As shown e.g. by Pincinato et al., (2021), regulations may be the only way to limit some environmental externalities, although introducing market incentives such as ecolabels may also be an option (Bush et al., 2013; Asche et al., 2021)."

7. In general, the authors note that the aquaculture industry is heterogeneous and that a more nuanced interpretation of sustainability is warranted. Again, some specific examples of the heterogeneity would bring out such nuance for the readers. Some of this nuance is brought out on page 14 (lines 223 ff) when comparing, for example, molluscs to salmon. The discussion of salmon itself did not reveal much nuance, despite quite differing experiences in Norway (widely considered to be quite sustainable across all pillars) versus Chile (which has had challenging sustainability issues, particularly related to pathogens, siting, and the misuse of antimicrobials).

It is true that we do not go into details for the specific species, and we believe that is beyond the scope of this paper. We have added "It should here be noted that the scores presented here compare the averages for different species groups. There is also significant variation in performance within each group not shown here. For instance, for salmon Chile has had significant disease and environmental challenges (Quezada and Dresdner, 2017; Quiñones et al., 2019) that has not been present in the other salmon producing countries.", and we hope you find this satisfactory and leave such detailed comparisons to future research.

8. Related to the previous point, it is unclear how to read and interpret Figure 4. What is the standardized score from 2 to -2? Much more information needs to go into the legend for that figure. For example, how is risk measured? From Table S1, risk encompasses metrics of volatility—but over what period? How is the volatility compared to other segments of the food system?

Thank you for your comment. We agree that there were details about the data standardization that were lacking from the figure legend.

There are 88 individual metrics that make up the 19 dimensions shown in Figure 4. This makes it impossible to describe how each is measured in a figure legend. We added the list of API indicators in Supplementary Material. This provides details about each metric, the criteria for assigning a score, and industry examples that the reader can refer to.

The figure legend was revised to "Average output dimension scores for salmon (n=4), tilapia (n=6), carp (n=9), shrimp (n=12), mollusc (n=9), catfish (n=4), and seaweed (n=2). Scores are standardized by row by subtracting the mean and dividing by the standard deviation. Thus, the standardized score reflects the distance from the mean in units of standard deviation. See the Supplementary materials for the individual metrics comprising each dimension."

9. New material is introduced into the conclusion, such as green financing and consumer markets, that is not backed by evidence in the main body of the paper. Moreover, the final sentence of the conclusion is weak, as it raises the potential for consumer markets to drive sustainability, but then notes that incentives for such behavior do not exist in the Global South where most aquaculture production occurs. I would suggest re-working the conclusion.

Yes, on rereading it we agreed that is just fizzled out. We have now deleted the last section, moved the ecolabeling into sustainability discussion in section 2, and tried to strengthen the discussion with elements such as freshwater vs. marine systems as well as poly culture and control with the production processes.

10. I found the result of comparable sustainability metrics between freshwater and marine systems to be quite interesting and relevant for policy discussions. More attention to this result, and what underpins this result, would be interesting for scholars and policymakers in this field.

We have added a new paragraph to the discussion to highlight this result as well as the poly-culture results, and hope this highlight the potential uses of the APIs better.

11. The reference section is very comprehensive.

Thank you.

And thank you for very helpful and constructive comments that we think have significantly improved the paper. We hope you find our responses satisfactory.

Responses to reviewer 2's comments on NCOMMS-23-27062-T, Environmental, Economic and Social Sustainability in Aquaculture: The Aquaculture Performance Indicators

Thank you very much for taking the time to read our paper carefully and for providing us a set of very useful comments and suggestions, which we strongly believe improved the paper. Below we reproduce your comments in plain text and how we addressed your comments and suggestions in *italics*.

The authors apply a set of published aquaculture performance indicators to 57 discrete aquaculture systems from across multiple continents/species/product types etc. They utilize an expert judgement based approach to score against three pillars of sustainability and look for potential complimentarity and trade-offs between these pillars, for each system. The paper uses capture fisheries against which discrete aquaculture systems are compared and materializes several key statements about 'sustainability' of these systems - some of which support and some of which contrast existing narratives/perceptions in the academic literature and wider, in society.

I found the paper easy to read and somewhat intuitive, providing a useful high-level viewpoint that may be useful to those considering wider policies for national aquatic food production. In this context, although it is useful and perhaps expected, to compare aquaculture to the fishery (as alternative ways of obtaining aquatic foods), one is farming and the other uses a hunter-gatherer approach, so perhaps not the only comparison that could and should have been presented in the analysis. 1. I would have liked to have seen the data for aquaculture (and fisheries) normalized against other farmed food sectors (e.g. beef, chicken etc) to provide a truer-indication of sustainability of aquatic foods (from aquaculture) and these alternative farmed meat types. This may have assisted in helping to negate some of the more negative views about sustainability of aquaculture in the literature and wider, mentioned in the literature. Through this lens, perhaps aquaculture would not appear as 'controversial' as proposed in the opening statements of the paper? If possible, adding this analysis would strengthen the paper considerably but if I am honest, I don't know whether this request is feasible given potential to apply the API or similar metrics to these terrestrial systems.

Thank you for your comments. We agree that the comparison of aquaculture and fisheries with terrestrial farmed food sectors is important for understanding how sustainable aquaculture is relative to other animal proteins. We have hopes to develop similar indicators for terrestrial animal production using the same framework. However, the development of comparable indicators for terrestrial farming and collection of data is a long-term aspiration and outside of the scope of this paper. As a start, our data facilitate a general comparison along the three pillars of sustainability between aquaculture and fisheries, and we are working on aligning the more specific indicators.

2. The paper may also be strengthened by mention of other sustainability metrics recently proposed for the sector, including in Nature journals (e.g. the One Health Aquaculture approach outlined in Nature Food) and, by referencing of UN/FAO level statements on sector sustainability.

Thank you for your suggestion. We have added the following to acknowledges some alternative approaches available in the literature (ln. 168):
"Several other sustainability metrics have been proposed for the aquaculture sector including Volpe et al. (2013), FAO (2017) and Stentiford et al., (2020). However, these systems have a relatively specific focus, and do not allow assessment along all three pillars of sustainability."
Overall, I enjoyed reading the paper and believe it will make a useful addition to the literature around aquaculture sustainability - particularly if it can be strengthened as proposed above.
Ok, thanks

Responses to reviewer 3's comments on NCOMMS-23-27062-T, Environmental, Economic and Social Sustainability in Aquaculture: The Aquaculture Performance Indicators

Thank you very much for taking the time to read our paper carefully and for providing us a set of very useful comments and suggestions, which we strongly believe improved the paper. Below we reproduce your comments in plain text and how we addressed your comments and suggestions in *italics*.

This paper provides, the best of my knowledge, the first application of the Aquaculture Performance Indicators (APIs) – a methodology (based on the Fisheries Performance Indicators) to provide a broad scale assessment of the (global) aquaculture sector. The paper applies the APIs to a global assessment based on an impressively large number of country/sector/species assessments that together enable the authors to make broad scale comparisons of the social, economic and environmental performance of the sector globally. It is novel in its ambitions and analysis. However, while I think the paper offers an important contribution to understanding the relative sustainability impact of the sector, there are a number of points where the messaging and focus can be improved.

1. First, the paper begins with a set of statements, based on a short review of the literature, arguing that the literature on aquaculture sees social, environmental and economic objectives as often incompatible. While I recognise the authors cannot review the literature in its entirety, nor report on such a review in a short paper like this, I do think there is a degree of selection bias in the papers reviewed. As an example, the paper cites Naylor et al. 2000 in a rather prominent way at the start of the paper – which does argue that there are key trade offs – but does not really engage with the substantially different argument in the Naylor et al. 2021 paper. Other statements around **assumptions that social and economic trade offs are assumed not to exist** (line 113) and **assumptions of the relationship between companies and social benefits** (Lines 88-102), and that **it is not obvious that environmental trade offs are inevitable** (line 84) are also not clearly supported. It is also not clear whether some authors, such as Belton and Little (as quoted on Line 280) claim in such definitive terms that large corporations benefit at the cost of smallholders.

We do agree that that we may have portrayed parts of the literature too unnuanced, and have tried to moderate the text, but we still maintain and think the literature indicates that there are significant controversies with respect to the sustainability of aquaculture. We have removed the Naylor et al. 2000 reference and added text and more recent and broader set of citations of key environmental impacts of aquaculture, and we do engage more with the broader arguments of Naylor et al. (2021). We have also deleted statements "Hence, the literature suggests that the interaction between social and economic impacts of aquaculture is case specific, and a trade-off does not necessarily exist." And "Hence, it is not obvious that there needs to be competition between the three pillars of sustainability." Rather we have added text throughout the introduction to better support these ideas.

As for the relationship between economic and social tradeoffs, Beveridge et al. (2013) states "Although unsupported by rigorous study, it is hypothesized that aquaculture producers in developing countries tend to target the production of larger-sized fish, aimed at middle-class urban regional and international markets (UNHRC, 2012), presumably in the expectation that

the higher absolute and relative prices such fish command increase profits. It is proposed here that there is a largely unarticulated and increasingly unmet demand for economically accessible fish by poor consumers, who are in the majority in developing countries."

We rewritten this paragraph to more clearly depict potential tradeoffs between social and economic pillars.

From Belton and Little (2011), "astronomical investment costs resulting from the extremely high intensity of the Vietnamese system of Pangasius production now exclude all but the extremely well resourced from market entry." They also say in regards to Bangladesh "the authors question, however, whether FPA projects have contributed to poverty-focused goals, and conclude that share ownership has become concentrated among wealthy shareholders who have been able to assume control over projects' boards of directors, enabling them to pursue agendas which further their aims and evade regulations put in place to prevent accumulation of shares originally allocated to poor and landless participants." They further state that "Those who have managed to accumulate capital from aquaculture production have usually been, at least in relative terms, reasonably well capitalized prior to initiation of their activities...".

However, we remove the Belton and Little citation and revise the text to "This contrasts with specific case studies in the literature suggesting that export-oriented aquaculture results in greater environmental degradation (van Mulekom et al. 2006)."

The statements in relation to the trade-offs are not assumptions, but a summary of some of our key results. We have tried to make this clearer.

Furthermore, while I think the authors are right to questions such assumptions, it is not always clear that the literature really makes the claims the authors are saying it does. For instance, the Hilborn et al. 2020 paper is far from a definitive case of showing that mangroves can be protected – nor is it clear what the relation to aquaculture is here. Gephart et al., 2021a; Koehn et al., 2022 show more than aquaculture is performing well in some dimensions. The statements as such risk setting up a series of so called 'straw men'. My suggestion to the authors is to use less definitive statements of conflict vs. complimentarity, and instead focus on the methodological value of using a systematic and comprehensive framework for assessing the three pillars of sustainability at a global scale. This would be convincing enough.

The Hilborn et al. (2020) paper is cited in reference to the statement about management systems being successful in preventing overfishing of forage fish stocks. We have added a second reference to support the statement about mangroves, FAO (2007). The world's mangroves 1980–2005: A thematic study prepared in the framework of the Global Forest Resource Assessment 2005. FAO Forestry Paper 153, Rome.). The relevance here is that aquaculture development has been one of the major drivers of mangrove depletion particularly in Southeast Asia. We have revised the introduction, removing many of the definitive statements posing tradeoffs between the three pillars and have provided greater context to support the statements and citations used.

2. Second, it is not at all apparent (to me at least) why the authors have made the comparison with fisheries and the FPIs. There is of course a clear methodological link between the APIs and FPIs, but it is not clear why the results of both need to be compared. The goal of the paper is clearly to establish the value of the APIs as a framework and how the data can show the variation in sustainability outcomes that can challenge broad (and often poorly supported) perceptions of aquaculture. Why then compare to fisheries? Including this analysis would need a far more convincing justification – alternatively, the comparison may be better positioned in a completely separate paper (that would in fact be of interest in its own right).

Reviewer 1 suggest some improvements to this discussion that we largely followed, and we hope that this goes at least some way towards providing the justification you are looking for. We think the comparison is interesting because it illustrates one of the most general challenges the API data can be used to address, and it is an important challenge in many markets where sustainability is an important attribute and for the large discussion of how sustainable seafood is provided. But yes, we certainly agree with you that the main goal of the paper is to establish APIs as a framework and what one can use it for. We hope you find this compromise acceptable.

3. Third, and related to the previous point, the paragraph that concludes that largely compares fisheries and aquaculture finishes with the more narrow observation that "**from a sustainability perspective, the water bodies make little difference**". As currently written, **this statement** appears a non sequitur in the paragraph – and section as a whole. The section makes a broad comparison between the trade-offs and synergies between fisheries and aquaculture sustainability. The paragraph then starts wit the diversity of aquaculture, turns to the importance of freshwater production and ends with a comparison of freshwater and marine. Given that the authors make the comparison so prominent in the introduction (line 65), I would suggest refocusing this section of the paper **on exactly this statement**. It is, as the authors well know, an important statement to make and I would be interested to see stronger evidence from the API data in support of this statement.

This paragraph was moved into the following section which was renamed "Production environment, technologies, and species". Additional text was added to discuss this result and is as follows "This likely stems from the result that there are both positive and poor cases of marine and freshwater aquaculture around the globe. The result sheds light on potential dichotomies regarding freshwater and marine aquaculture (Belton et al., 2020; Costa-Pierce et al., 2022) and has important policy implications for future development in the marine environment."

4. Fourth, I was intrigued by the international trade section. The authors make various claims for export vs domestic production in high and low income countries – with various claims that export aquaculture outperforms, or is at least no worse than domestic oriented production. There are generally interesting findings. I have three points here:

(1) What I would like to see is some explanation as to how domestic and export oriented production can be distinguished from the results given the growing ambiguity of many sectors around the world (shrimp in many SE Asia countries are orienting towards growing domestic consumption for instance).

We agree that the line between production of export-oriented and domestic production is somewhat blurred. However, the APIs contain an indicator of the share of production that is exported that goes a long way to address this challenge. To clarify, we added that the APIs contain an indicator that show market orientation and the following text to the methods section "To examine potential differences in domestic and export-oriented aquaculture sectors, we separate the data based on the International Trade metric. Those sectors receiving a score of 1 (virtually no export) and 2 (2-30% export) were classified as domestic and those sectors receiving a score of 4 (60-90% export) and 5 (90-100% export) were classified as export-oriented. Those sectors scoring a 3 (30-60% export) were not included in the analysis."

(2) I am also not convinced by the comparison with case studies (following my first point); I don't think the statements made in this paper are necessarily more convincing than the bias of a case study approach as now presented. Said differently, I would encourage the authors to avoid such comparisons and instead let the very detailed data speak for itself.

Sorry, but we are not entirely sure what you mean here. However, as noted in our response to your first point, the introduction of the paper has been significantly rewritten, and most of the direct comparisons with case study approaches have been removed.

(3) On line 290, the authors argue that " the results support the notion that production for export and domestic markets are from different production systems and are linked to different performance outcomes although seemingly operating under similar governance systems, and that the dynamics are further nuanced by development status". I am not clear what this statement is actually saying because the results are not presented at a high enough level of precision to support this claim. As such, there seems a lot open to interpretation here. The interpretation of the authors is that "The results suggest that market incentives may have the highest potential in obtaining higher sustainability standards". While this may well a fair statement I cannot follow the reasoning the authors use to get to such a claim from the results presented.

As noted in the response to your comment 4.1, the APIs contain a specific indicator indicating the degree to which the production is exported. The specific sentence was more than a bit convoluted though, and has been removed.

5. Fifth, the authors state in the conclusion of the paper that there is considerable variation in the degree of sustainability in the industry – matching the variation of production systems and species grown around the world. This is a fair statement based on a general observation. But I would like more detail that is currently presented. For instance, Figure 3 in my view does not show a high degree of variation. Quite the contrary, it shows modest variation in both dimensions of the figure. Figure 5 in contrast does show higher variation based on output dimensions. However, there is a lot of information embedded in this figure that is not explained clearly in the text – yes the authors given an impression by species of where performance is better or worse, but it takes some work to try and follow the authors on the broader comparisons of social, economic and environmental that are being drawn out of this figure. This left me thinking whether there are specific trends that should be drawn out of

such a complex figure? And overall, what kinds of variation are most prominent from such a figure?

The analysis in Figure 3 is based on aggregating a large number of individual metrics. As this tends to mask difference, we interpret the variation in Figure 3 to be quite significant, and most cases this was supported by statistically significant differences in means.

We assume that the reviewer is referring to Figure 4 rather than Figure 5. Given the large dataset we are working with, it is not possible to describe all the results within the text. We instead highlight a few of most interesting results. E.g., molluscan aquaculture performed well in environmental dimension but not as well in economic dimensions.

With respect to the variation, there were several dimensions where variation across species was higher. These include local ownership, trade, and several of the of environmental dimensions. This text was added to the manuscript " Interestingly, the greatest variation across species groups was observed in the dimension of local ownership, followed by trade, and the environmental dimensions of certification, feed, and water use and effluent." We now note in the discussion that: "and these (…variations…) becomes more pronounced the more disaggregated the measures used are"

6. Finally, the final paragraph of the conclusion makes some assertions that the results can be instructive for policy, R&D and informing the role of market incentives for sustainability. I am not sure what this value is exactly. How could, for instance, three claims in the paper on marine vs. freshwater, intensive vs. extensive and domestic vs export lead to clearer advice – or at least a reflection on what can be improved on in policy terms. Such a statement would extend the paper beyond the claim that the APIs provide insight into the variation of the sector to indicate what the key challenges are for the sector in sustainability terms and what, based on the APIs, might be a programme of improvement (where needed).

This is a point also made by Reviewer 1, and yes, on rereading it we agreed that is just fizzled out. We have now deleted the last section, moved the ecolabeling into sustainability discussion in section 2, and tried to strengthen the discussion with elements such as freshwater vs. marine systems as well as poly culture and control with the production processes.

Thank you for your helpful and constructive comments that we think have significantly improved the paper. We hope you find our responses satisfactory.

Reviewers' Comments:

Reviewer #1:
Remarks to the Author:
Review comments: Garlock et al., Nat Comms, Jan 11 2024

Thank you for the opportunity to review the revised version of the article submitted to Nature Communications, " Environmental, Economic, and Social Sustainability in Aquaculture: The Aquaculture Performance Indicators". For the most part, I am happy with the revisions and the authors' responses to the review comments that I recorded in the first round. I would still like to see a few additions and edits, however, before the paper is published. When publishing work on the results of a new sustainability indicator that could be replicated and enhanced by other researchers in the field, I feel that full transparency is critical. In particular, the authors need to be more transparent on how they selected their sample, and what was not included in the sample. The selection appears somewhat opportunistic and influences the results. Adding more details on what was not included (of importance) would motivate other researchers to add to this effort. I was happy with the supplemental information, but more details on the sampling strategy would add greatly to the main text.

My suggestions for the final submission are as follows, mostly in order of how they appear in the manuscript:

1. I am happy that the full description of the API is now attached in SI.1. When the API is first mentioned on line 92, I would strongly suggest referring to SI.1 so the reader has access to the details of the API while reading the paper.
2. Line 171: Replace "these systems have a relatively specific focus" with "these metrics have a relatively specific focus".
3. Line 177: Replace "but can also be scored by an expert" with "and are scored by an experts in data poor settings (see S2)." It is important for the reader to see who the experts are so s/he can judge the level of expertise and potential bias in scoring.
4. Line 185: The data were collected between 2020-21. In response to my earlier comments, please make a note in the ms that the data collected in this period pertain to production systems in 2018 and 2019 (authors' response to my question). For expert opinion in data poor settings, is the relevant time period 2018-19, or 2020-21? I would think the height of the COVID-19 pandemic would have influenced the economic indicators.
5. Line 187: 79% of the sample is from developing countries. Are you referring to 79% of the 57 systems analyzed, or 79% of global production?

6. A more major point: In this section (lines 174-198), I think it is important to mention the opportunistic element of the sample. This is a comment that also pertains to the methods section (lines 424 ff). The authors now note in the methods section: "The 57 assessments represent a non-random sample where purposive sampling 427 was used to collect data on sectors with relatively high importance in global aquaculture 428 production; however, there were important sectors that were not within our capacity to assess." They then indicate that Egypt (one of top 10 producing countries) is not included. I would provide a few other examples that seem important here. First, seaweed represents 3% of the sample, but in live-weight production, seaweed is more important. The only seaweed system in the sample is the Philippines, which is dwarfed by China and Indonesia (not included in the sample). Sub-Saharan Africa, which is small in global production, is hardly represented in the sample. While I understand that it is small, some other small sectors (US oysters) are included in the sample. Obviously the results of the indicators depends largely on which systems are included in the sample. I would like more transparency here on how the author group arrived at this sample of 57, and what could be done with future research to expand the sample to be more representative. I am not saying that the results on the indicators are wrong or will change dramatically as more systems are included, but I think it's very important to explain how the sample was constructed.
7. On this note, I am still not satisfied with how Indonesia is represented, with two polyculture

systems: shrimp and milkfish/tilapia. Please note that the total production of these systems is very large in Indonesia according to S2 (162,788 for milkfish/shrimp and 1,931,421 for tilapia/whiteleg shrimp) so the results could really influence the average represented for polyculture/monoculture systems and by species. I have worked in Indonesia and reached out to experts on Indonesian aquaculture. The response I received was as follows:
"Shrimp systems: The very low productivity systems in Indonesia (extensive, or as they call it in Indonesia, 'traditional') sometimes include polyculture, although by % traditional production represents around 10% of national production tonnage. These are mostly farms where the ponds are secondary sources of income and are often minimally tended or managed, meaning polyculture is present due to the nature of stocking and accepted as a way to diversify products. Numerically, government stats show that these make up the majority of farms when considering the total number of farms (irrespective of total tonnage or total area of production). Farms have also been rapidly intensifying over the last 5 years.
So… if the authors are talking about extensive/traditional systems, especially like those in Kalimantan, where farms are largely disconnected from commodity shrimp supply chains (hatcheries, feed mills, processors, exporters) and dominated by P. monodon and other native/non-P. vannemei shrimp species cultivation, their assumptions might be reasonable. But they would not be reasonable for shrimp aquaculture across Indonesia as a whole or where the sector is headed."
I am still not satisfied with how the authors have treated Indonesian systems (these polycultures vs monocultures for shrimp and tilapia; the lack of seaweed).

8. Line 209: Coming back to the relationship between economic and environmental scores that were addressed in my first set of comments. I understand the point that the authors are making regarding the role that the environment plays in provisioning fisheries and supporting aquaculture, so that's okay. When I examine Figure 2, however, I see that the trend line is influenced by some sample observations that have a high environmental score relative to the economic score, which seems to be a different point worth mentioning in the manuscript.
9. Line 239: The second phrase of this sentence "and as such…" does not seem to follow from the first phrase. I suggest splitting into two sentences or revising the sentence.
10. Figure 3: Why is seaweed/algae missing from this figure? Seaweed was 3% of the sample, and different species of algae rank very high in global live-weight volume. Many observers have high hopes for seaweed as a sustainable blue food source (I am more skeptical). The authors should be explicit about what they have learned about seaweed/algae, especially since the major producers (China, Indonesia) are missing.
11. In the trade section (line 327 ff), how is the Indonesian shrimp/tilapia and shrimp/milkfish handled? Most of the shrimp is exported, and the tilapia/milkfish are consumed domestically. Since these are large numbers, I'm wondering how these polycultures are handled vs the carp systems (many of which are polycultures) in China. (One of author responses to my comments seemed to be dealing with Chinese carp differently by highlighting one species in the system.)
12. Paragraph starting on line 376: Check the tenses on the verbs—not all correct here.
13. Line 378: Debate would be clearer if edited as "relative merits and potential of freshwater versus marine aquaculture".
14. Line 387: Change "the" analysis to "Our" analysis for clarity.
15. Line 388: Edit to: "The analyses highlight and compare different aspects of sustainability for different forms of aquaculture, and can be used to identify areas for improvement and key areas of needed research." Reason: I don't think the analysis and paper, as currently drafted, really identifies areas for improvement and key areas of needed research.

Signed: Roz Naylor

Reviewer #2:
None

Reviewer #3:
Remarks to the Author:
The authors have addressed my comments.

The introduction is now more tempered in its claims on social, economic and environmental impacts and trade-offs based on the literature. And the the additional edits to the other sections of the paper also improve the clarity of argumentation.

I am still left with some questions on why the comparison of the FPis is necessary. But on balance, the FPIs do provide a point of comparison. The authors do little with this comparison, but the point that aquaculture performs as least as well (and often better) than fisheries is important to make in a paper like this.

The conclusion of the paper is improved - with a statement around the usefulness of the APIs for improving the performance of the sector.

Responses to reviewer 1 on Environmental, Economic and Social Sustainability in Aquaculture: The Aquaculture Performance Indicators

Thank you again for taking the time to read our paper carefully and for providing us a set of very useful comments and suggestions, which we strongly believe improved the paper. Below we reproduce your comments in plain text and how we addressed your comments and suggestions in *italics*.

I was happy with the supplemental information, but more details on the sampling strategy would add greatly to the main text.

My suggestions for the final submission are as follows, mostly in order of how they appear in the manuscript:

1. I am happy that the full description of the API is now attached in SI.1. When the API is first mentioned on line 92, I would strongly suggest referring to SI.1 so the reader has access to the details of the API while reading the paper.

SI.1 is now referred to on line 92 when the API is first mentioned.

2. Line 171: Replace "these systems have a relatively specific focus" with "these metrics have a relatively specific focus".

Done.

3. Line 177: Replace "but can also be scored by an expert" with "and are scored by an experts in data poor settings (see S2)." It is important for the reader to see who the experts are so s/he can judge the level of expertise and potential bias in scoring.

Replaced

4. Line 185: The data were collected between 2020-21. In response to my earlier comments, please make a note in the ms that the data collected in this period pertain to production systems in 2018 and 2019 (authors' response to my question). For expert opinion in data poor settings, is the relevant time period 2018-19, or 2020-21? I would think the height of the COVID-19 pandemic would have influenced the economic indicators.

This have now been clarified on lines 186-188 as the sentence is rewritten to "Data on 57 aquaculture systems were collected between 2020 and 2021 that reflects performance of production systems as well as more general economic indicators in 2018 and 2019. As such, the data is not impacted by the COVID-19 pandemic and associated measures"

5. Line 187: 79% of the sample is from developing countries. Are you referring to 79% of the 57 systems analyzed, or 79% of global production?

We have tried to clarify this by writing: "Seventy-nine percent of the case studies are from developing countries" This is now in ln 196.

6. A more major point: In this section (lines 174-198), I think it is important to mention the opportunistic element of the sample. This is a comment that also pertains to the methods section (lines 424 ff). The authors now note in the methods section: "The 57 assessments represent a non-random sample where purposive sampling 427 was used to collect data on sectors with relatively high importance in global aquaculture 428 production; however, there were important sectors that were not within our capacity to assess." They then indicate that Egypt (one of top 10 producing countries) is not included. I would provide a few other examples that seem important here. First, seaweed represents 3% of the sample, but in live-weight production, seaweed is more important. The only seaweed system in the sample is the Philippines, which is dwarfed by China and Indonesia (not included in the sample). Sub-Saharan Africa, which is small in global production, is hardly represented in the sample. While I understand that it is small, some other small sectors (US oysters) are included in the sample. Obviously the results of the indicators depends largely on which systems are included in the sample. I would like more transparency here on how the author group arrived at this sample of 57, and what could be done with future research to expand the sample to be more representative. I am not saying that the results on the indicators are wrong or will change dramatically as more systems are included, but I think it's very important to explain how the sample was constructed.

On ln 191 we now write: "The 57 case studies are an opportunistic sample targeting the major species and countries where aquaculture production takes place, but limited by where researchers volunteered to conduct APIs." And on ln 418 at the end of the conclusions section we write "An expansion of the database will be beneficial as it will allow analysis of specific sub-systems, and also make the sample even more representative. In particular, adding more observations for seaweed would facilitate analysis of the most rapidly growing parts of the aquaculture sector.". We also add several clarifications and in the methodology section we note explicitly: "For comparison, global aquaculture production is 47% finfish, 28% seaweed, 15% mollusks, and 9% crustaceans. Hence, seaweed aquaculture is the species group most underrepresented in our sample and with only two observations, a separate analysis could not be conducted for this sector. However, it is worthwhile to note that as most of the seaweed production has a low unit value, its value share is only 5%. It is also important to note that some small sectors are present largely because the data was easy to obtain. The main results are quite stable as removal of outliers does not change conclusions (ln 462)" Please see later in this report for further discussion of the stability of the results. Yes, we have only two observations from Sub-Saharan Africa but we think they represent that part of the global aquaculture system reasonably well given its limited production.

7. On this note, I am still not satisfied with how Indonesia is represented, with two polyculture systems: shrimp and milkfish/tilapia. Please note that the total production of these systems is very large in Indonesia according to S2 (162,788 for milkfish/shrimp and 1,931,421 for tilapia/whiteleg shrimp) so the results could really influence the average represented for polyculture/monoculture systems and by species. I have worked in Indonesia and reached out to experts on Indonesian aquaculture. The response I received was as follows:
"Shrimp systems: The very low productivity systems in Indonesia (extensive, or as they call it in Indonesia, 'traditional') sometimes include polyculture, although by % traditional production represents around 10% of national production tonnage. These are mostly farms where the ponds are secondary sources of income and are often minimally tended or

managed, meaning polyculture is present due to the nature of stocking and accepted as a way to diversify products. Numerically, government stats show that these make up the majority of farms when considering the total number of farms (irrespective of total tonnage or total area of production). Farms have also been rapidly intensifying over the last 5 years.
So… if the authors are talking about extensive/traditional systems, especially like those in Kalimantan, where farms are largely disconnected from commodity shrimp supply chains (hatcheries, feed mills, processors, exporters) and dominated by P. monodon and other native/non-P. vannemei shrimp species cultivation, their assumptions might be reasonable. But they would not be reasonable for shrimp aquaculture across Indonesia as a whole or where the sector is headed."
I am still not satisfied with how the authors have treated Indonesian systems (these polycultures vs monocultures for shrimp and tilapia; the lack of seaweed).

We are aware that the Indonesian sector is rapidly changing. Our observation was collected for 2018, and as such before the intensification wave. In principle, this can be accounted for by dividing the sector into two parts and score respectively the traditional extensive and the intensive separately. However, as we do not have that data, that must be conducted at a future date. We do note on ln 493: "The focus of a specific year can be a challenge when a sector is rapidly changing. For instance, when our observation for shrimp in Indonesia was conducted, the industry was primarily composed of extensive producers, while it has intensified rapidly since then." and on ln 513 "Indonesian tilapia/shrimp system, the shrimp is mostly exported while the tilapia is consumed locally. Production indicators are then an average of the two species. For example, the export indicator is scored as a 3."

As a check of stability, we remove the two Indonesian polycultures and re-run the comparisons between monoculture and polycultures as well as comparisons across species groups. We find minor changes in coefficient value, but the overall significance does not change when comparing monocultures to polycultures.
In particular, when the observations for Indonesia is removed monocultures still scored higher than polycultures on all three pillars: environmental ($p=0.089$), economic ($p=0.022$), and community ($p=0.010$). The correlations for environmental-economic pillars decline to 0.23 from 0.33 but is still statistically significant, environmental community declined to 0.44 from 0.45, and economic and community declined to 0.53 from 0.55.

If we assume that our data is for extensive culture and adjust the production numbers to approximately 10% of the Indonesian production, this will lower our coverage from 41% to 40% of global aquaculture production, but it will not change any of the averages because they are not weighted. And given that our data collection was conducted for 2018, this is most likely too large of a reduction as the extensive sector was relatively larger back then.

8. Line 209: Coming back to the relationship between economic and environmental scores that were addressed in my first set of comments. I understand the point that the authors are making regarding the role that the environment plays in provisioning fisheries and supporting aquaculture, so that's okay. When I examine Figure 2, however, I see that the trend line is influenced by some sample observations that have a high environmental score relative to the economic score, which seems to be a different point worth mentioning in the manuscript.

To investigate the stability of the results, we removed four outliers and re-computed the correlation between economic and environmental. The coefficient increased from 0.33 to 0.48 and remained statistically significant. The outliers were India oysters, India carp, Myanmar carp polyculture, and Myanmar extensive shrimp. On ln 244 we now note that: "However, it is worthwhile to note that the weaker correlation is largely driven by four outliers, India oysters, India carp, Myanmar carp polyculture, and Myanmar extensive shrimp. If these are removed, the correlation coefficient increases to 0.48."

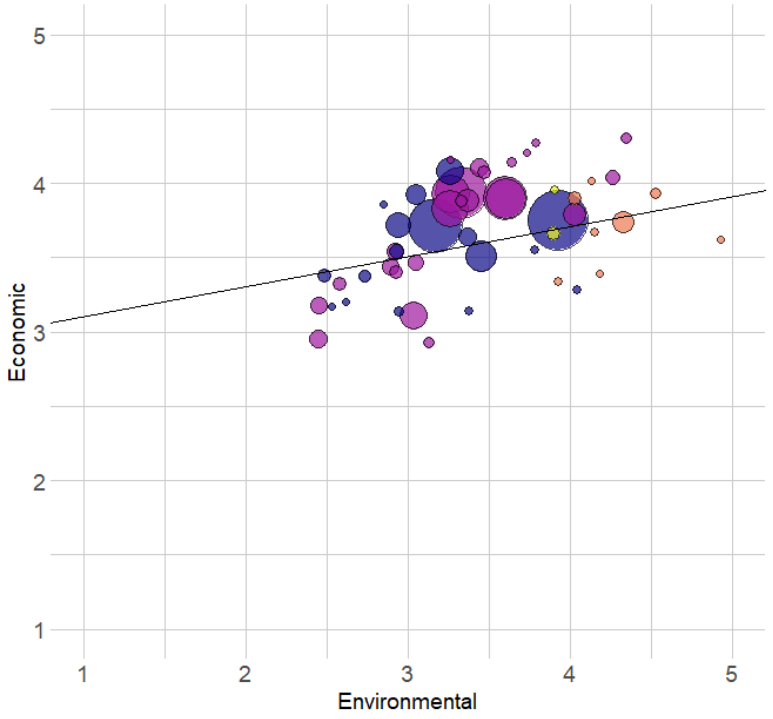

9. Line 239: The second phrase of this sentence "and as such…" does not seem to follow from the first phrase. I suggest splitting into two sentences or revising the sentence.

The two sentences were split into two.

10. Figure 3: Why is seaweed/algae missing from this figure? Seaweed was 3% of the sample, and different species of algae rank very high in global live-weight volume. Many observers have high hopes for seaweed as a sustainable blue food source (I am more skeptical). The authors should be explicit about what they have learned about seaweed/algae, especially since the major producers (China, Indonesia) are missing.

We only have two seaweed observations in the database, and that is too few to compute any meaningful aggregate measures.

11. In the trade section (line 327 ff), how is the Indonesian shrimp/tilapia and shrimp/milkfish handled? Most of the shrimp is exported, and the tilapia/milkfish are consumed domestically. Since these are large numbers, I'm wondering how these polycultures are handled vs the carp systems (many of which are polycultures) in China. (One of author responses to my

comments seemed to be dealing with Chinese carp differently by highlighting one species in the system.)
Both Indonesian systems scored a 3 on the international trade metric indicative of both domestic and international markets and thus were excluded from the trade analysis. In addition, on line 512 we note that "In the Indonesian tilapia/shrimp system, the shrimp is mostly exported while the tilapia is consumed locally. Production indicators are than an average of the two species. For example, the export indicator is scored as a 3.".

12. Paragraph starting on line 376: Check the tenses on the verbs—not all correct here.

Revised

13. Line 378: Debate would be clearer if edited as "relative merits and potential of freshwater versus marine aquaculture".

Done.

14. Line 387: Change "the" analysis to "Our" analysis for clarity.

Done.

15. Line 388: Edit to: "The analyses highlight and compare different aspects of sustainability for different forms of aquaculture, and can be used to identify areas for improvement and key areas of needed research." Reason: I don't think the analysis and paper, as currently drafted, really identifies areas for improvement and key areas of needed research.

Done.

Thanks again for your helpful comments, and we hope you will find our responses satisfactory.

Reviewers' Comments:

Reviewer #1:
Remarks to the Author:
Really nice job on this paper. I appreciate your responses to all of my comments. I'm now ready to sign off. This paper will be a valuable contribution to the literature, and I hope it sparks efforts to measure additional important aquaculture systems.

Responses to reviewer 1 on Environmental, Economic and Social Sustainability in Aquac ulture: The Aquaculture Performance Indicators

Really nice job on this paper. I appreciate your responses to all of my comments. I'm now ready to sign off. This paper will be a valuable contribution to the literature, and I hope it sparks efforts to measure additional important aquaculture systems.

Thank you
